# Exploring the traditional Chinese diet and its association with health status—a systematic review

**DOI:** 10.1093/nutrit/nuae013

**Published:** 2024-08-02

**Authors:** Jizhao Niu, Bai Li, Qing Zhang, Ge Chen, Angeliki Papadaki

**Affiliations:** Centre for Exercise, Nutrition and Health Sciences, School for Policy Studies, University of Bristol, Bristol, UK; Centre for Exercise, Nutrition and Health Sciences, School for Policy Studies, University of Bristol, Bristol, UK; School of Psychological Science, University of Bristol, Bristol, UK; Centre for Exercise, Nutrition and Health Sciences, School for Policy Studies, University of Bristol, Bristol, UK; Bristol Dental School, University of Bristol, Bristol, UK; Centre for Exercise, Nutrition and Health Sciences, School for Policy Studies, University of Bristol, Bristol, UK

**Keywords:** Chinese diet, definition, noncommunicable diseases, systematic review, traditional diets

## Abstract

**Context:**

Increased adherence to a traditional Chinese diet (TCD) could reduce the increasing prevalence of noncommunicable diseases. Currently, there is no consistent definition of the TCD in the literature, and its associations with health outcomes have not yet been identified.

**Objective:**

This systematic review aimed to assess the definition of the TCD, in the literature, and to evaluate whether the TCD, as described, is associated with health outcomes.

**Data sources:**

Fourteen databases were searched up to April 25, 2022.

**Data extraction:**

Three reviewers (in pairs) independently screened and extracted data. A modified risk-of-bias tool was used to assess the quality of the studies assessing the TCD definition; the Newcastle–Ottawa Scale and the Cochrane Risk-of-Bias tool were used to assess the quality of the observational studies and randomized controlled trials assessing associations between the TCD and health outcomes.

**Data analysis:**

Ninety-nine studies were identified that assessed the TCD definition. In at least 75% of the studies, rice and leafy vegetables were consistently reported as food groups that characterize the TCD; the most frequently cited food items were white rice, spinach, bokchoy, and cabbage. Fish and seafood, pork, and pork products were consistently reported in studies exclusively referring to the TCD consumed in southern China (n = 21 studies), whereas wheat and wheat products were commonly reported in studies focusing on northern China (n = 14 studies). Fifteen studies reported on the quantities of food groups that are characteristic of the TCD, but their findings were inconsistent. Of the 99 studies, 54 assessed associations with health outcomes. The TCD was overall inversely associated with obesity risk and weight gain, while relationships between the TCD and other health outcomes were inconsistent.

**Conclusion:**

Further studies are needed to determine the quantities of foods consumed in the TCD and to establish a consistent definition for further exploration of the TCD’s potential role in preventing non-communicable diseases.

## INTRODUCTION

Noncommunicable diseases (NCDs), such as cardiovascular diseases (CVDs), cancers, chronic respiratory diseases, and diabetes, have major implications on a global scale.^1^^,^^2^ In the past decade, approximately 41 million people died from NCDs each year, accounting for 71% of all global deaths.^3^ Additionally, global data from 2016 indicate that NCDs were responsible for 61.4% of disability-adjusted life years.^4^ It has been suggested that China contributes the largest number of patients and deaths related to NCDs,^5^ and NCDs have become the biggest health challenge to China, accounting for 89% of its 9.98 million annual deaths.^6^ A World Bank report estimated that morbidity and mortality attributable to NCDs are expected to rise by approximately 50% and 80%, respectively, in China, by 2030.^7^

Poor diet is globally regarded as a primary risk factor for NCDs.^8^ To reduce the increasing prevalence of NCDs, traditional dietary patterns have been recommended by several international organizations.^9^^,^^10^ These diets are commonly regarded as healthy, because they include a large amount of plant-based foods, such as vegetables, fruits and grains, as well as a small amount of foods of animal origin and oils.^11^^,^^12^ For example, a systematic review and meta-analysis of controlled trials has indicated that the Mediterranean diet is associated with reduced risk of a multitude of NCDs, such as CVDs, stroke, and breast cancer,^13^ so it should be promoted as a healthy dietary pattern. However, due to cultural and geographical influences, it may not be feasible to promote the Mediterranean diet in all countries.

China has undergone major socio-economic transitions over the past decades, in parallel with important transitions in food supply and dietary habits.^14^ The Chinese diet is being gradually Westernized, with a reduction in the consumption of vegetables and whole grains, and an increase in the intake of refined grains, red meat, and unhealthy fats, which has been suggested to have contributed to the increasing prevalence of NCDs in the country.^15^ Therefore, promoting the TCD might be an important public health measure for addressing the high rates of NCDs in China.^16^ However, there are various definitions of the TCD in the existing literature, potentially due to the diverse eating habits and cultural traditions across Chinese regions, and the significant changes to food habits and food culture over time.^17^^,^^18^ These diverse definitions make it challenging to establish the association between the TCD and health outcomes.^18^ Currently, no consistent definition of the TCD has been achieved. Additionally, no study has systematically evaluated the definition of the TCD and its association with health outcomes in the existing literature.

The aim of this study was, therefore, to systematically assess how the TCD is defined in the existing literature, and to evaluate the association between the TCD, as defined in the literature, with health outcomes. The specific objectives were: (1) to identify the food groups and food items that were consistently identified as components of the TCD, as well as the frequency of consumption and amount consumed; and (2) to synthesize and appraise the published evidence on the association between the TCD and health outcomes.

## METHODS

This review was conducted based on a registered protocol (PROSPERO registration number: CRD42022321637), and reported following the PRISMA (Preferred Reporting Items for Systematic Reviews and Meta-Analyses) guidelines (Supplementary Materials I, see Table S1 in the Supporting Information online).^19^

### Search strategy

Searches were performed to identify peer-reviewed articles, books, and gray literature (such as conference abstracts, and reports) published in English and/or Chinese, up to April 25, 2022, without any limitations in terms of the publication date or place. Local chronicles (like Di Fang Zhi) were not considered eligible for inclusion, because they focus more on the history of a single food, not the whole diet. Databases searched were Anthropology Plus, PubMed, CINAHL, MEDLINE, Embase, Web of Science, ProQuest (Dissertations and Theses), PsycINFO, Scopus, CENTRAL and Cochrane Reviews, China National Knowledge Infrastructure (CNKI), Wanfang Data, VP Website, and SinoMed (CBM). The search strategy was designed by J.N., with consultation with B.L. and A.P., and tailored to each database; the detailed search strategy is provided in Supplementary Materials I; see Table S2 in the Supporting Information online.

### Study eligibility criteria

This systematic review applied PICOS (Population, Intervention, Comparison, Outcome, and Study design)^20^ criteria for each research question, as detailed in Table 1. Studies and articles describing diet as “traditional” Chinese were considered eligible, including those conducted outside of China, as long as they referred to diets distinctive of China. Articles that discussed a specific geographical area of China and those focused on the traditional diet of a minority group were included as well. Studies and articles were excluded if: (1) they only focused on single foods or nutrients instead of the whole dietary pattern; (2) they did not identify a “traditional” diet; and (3) they were duplicate studies (such as articles by different authors containing information on the same study or project).

**Table 1:** PICOS criteria for inclusion of studies

Parameter	Inclusion criteria for the first research question (assessment of the TCD definition)	Inclusion criteria for the second research question (assessment of associations between the TCD and health outcomes)
Population	Adults aged over 18 years who consume a traditional Chinese diet, except postpartum women, especially those breastfeeding	Same as research question 1
Intervention/exposure	NA	Dietary pattern labelled as TCD and/or eating habits recognized as traditional Chinese, and/or highest level of adherence to the TCD
Comparison/control	NA	Other types of dietary patterns; no treatment or usual care in terms of diet; lowest level of adherence to the TCD
Outcome	Definition of the traditional Chinese diet	Overall incidence or prevalence of NCDs (such as diabetes, cancer, etc.); metabolic risk factors; BMI and other related factors (such as waist circumference); diabetes risk factors (such as blood concentrations of glucose); other NCD-related outcomes
Study design	All types of studies	Quantitative peer-reviewed studies

*Abbreviations:* BMI, body mass index; NA, not applicable; NCDs, noncommunicable diseases; TCD, Traditional Chinese diet.

### Study selection

Covidence systematic review software (Veritas Health Innovation, Melbourne, Australia) was used for screening the search results. After eliminating the duplicates, the titles and abstracts of identified records were screened by 2 independent reviewers (J.N. and Q.Z.), followed by an independent review of the full texts against the eligibility criteria. Any discrepancy was solved by discussion (inter-rater reliability: Kappa = 0.74; 97% agreement). Consensus was achieved by consulting a third reviewer (A.P. and/or B.L.) if initial agreement could not be achieved.

### Data extraction

First, 2 independent reviewers (J.N. and Q.Z.) conducted a pilot study of the data extraction form (Supplementary Materials I, see Table S3 in the Supporting Information online) with 10% of the studies. Following the pilot, these reviewers proceeded to extract data from the remaining studies. The extracted data included study characteristics (eg, author, country, publication date, and study area), methodological information (eg, study design and study aim), the characteristics of the population (eg, numbers, age, sex, and health status if applicable), the dietary pattern assessment method, and the definition and description of the diet. For the second objective, the intervention/exposure characteristics and descriptions of comparators/controls, outcome measures, quantitative outcomes (such as risk ratios [RRs], odds ratios [ORs], mean value, and prevalence rate), and covariates were also extracted. Any disagreement was resolved by discussions (inter-rater reliability: Kappa = 0.51; 96% agreement); consensus was achieved by consulting a third reviewer (A.P. and/or B.L.) if needed.

### Data analysis for the definition of TCD (review question 1)

The frequency of the citation of food items and food groups was calculated for defining the TCD. All foods and food groups characterizing the TCD were extracted into an Excel document (see Supplementary Materials II in the Supporting Information online) to calculate the frequency of the citation of food items and food groups. Food items reported using different names were categorized into the same food group (for example, corn and maize were categorized into the corn and corn products group). If a study mentioned several food items belonging to one food group, each food item was calculated once and the food group was also only calculated under one citation. For example, if a source reported apples, pears and peaches, each was documented under one citation and the food group “fruits” was only calculated once, not 3 times, under that same citation. The categorization of food groups was based on a food grouping system developed specifically for the Chinese Health Nutrition Survey by researchers from the University of North Carolina at Chapel Hill and the Chinese Institute of Nutrition and Food Safety (INFS)^21^ (which separates foods into nutritionally and behaviorally meaningful food groups), and the Chinese Food Composition Tables (Supplementary Materials I, see Table S4 in the Supporting Information online).^22^

#### Methods used for defining geographical regions when calculating the food citation frequency

Different definitions of the TCD in the existing literature were presented by regions, such as the identified “traditional southern diet” and “traditional northern diet”. These used the Qinling Mountains–Huaihe River line (which has been widely recognized as an important north–south geographical demarcation line^23^) to divide China into northern and southern, in terms of the considerable differences that exist in natural geography, geology, and culture between the northern and southern regions.^24^^,^^25^ In this review, definitions based on this classification were subsequently defined as TCD, traditional southern diet, traditional northern diet, and traditional diet in minority groups. The food citation frequency was calculated and then reported by region (4 subgroups in total). If one study reported more than one regional dietary pattern, the frequency of food items and food groups in each diet were calculated separately.

However, China is a large country, and the differences in diets might not be limited to southern and northern. It was, therefore, deemed appropriate to use an additional classification method in addition to the Qinling Mountains–Huaihe River line, to better capture potential differences between regions and across geographical classification systems. The second approach, named “5-regions classification,” involved dividing China into 5 main areas in terms of dietary preferences and geographical characteristics: northern China, southern China, eastern China, western China, and central China.^22^ The primary reviewer (J.N.) recategorized the definitions into these 5 groups according to where the participants in the included studies came from, or the regions where the studies were conducted. Additionally, data from studies in which participants represented the whole country (ie, could not be classified as being from a single region) were categorized into the TCD group. The food citation frequency based on the recategorized regions (6 subgroups in total) was then calculated and reported.

### Data analysis for associations between TCD and health outcomes (review question 2)

There was significant heterogeneity in the methods, populations, and outcomes used in studies considered eligible to address the second research question, so a narrative synthesis was a more appropriate method for reporting the findings than a meta-analysis.

### Risk of bias, study quality, and quality of reporting

Considering the nature of the studies included in this systematic review for the first research question, the existing tools for assessing risk of bias were not deemed appropriate. Thus, the quality of these studies was assessed using an adapted tool that had previously been used in similar studies in India and Mexico.^12^^,^^26^ This 8-item index assessed whether the studies described: (1) the foods included in the dietary pattern; (2) the food groups included in the dietary pattern; (3) the proportions of the foods and food groups included; (4) the methodology used to identify the dietary pattern; (5) the geographical area(s) the dietary pattern was consumed in; (6) the population represented; (7) the identification of whether the data and descriptions of diet were nationally or regionally representative; and (8) the year(s) represented.

For studies assessing the association between the TCD and health outcomes, risk of bias was evaluated using an adapted version of the Newcastle–Ottawa Scale for cohort, cross-sectional, and case–control studies^27^^,^^28^ (Supplementary Materials I, see Table S5 in the Supporting Information online), while the Cochrane risk-of-bias tool was used for randomized controlled trials (RCTs).^29^ The overall quality of the reporting of cross-sectional, case–control, and cohort studies was also evaluated (by J.N.) using the STROBE statement,^30^ while the quality of reporting of RCTs was assessed via the CONSORT statement.^31^ Three independent reviewers (J.N., Q.Z., and G.C.) assessed risk of bias and discussed discrepancies until agreement was reached.

## RESULTS

**Figure 1:**
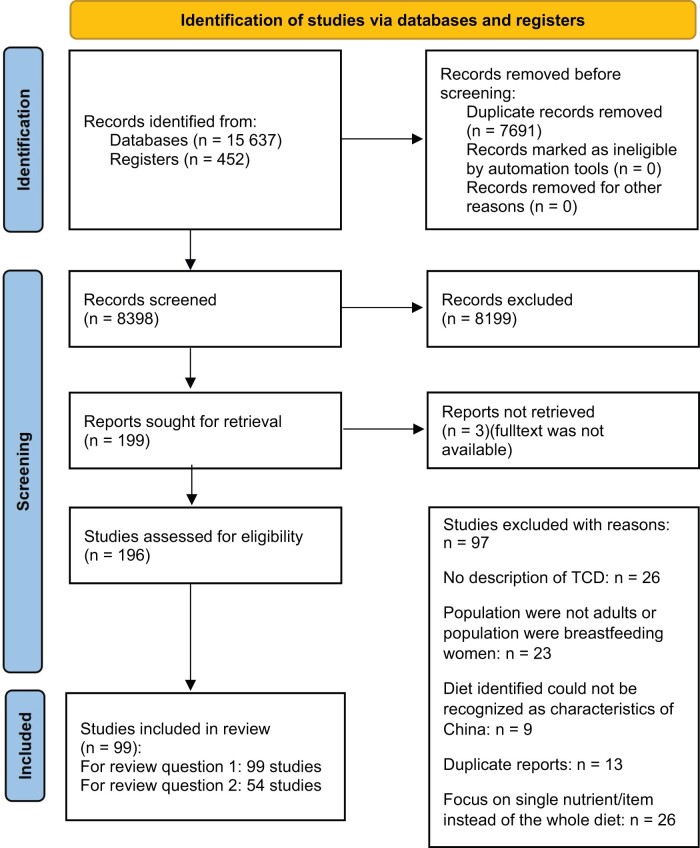
**Flowchart of search and selection process**.

After removing the duplicates, 8398 articles remained for title and abstract screening, of which 8199 were excluded, as they were irrelevant to the review’s aim. As full texts could not be found for 3, the full text of a total of 196 studies was read, and 97 further studies were excluded (see detailed reasons in Supplementary Materials I [Table S6] in the Supporting Information online). Ultimately, a total of 99 studies were included, with all 99 reporting on the definition of the TCD and 54 evaluating the association between the TCD and health outcomes (Figure 1).

### Definition of the TCD (research question 1)

#### Study characteristics

Among the 99 studies, 7 were editorials,^32–38^ 8 were literature reviews^14^^,^^39–45^ and 84 were original research studies^16^^,^^24^^,^^46–126^ (Supplementary Materials I, see Table S7 in the Supporting Information online). Editorials largely described the TCD based on the authors’ experiences and understandings (eg, Zhao and Bao^45^ described foods and analyzed the characteristics of the TCD, according to their working experience in clinical nutrition). Most descriptions of the TCD (n = 84) focused on the period post the 1990s. Three reports, respectively, discussed the TCD between the early 1960s to and late 1970s,^14^ in the early 13th century,^42^ and in the period of the feudal dynasties.^44^ In the 8 literature reviews,^14^^,^^39–45^ historical and ethnographic data were utilized to described the TCD. The historical data were mostly drawn from manuscripts and chapters in books, and consisted of descriptions of foods consumed in the TCD during various periods. The ethnographic data included direct observations of the TCD among specific populations. All the original studies (n = 84) used quantitative methods to provide a definition of the TCD, including 18 cohort studies,^46–63^ 58 cross-sectional studies,^24^^,^^64–120^ 6 case–control studies,^16^^,^^121–125^ and 2 RCTs.^126^^,^^127^ Most included studies (n = 80) applied principal component analysis (PCA) or factor analysis (FA) to derive the TCD.

#### Definitions of the TCD in all studies

Among the included studies, only 35 listed food items that characterize the TCD. The food items that appeared in at least 75% of the studies were: white rice, spinach, bokchoy, and cabbage (Table 2a and see Supplementary Materials II in the Supporting Information online). All included studies (N = 99) listed the food groups that characterize the TCD. Food groups that were reported in at least 75% of the studies were rice and leafy fresh vegetables (Table 2b, and see Supplementary Materials II in the Supporting Information online). The food items and food groups also reported in at least 50% and 25% of studies are listed in Table 2a and b (see Supplementary Materials II in the Supporting Information online). Food items that appeared in at least 50% of the studies included fruits (eg, apples, pears). However, fruit was not reported as a food group characteristic of the TCD by at least 50% of studies, which might be due to the incomparability of percentages between food items and food groups. In some studies, special foods consumed by minority groups were also mentioned, such as köröngge (the top layer of cream made by boiling cow’s or sheep’s milk until a thin layer forms on the surface) in the Mongolian diet, and Dingguan (rice with mixed vegetables and meat) in the Tujia (one of the ethnic groups in China) diet (detailed explanations of special foods are shown in Supplementary Materials I, see Table S8 in the Supporting Information online).

**Table 2:** Most-cited food items and food groups reported in the included studies

a. Food items listed in at least 75%, 50%, and 25% of the studies (n = 35)			
75%	50%	25%	Minority groups’ special foods^b^
White rice, spinach, bokchoy, cabbage	Brown rice, noodles, corn, millet, maize, porridge, rape, lettuce, celery, tomato, cucumber, carrot, white melon, onion, broccoli, pepper, apple, pear, etc.^a^, pork, freshwater fish, shellfish	Flour, steamed buns, oats, wheat, buckwheat, soup thickened with flour, beef, lamb, large intestine, chicken, duck, goose, smoked meat, dried fish, eggs, pickled vegetables, preserved salty vegetables, dried vegetables, tofu, beans, almonds, sweet potato, potato, taro	Kumis, köröngge, fermented mare’s milk, fried wheat, roasted horse meat, Ci, Zong, Dinggang, Gary tofu, oil tea, rice and wheat alcohol, braised pork belly, snake

b. Food groups listed in at least 75%, 50%, and 25% of the studies (n = 99)		
75%	50%	25%
Rice, leafy fresh vegetables	Fish and seafood, pork and pork products, wheat and wheat products	Beef and beef products, corn and coarse grain, dried legumes, eggs and egg products, nonleafy fresh vegetables, fruits, legume products, pickled, salted or canned vegetables, poultry and game, starchy roots and tubers

a Including apple, pear, orange, peach, banana, grapes, cherries, jujube, apricots, cantaloupe, watermelon, grapefruit, kiwi, strawberries.

b Detailed description of these foods can be found in the Supplementary Materials I, see Table S8 in the Supporting Information online.

#### Definitions of the TCD according to two different methods of defining regions

##### Definitions of TCD according to the Qinling Mountains–Huaihe River line

As mentioned above, since only 35 studies reported food items, all subsequent analyses were conducted based on food groups, rather than individual food items. There were 4 main regions reported in the literature: all regions (n = 69, representing the TCD),^14^^,^^16^^,^^32–39^^,^^43–45^^,^^49–54^^,^^56–60^^,^^64–67^^,^^71^^,^^72^^,^^74^^,^^77–82^^,^^86^^,^^88^^,^^90^^,^^93^^,^^95^^,^^96^^,^^98^^,^^100–103^^,^^105–108^^,^^111–121^^,^^123–128^southern China (n = 21, representing the traditional southern diet),^14^^,^^24^^,^^40^^,^^46–48^^,^^63^^,^^69^^,^^70^^,^^83–85^^,^^87^^,^^89^^,^^92^^,^^94^^,^^99^^,^^104^^,^^109^^,^^110^^,^^122^ northern China (n = 14, representing the traditional northern diet),^24^^,^^40^^,^^46^^,^^55^^,^^61^^,^^62^^,^^69^^,^^73^^,^^76^^,^^91^^,^^92^^,^^94^^,^^97^^,^^109^ and minority groups (n = 3).^41^^,^^42^^,^^75^ Eight studies focused on more than one area of China.^24^^,^^40^^,^^46^^,^^69^^,^^92^^,^^94^^,^^97^^,^^109^

The studies referring to all regions (n = 69) reported similar food groups to the overall definitions identified in “Definitions of the TCD in All Studies” (Table 3a). Most of the studies (n = 52) listed rice and leafy fresh vegetables, and more than 50% of them (n = 35) reported wheat and wheat products. Pork and pork products and fish and seafoods were also reported by nearly 50% of the studies (n = 31). The studies referring exclusively to southern China (n = 21) showed some differences in food groups with at least 75% citations, compared with the descriptions of traditional northern diet. Fish and seafoods were mentioned more frequently (in 75% of the studies). They also included calorically sweetened beverages and ready-to-eat cereals and porridge, which were not mentioned in other areas (Table 3b). Studies referring exclusively to northern China (n = 14) reported food groups that differed from those in the studies of all regions. Most of these studies (75%, n = 11) mentioned wheat and wheat products, as well as wheat with a filling, which was different from the southern diet. Moreover, corn and coarse grains were mentioned (50%, n = 6), which was not the case for the southern diet. However, fish and seafoods, as well as legume products, were mentioned less frequently (Table 3c).

**Table 3:** Most-cited food groups reported in the included studies, by region (according to the Qinling Mountains–Huaihe River line)

a. Food groups listed in at least 75%, 50%, and 25% of the studies referring to all regions(n = 69)		
75%	50%	25%
Rice, leafy fresh vegetables	Wheat and wheat products	Beef and beef products, corn and coarse grain, dried legumes, fish and seafood, nonleafy fresh vegetables, fruits; pork and pork products, legume products, pickled, salted or canned vegetables, poultry and game, starchy roots and tubers

b. Food groups listed in at least 75%, 50%, and 25% of the studies referring to southern China(n = 21)		
75%	50%	25%
Fish and seafood, leafy fresh vegetables, rice	Nonleafy fresh vegetables, pork and pork products, poultry and game, legume products, dried legumes	Animal-based milk, beef and beef products, calorically sweetened beverages, eggs and egg products, fruits, instant food, oil, organ meats, pickled, salted or canned vegetables, processed meat, ready-to-eat cereals and porridge, wheat and wheat products, wheat with a filling


c. Food groups listed in at least 75%, 50%, and 25% of the studies referring to northern China(n = 14)		
75%	50%	25%
Wheat and wheat products, wheat with a filling	Corn and coarse grain, leafy fresh vegetables	Beef and beef products, dairy products, eggs and egg products, fish and seafood, nonleafy fresh vegetables, fruits, pickled, salted or canned vegetables, pork and pork products, rice, starchy roots and tubers, starchy root products and tuber products


d. Food groups listed in minority groups (Hakka: n = 1, Tujia: n = 1, Mongolian: n = 1)		
Hakka	Tujia	Mongolian
Dried legumes, fish and seafood, fruits, organ meat, rice, leafy fresh vegetables	Calorically sweetened beverages, corn and coarse grain, legume products, starchy roots and tubers, rice, leafy fresh vegetables, pork and pork products	Beef and beef products, dairy products, lamb and lamb products, oil, other livestock, pork and pork products, wheat and wheat products, wheat with a filling

China has 56 ethnic groups; each has its own unique cultural and culinary traditions.^129^ Three studies discussed the diet in minority ethnic regions (Hakka: n = 1^75^; Tujia: n = 1^41^; Mongolia: n = 1^42^). Using the Qinling Mountains–Huaihe River line to define the regions, Hakka and Tujia are mostly located in southern China, and Mongolia is clustered in northern China. Consumption of dried legumes and organ meats was reported as a more common characteristic of the Hakka diet compared with other minority groups’ diets; Mongolians seemed to consume red meat (including beef, lamb, and other livestock) and oil more frequently. Rice was reported in the Hakka and Tujia diets, which was similar to other studies referring to southern China^14^^,^^24^^,^^40^^,^^46–48^^,^^63^^,^^69^^,^^70^^,^^83–85^^,^^87^^,^^89^^,^^92^^,^^94^^,^^99^^,^^104^^,^^109^^,^^110^^,^^122^; wheat and wheat products and wheat with a filling were also reported to be characteristic of the Mongolian diet, as in other studies conducted in northern China (Table 3d).^24^^,^^40^^,^^46^^,^^55^^,^^61^^,^^62^^,^^69^^,^^73^^,^^76^^,^^91^^,^^92^^,^^94^^,^^97^^,^^109^

##### Definitions of TCD according to the 5-regions method

There were 6 regions represented after applying the 5-region classification method: all regions (n = 39,^24^^,^^32–35^^,^^37–40^^,^^43–49^^,^^53–56^^,^^59–63^^,^^65^^,^^69^^,^^85^^,^^87^^,^^92^^,^^94^^,^^101–103^^,^^109^^,^^110^^,^^126^^,^^127^^,^^130^ representing the TCD), southern China (n = 7,^36^^,^^50^^,^^51^^,^^77^^,^^111^^,^^113^^,^^120^ representing the traditional southern diet), northern China (n = 15,^42^^,^^71^^,^^73^^,^^74^^,^^76^^,^^79^^,^^91^^,^^95–97^^,^^100^^,^^105^^,^^106^^,^^108^^,^^128^ representing the traditional northern diet), eastern China (n = 35),^14^^,^^16^^,^^52^^,^^57^^,^^58^^,^^64^^,^^66^^,^^67^^,^^70^^,^^75^^,^^78^^,^^80–84^^,^^86^^,^^88–90^^,^^93^^,^^98^^,^^99^^,^^104^^,^^107^^,^^114–116^^,^^118^^,^^119^^,^^121–125^ representing the traditional eastern diet, western China (n = 1),^117^ and central China (n = 2,^41^^,^^72^ representing the traditional central diet).

The most cited food groups in the TCD were leafy fresh vegetables, pork and pork products, and rice (Table 4a), which is similar to the findings produced using the Qinling Mountains–Huaihe River line method. Studies referring to eastern China (n = 35), northern China (n = 15), and central China (n = 2) showed similarities in terms of leafy fresh vegetables. For staple foods, 75% of the studies from eastern (n = 29) and southern (n = 7) China mentioned rice to be characteristic of these diets, while those from northern (n = 10) China reported wheat and wheat products, with corn and coarse grains also being frequently mentioned in relation to central China (n = 2). Legume products were mentioned more frequently in southern China than in other regions. Only one article related to the western Chinese region was available,^117^ so it was not included in the food citation frequency statistics, but the food groups mentioned in the article were listed (Table 4c).

**Table 4:** Most-cited food groups present in the included studies, by region (according to the 5-regions method)

a. Food groups listed in at least 75%, 50%, and 25% of the studies referring to all regions (n = 39)		
75%	50%	25%
Leafy fresh vegetables, pork and pork products, rice	Fish and seafood, poultry and game, wheat and wheat products	Beef and beef products, dried legumes, nonleafy fresh vegetables, legume products, organ meats, wheat and wheat products, wheat with a filling

b. Food groups listed in at least 75%, 50%, and 25% of the studies referring to eastern China (n = 35)		
75%	50%	25%
Leafy fresh vegetables, rice	Fish and seafood, wheat and wheat products	Corn and coarse grain, dried legumes, eggs and egg products, nonleafy fresh vegetables, fruits, legume products, pickled, salted or canned vegetables, pork and pork products, starchy roots and tubers


c. Food groups listed in western China (n = 1)
Beef and beef products, calorically sweetened beverages, deep-fried wheat, starchy roots and tubers, wheat and wheat products

d. Food groups listed in at least 75%, 50%, and 25% of the studies referring to southern China (n = 7)		
75%	50%	25%
Rice	Leafy fresh vegetables, legume products	Beef and beef products, fish and seafood, fruits, pickled, salted or canned vegetables, starchy roots products and tubers products, wheat and wheat products


e. Food groups listed in at least 75%, 50%, and 25% of the studies referring to northern China (n = 15)		
75%	50%	25%
Leafy fresh vegetables, wheat and wheat products	Fish and seafood, pork and pork products, rice	Beef and beef products, corn and coarse grains, eggs and egg products, fish and seafood, nonleafy fresh vegetables, fruits, fungi and algae, legume products, oil, organ meats, pickled, salted or canned vegetables, poultry and game, starchy roots and tubers


f. Food groups listed in at least 75%, 50%, and 25% of the studies referring to central China (n = 2)		
75%	50%	25%
Corn and coarse grains, leafy fresh vegetables, legume products, rice, wheat and wheat products	Calorically sweetened beverages, dairy products, eggs and egg products, pork and pork products, ready-to-eat cereals and porridge, starchy roots and tubers	NA

#### Food groups inversely associated with the TCD

A total of 42 studies, which used PCA in their analysis to derive the TCD, reported food groups that are inversely associated with the TCD.^24^^,^^46–50^^,^^53–59^^,^^61–65^^,^^67^^,^^69^^,^^71^^,^^78–81^^,^^85–87^^,^^94^^,^^101–103^^,^^109^^,^^111^^,^^113^^,^^118^^,^^122–124^^,^^130^ These food groups (Table 5) were, therefore, considered not to be characteristic of the TCD. Wheat and wheat products were mostly reported to be negatively associated with the TCD (n = 19). Moreover, 25% of these studies also mentioned an inverse association between Western-style fried and fast foods, starchy roots and tubers, and rice, and the TCD.

**Table 5:** Most-cited food groups that were inversely associated with a traditional Chinese diet

Food groups listed with inverse associations in at least 50%, 25%, and 10% of the studies (n = 42)		
50%	25%	10%
Wheat and wheat products	Western-style fried and fast foods, starchy roots and tubers, rice	Corn and coarse grains, deep-fried wheat, dried legumes, fish and seafood, pork and pork products, poultry and game

#### Quantities of food consumption in the TCD

A total of 15 studies reported the amounts of food groups that were consumed in the TCD,^16^^,^^54^^,^^55^^,^^57^^,^^72^^,^^86^^,^^88^^,^^93^^,^^101^^,^^104^^,^^116^^,^^121^^,^^123^^,^^126^^,^^127^ while only 3 of them reported the quantities of food items.^88^^,^^104^^,^^127^ Hence, this review focused on the analysis of food groups, due to the limited number of studies reporting on the quantities of specific food items. One study assessed the percentage of energy distribution by food groups^16^; 5 reported the average quantities of foods consumed daily or weekly^54^^,^^72^^,^^93^^,^^121^^,^^126^; 8 studies reported the consumed food groups by quartiles/quintiles of intake.^55^^,^^57^^,^^86^^,^^88^^,^^101^^,^^104^^,^^116^^,^^123^ One study provided detailed accounts of food consumption, but was not considered in this section.^127^ This was due to its focus solely on describing food items presented in a recipe as an intervention, without providing any recommendations regarding the quantities of these foods typically consumed in the TCD.

The reported quantities of food groups consumed are shown in Supplementary Materials I (see Table S9 in the Supporting Information online). It was not possible to reach an agreement on the quantities of the food groups consumed in the TCD, because the studies applied different methods for assessing food groups and reported different amounts. For example, even in the studies that used the same method for assessing the quantity of fresh vegetables consumed in the TCD, the results varied from 279.1 g (standard deviation, SD 179)^54^ to 500 g (SD 131)^72^ per day. This wide variation was also observed for other food groups, such as fruits and eggs. Additionally, these studies grouped foods differently. For instance, some studies combined different kinds of meat together, referring to them as “meat,” while others separated meat into livestock and poultry.^72^ Likewise, the quantities of some food groups, such as soft drinks,^121^ and wheat and wheat products,^86^ were not comparable between studies as they were only reported in some of the studies. These differences limited our ability to obtain a consensus on the quantities of the food groups consumed in the TCD.

#### Risk-of-bias assessment

**Figure 2:**
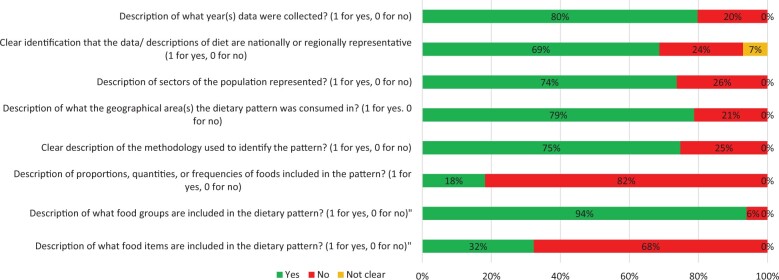
**Results of risk-of-bias assessment for review question 1**.

Most studies clearly reported 75% of the indicators for the risk-of-bias assessment (Supplementary Materials I [see Table S10 in the Supporting Information online]). However, fewer studies described the quantities of foods/food groups (n = 15) and included descriptions of food items (n = 35) (Figure 2).

### Associations between the TCD and health outcomes (research question 2)

#### Study characteristics

Among the 99 studies that defined the TCD in the current review, 54 also assessed associations between the reported TCD and NCD-related health outcomes (Supplementary Materials I [see Table S11 in the Supporting Information online]). One study was an RCT,^127^ 4 were case–control studies,^16^^,^^121–123^ 12 were cohort studies,^47^^,^^49^^,^^51–53^^,^^55–60^^,^^62^ and 37 were cross-sectional studies.^24^^,^^64–66^^,^^69^^,^^71^^,^^72^^,^^74–76^^,^^78^^,^^82–89^^,^^91^^,^^92^^,^^94^^,^^95^^,^^97–101^^,^^103^^,^^104^^,^^107^^,^^109–111^^,^^114^^,^^116^^,^^118^ All studies used a validated food frequency questionnaire to collect dietary information, and most of them (n = 52) used FA/PCA to derive the TCD. One case–control study applied latent class analysis to assess the diet, as this allowed the identification of unobserved subgroups or classes within cases.^121^ One study reported the daily consumption of foods to show the composition of the TCD.^127^

#### Cancer

Two case–control studies evaluated the association between the TCD and risk of breast cancer, with one showing null findings (OR: 0.86, 95% CI: 0.65–1.14)^121^ and the other indicating an inverse association, for both menopausal (OR: 0.68, 95% CI: 0.48–0.97) and pre-menopausal women (OR: 0.47, 95% CI: 0.29–0.76).^123^ One study also explored the traditional Cantonese diet and its association with primary liver cancer, suggesting that this dietary pattern was associated with a lower incidence risk of this cancer type (OR: 0.61, 95% CI: 0.46–0.82).^122^

#### Diabetes-related outcomes

A total of 11 studies examined the relationship between traditional diets and diabetes-related outcomes, with the majority (n = 8) finding no association.^16^^,^^64^^,^^83^^,^^84^^,^^89^^,^^91^^,^^116^^,^^118^ Type 2 diabetes was not found to be associated with the traditional diets in one case–control study and one cross-sectional study.^16^^,^^89^ Additionally, 2 cross-sectional studies focused on prediabetes, and found no evidence of association with either the TCD (OR: 1.057, 95% CI: 0.933–1.199) or the traditional southern diet (OR: 1.17, 95% CI: 0.757–1.826).^64^^,^^83^ One cross-sectional study suggested that higher adherence to the TCD was linked to lower risk of diabetes (OR: 0.810, 95% CI: 0.690–0.952).^71^ However, the other 3 studies showed no association.^47^^,^^91^^,^^116^

Four studies examined the influence of the TCD on glucose biological markers. One cohort study showed reduced concentrations of Hemoglobin A1C (–2.18%, 95% CI: –3.34% to –1.01%) and lower Homeostatic Model Assessment for Insulin Resistance indices (–10.34%, 95% CI: –16.92% to –3.24%), compared with populations consuming modern and high-wheat diets.^47^ A similar finding was also reported by another cohort study for hyperglycemia (RR: 0.59, 95% CI: 0.35–0.99).^52^ However, there were contradictory findings in 2 cross-sectional studies, suggesting no association between the traditional southern diet and glycemic control (OR: 1.08, 95% CI: 0.889–1.322),^84^ or between the TCD and insulin resistance (OR: 1.05, 95% CI: 0.62–1.76).^118^

#### Obesity-related outcomes

A total of 13 studies explored associations between the traditional diets and obesity-related outcomes; most of them (n = 8) showed that the TCD was associated with a lower risk of obesity and was inversely associated with weight gain.^49^^,^^53^^,^^57^^,^^59^^,^^72^^,^^88^^,^^101^^,^^127^ Five studies (RCT: n = 1; cross-sectional: n = 1; cohort: n = 3) reported differences in average body mass index/weight, with all of them showing that populations with highest adherence to the TCD had lower average body mass index/weight than those with the lowest adherence.^49^^,^^57^^,^^59^^,^^101^^,^^127^

Nine studies (cohort: n = 1; cross-sectional: n = 8) examined the association between traditional diets and central/general obesity, with 4 (cohort: n = 1; cross-sectional: n = 3) reporting reduced odds for obesity among those who had higher adherence to traditional diets.^53^^,^^72^^,^^88^^,^^101^ One cross-sectional study conducted analyses according to gender, indicating that the TCD was correlated with a higher risk of obesity in males (OR: 1.954, 95% CI: 1.258–3.036) but not in females (OR: 1.114, 95% CI: 0.759–1.636).^98^ Furthermore, 3 cross-sectional studies reported associations by region, suggesting that people following the traditional northern diet had a higher risk of obesity compared with people following the traditional southern diet.^24^^,^^92^^,^^109^ For example, in Zhang et al’s study, people in the highest quintiles of adherence to the traditional northern diet had greater odds of general obesity (OR: 2.28; 95% CI: 1.38–3.74) compared with people following the traditional southern diet (OR: 0.48, 95% CI: 0.29–0.78).^109^

#### Kidney disease

Two cross-sectional studies explored association between the traditional southern diet and chronic kidney disease (CKD),^87^^,^^104^ and 3 studies (cohort: n = 1; cross-sectional: n = 2) explored the relationship between TCD and kidney-related dysfunction.^51^^,^^66^^,^^85^ However, the findings were inconsistent. Shi et al^87^ indicated a positive association between the traditional southern diet and increased CKD risk (OR: 4.56, 95% CI: 3.18–6.56), while Xu et al^104^ study found no association between them (OR: 0.94, 95% CI: 0.78–1.11). The cross-sectional study conducted by He et al^66^ reported that populations with a higher adherence to the TCD had a lower risk of hyperuricemia (OR: 0.82, 95% CI: 0.426–0.922), while Shi^85^ showed a higher risk of hyperuricemia (OR: 3.24, 95% CI: 2.61–4.01) for this population group.

#### Hypertension

Eight cross-sectional studies reported on associations between the traditional diets and blood pressure, with varying findings.^75^^,^^82^^,^^94^^,^^95^^,^^97^^,^^99^^,^^103^^,^^114^ Three of them showed no association between the traditional diets and hypertension,^97^^,^^99^^,^^114^ but two studies suggested that, compared with populations with the lowest adherence to the TCD, those with the highest adherence had a higher prevalence of hypertension (PR: 1.47, 95% CI: 1.18–1.82).^82^^,^^95^ Conversely, Xu et al^103^ found that adults with higher adherence to the TCD had a lower risk of hypertension (RR: 0.69, 95% CI: 0.50–0.95). One study, which explored both the traditional northern diet and the traditional southern diet, reported that higher adherence to the traditional northern diet was associated with a higher risk of hypertension (OR: 1.30, 95% CI: 1.11–1.53), while higher adherence to the traditional southern diet was related to a lower risk of hypertension (OR: 0.73, 95% CI: 0.59–0.89).^94^

#### Cardiovascular-related outcomes

Four studies assessed the association between traditional diets and CVD incidence (cohort: n = 2; cross-sectional: n = 2)^69^^,^^71^^,^^79^^,^^83^; 3 of them reported that adults with higher adherence to traditional diets could have a lower risk of CVD.^55^^,^^56^^,^^75^ However, 1 cross-sectional study explored the association between the traditional Tianjin diet and risk of CVD by gender, showing no association in either males (OR: 1.07, 95% CI: 0.73–1.56) or females (OR: 0.90, 95% CI: 0.61–1.33).^71^

#### Lipid-related outcomes

Five studies (cohort: n = 1; cross-sectional: n = 4) explored the association between traditional diets and lipid indices^53^^,^^65^^,^^78^^,^^99^^,^^110^; two of these (both cross-sectional) reported the findings by gender.^65^^,^^78^ Males seemed to have a higher risk of developing high total cholesterol (TC) and triglyceride (TG) concentrations than females (OR: 1.10, 95% CI: 1–1.21 and OR: 1.21, 95% CI: 1.10–1.33 vs OR: 0.88, 95% CI: 0.75–1.03 and 0.96, 95% CI: 0.79–1.16, respectively).^65^ This finding was supported by the research of Lyu et al,^78^ who showed that males had a higher ratio of TGs (OR: 0.77, 95% CI: 0.62–0.95), compared with females (OR: 0.86, 95% CI: 0.69–1.07). However, no association was found between the traditional Jiangsu diet and high TC or TG risk (OR: 1.001, 95% CI: 0.834–1.201 and 0.940, 95% CI: 0.779–1.133, respectively).^99^ Zhang et al^110^ reported an inverse association between the traditional southern diet and high-density lipoprotein cholesterol in females (beta coefficient β: –1.86, 95% CI: –3.39 to –0.33).

#### Other NCD-related outcomes

A total of 8 studies explored the association between traditional diets and other NCD-related outcomes (cohort: n = 3; cross-sectional: n = 5).^58^^,^^60^^,^^62^^,^^69^^,^^74^^,^^86^^,^^100^^,^^107^ One cross-sectional study reported that the traditional northern diet was associated with an elevated risk of stroke, compared with the traditional southern diet (OR: 1.82, 95% CI: 1.6–2.43).^69^ The population with higher adherence to the TCD also showed a higher risk of anemia and asthma.^58^^,^^86^ However, higher adherence to the TCD was reported to be inversely associated with metabolic syndrome risk (OR: 0.72, 95% CI: 0.596–0.952).^100^ Three studies (cohort: n = 1; cross-sectional: n = 2) examined the relationship between TCD and the risk of chronic obstructive pulmonary disease, endoscopic gastric mucosal atrophy, and non-alcohol fatty liver, respectively; no evidence of an association was found for any of these outcomes.^62^^,^^74^^,^^107^

#### Assessment of the risk of bias and quality of reporting

The assessment of risk of bias, separately for study design, is shown in Supplementary Materials I (see Table S12 in the Supporting Information online). Case–control studies were assessed as having high risk of bias in the definition of cases and the response rates for cases and controls. For cohort studies, even though half of them were assessed as having an overall low risk of bias, a potential risk in inadequate follow-up of cohorts was identified. The cross-sectional studies showed a potentially high risk of bias in the sample size, as most did not provide justification for this construct. The RCT was assessed as having a high risk of bias due to the missing outcome data and information on measurement of outcomes, as well as the lack of justification provided for deviations from intended interventions. Overall, no cohort studies, 25% of case–control studies (n = 1 of 4), 5% of cross-sectional studies (n = 2 of 37), and 100% of RCTs (n = 1 of 1) were assessed as having a high risk of bias.

Regarding the quality of reporting, no study reported all items required by the either STROBE statement or the CONSORT statement (Supplementary Materials I, see Table S13 and Figure S1 in the Supporting Information online). In relation to the STROBE statement, few studies justified the sample size or the analyses of subgroups/interactions. Only one study was assessed by using the CONSORT statement; in that study’s report, information was not reported on trial design, participants, outcomes, sample size, randomization, allocation concealment mechanism, blinding, statistical methods, recruitment, numbers analyzed, ancillary analyses, and harms.

## DISCUSSION

To our knowledge, this is the first study to systematically review the evidence in the existing literature to obtain a definition of the TCD, and to assess any associations between this dietary pattern, as described in the literature, and NCD outcomes. This systematic review revealed that rice and leafy fresh vegetables were the most-cited food groups constituting the overall TCD, and white rice, spinach, bokchoy, and cabbage were the most frequently cited food items characteristic of the TCD. Subgroup analyses indicated that food groups varied among regions. The traditional diets of minority groups were found to resemble the traditional diets of the Han population in areas where these groups coexisted. This finding diverged from that of a prior cohort study, which suggested a difference between the diets of minority groups and the Han majority.^129^ This disparity might be attributed to the limited number of included studies focusing on minority groups (n = 3) in this review. Investigations into the relationship between the TCD and health outcomes suggested that adherence to the traditional diet could lower the risk of obesity and prevent weight gain. Associations with other health outcomes remain unclear, however, which might be due to inconsistencies in the definition of the TCD used across studies.

### Definition of the TCD

Rice and leafy fresh vegetables were the food groups with at least 75% citation frequency across studies. This finding is similar to that of a previous narrative review, which identified leafy vegetables, rice, and wheat as fundamental elements of the TCD.^39^ Wheat, corn and coarse grains have also previously been reported as main features of the TCD, suggesting their importance should be equal to that of rice,^34^^,^^35^ but in this review they were reported in fewer studies than rice. The regional traditions and the availability of wheat in northern China and rice in the south might be the main drivers of these differences across studies.^46^ As most of the studies included in the current systematic review were conducted in the south, this could have resulted in the studies referring to all regions showing a higher frequency of rice consumption than of wheat and wheat products.

The subgroup analyses revealed differences in the frequency of food groups between regions. For example, corn and coarse grains were reported for more studies in northern, compared with southern, China, according to the Qinling Mountains–Huaihe River line. This might be attributed to the higher production of corn and coarse grains in northern regions, which potentially influences food availability.^131^ Since the early 1980s, there has been a northward shift in the production of corn and coarse grains.^131^ Households in the northern regions have relied heavily on locally produced foods for essential nutrition,^132^ which potentially influenced the consumption of food groups in these regions. However, one study suggested that the majority of corn and coarse products were consumed in southern China, despite their production in northern China, which might be attributed to the development of inter-regional trade and economic disparities.^133^ Further studies are needed in order to clarify the varying frequencies of food group consumption across Chinese regions.

Studies referring to the north and east of China (according to the five-region classification system) found that people there were more likely to consume organ meats (liver and blood), and seeds and nuts (peanuts, pumpkin seeds, pistachios, almonds, walnuts, cashew nuts, sesame seeds). It is possible that these food groups are consumed to different extents in the different regions of the country due to cultural differences in eating habits. For example, gathering seeds and nuts has been an important custom contributing to plant-based sustenance in northern regions since the Yangshao period (around 5000–3000 BCE).^134^ Additionally, the consumption of organ meat has a long history in the east of China, dating back to the Zhou dynasty (1046–256 BCE).^44^ In traditional Chinese medicine, organ meat is believed to have healing properties and is considered to be a nourishing food that can strengthen the body. Organ meat has also been associated with the concept of “yang” or warmth, which is considered beneficial for health in traditional Chinese culture.^135^^,^^136^

Some food groups, such as calorically sweetened beverages (eg, bubble tea), cakes (eg, soy pudding), animal-based milk products (milk, yogurt), instant foods (eg, instant noodles), organ meats, nuts and seeds were only mentioned in some studies, and with a relatively low frequency. It has been reported that alcoholic beverages, calorically sweetened beverages, cakes and animal-based dairy products, as well as instant foods, are more characteristic of a Western diet.^137^ This suggests that increased consumption of certain food groups has made the TCD become more similar to the Western diet over time.^46^ Additionally, considering that most of the included studies (n = 80) used a posteriori methods (such as PCA) to derive dietary patterns, the foods included in the TCD essentially reflected the current diets of the population but did not necessarily reflect the accurate traditional diet, even though they were defined as characteristic of the TCD by the included studies.

Wheat and wheat products (dumplings, cassava) were mostly reported as being inversely associated with the TCD (n = 19). However, at least 50% of studies in eastern and northern China reported that wheat was characteristic of the TCD. The overall inverse association was probably related to the unbalanced number of studies conducted in the north and the south that were included in this review. The traditional southern diet was recognized as having a high intake of rice and being inversely associated with wheat. In contrast, the traditional northern diet was usually characterized by high consumption of wheat and wheat products, as well as starchy roots and tubers, such as potatoes.^69^ As most of the included studies represented southern China, the inverse association between the TCD and wheat and starchy roots and tubers could be explained by this.

The consumption of rice, wheat, vegetables, and poultry has been a significant part of the TCD since the Qin Dynasty, dating back approximately 2230 years. This is often described as “5-grain for nourishment, 5 animals for the benefit,”^138^ and can be attributed to longstanding traditions of rice, grain, and cereal cultivation, coupled with poultry breeding, in China.^139^ During the Northern Song Dynasty, there was a notable shift toward lamb as the primary meat source, due to the thriving trade between the northern and southern regions of China, resulting in the transportation of significant quantities of lamb from the northern grasslands to the south.^44^ However, in more recent times, pork has emerged as the predominant food group within the TCD. This prominence of pork might have been influenced by evolving social customs and the active promotion of the pig industry.^32^^,^^40^^,^^46^ The consumption of soy products has also increased and has been a component of the TCD since the Song Dynasty. This is due to the recognition of soy’s edible value and the development of various techniques (such as fermentation) for preparing soy products.^140^ Furthermore, post-2000 research indicates that processed meats and soft drinks might be part of the TCD,^83^^,^^84^ but were absent in earlier TCD studies.^32^^,^^40^ This transition could be attributable to economic shifts and the adoption of contemporary research methodologies in more recent studies.^46^^,^^141^ In addition to the changes in food groups, cooking methods have also exhibited change over time. Before the 1990s, preservation and drying were popular as main cooking methods,^14^^,^^42^^,^^44^ but recent studies indicate that their popularity has declined. This trend may be linked to an increasing public awareness of health concerns, which has prompted investigation into the health impacts of these cooking methods.^142^ Therefore, the TCD has been influenced by environmental, cultural, and economic factors.^143^ Further research is essential in order to delineate the changes in the TCD components over time.

When trying to develop a consistent definition of the TCD for future research, some of the food groups identified should be considered cautiously. It might be possible to include nuts, seeds, and organ meat in the definition of the TCD, as these foods are still an important part of the diet in contemporary times, and they were mentioned by the included studies from several regions. However, beverages (such as soft drinks and juice), cakes, animal-based dairy products, and instant foods might not be as widely accepted as part of the TCD, as they might not have been part of the TCD in the past.

### Differences in regional classification methods leading to TCD definition disparities

The reported frequencies of food groups consumed differed according to the method of regional classification. For example, the 5-regions classification method generated an eastern region, in which food groups that were characteristic of the diet were similar to those of the southern region as identified via the Qinling Mountains–Huaihe River line classification. Another example is that, compared with the food groups consumed within the original northern region, the northern area generated by the five-regions method featured more studies citing leafy fresh vegetables, fish, and rice as main food groups.

There might be a number of reasons for these observed differences. It might be that, contingent upon the classification method used, the provinces contained in each region ultimately led to different numbers of studies being included in the analysis. According to the Qinling Mountains–Huaihe River line, 3 traditional dietary patterns were defined (southern, northern, and overall TCD). However, considering the variation in dietary preferences and geography within the southern and northern regions,^22^ the 5-regions method was deemed important in order to reclassify the included studies into 5 traditional dietary patterns (southern, northern, eastern, western, and central), along with the overall TCD. Both the south and north regions were identified under the two methods, but the Chinese provinces encompassed using the two classification methods differed in some areas. For example, Jiangsu and Zhejiang were categorized under eastern China, using the five-regions classification method, whereas they were considered to be part of southern China when the Qinling Mountains–Huaihe River line method was used. These geographical discrepancies in the TCD, stemming from the different regional classification methods, could explain the variation in the consumption of food groups, as reported by the reviewed studies. Future studies assessing traditional diets in China should aim to develop a consensus definition of how regions (and their provinces) are classified, in order to take into account regional traditions and geographical characteristics.

### Specific food items included in the TCD

China has 56 ethnic groups, with the Han Chinese accounting for over 90% of the total population. The remaining population, approximately 10%, is distributed among the other 55 ethnic groups, which are commonly called the 55 minority groups.^144^ It should be acknowledged that each minority group might consume its own foods and use unique cooking methods, due to its culture or heritage.^131^^,^^145^ Two of the studies included in this review reported both food items and food groups consumed by minority groups,^41^^,^^42^ and 1 reported only food groups.^75^ For example, Mongolian people consume horse milk and its related products, as well as köröngge (boiled milk skin), which was not commonly consumed by other ethnic groups.^42^ The Tujia population were reported to consume Ci (made from glutinous rice, either steamed or deep-fried) and Dinggang (a mixed dish of rice, vegetables, and small amounts of preserved meat) as staple foods.^41^

There are more than 1100 types of foods in China, including more than 300 types of vegetables, which makes it challenging to list all of them in one study.^22^ Some food items not commonly consumed in China, such as dogs and donkeys, were also reported.^40^ Despite miso also being reported in one study, it is commonly recognized that this food is characteristic of the traditional Japanese diet.^146^ When defining the TCD, it would be important to consider whether foods that are less commonly consumed, and foods that are more characteristic of other traditional dietary patterns, should be identified as part of the TCD. Future studies should assess the feasibility of integrating these less frequently consumed food items into a TCD, especially if such a dietary pattern should be inclusive of all the minority groups residing in China.

### Quantities of food groups included in the TCD

When defining a dietary pattern, it is essential to report the quantities of the various food groups consumed, especially when exploring the association between diet and health outcomes. This is because different food quantities might result in completely different outcomes.^18^^,^^147^ The current review revealed that only a few studies (n = 15) reported food group quantities, with heterogeneous cross-study reporting of the quantities of food groups being consumed in the TCD. This heterogeneity means it is challenging to identify the exact quantities of the various food groups in the TCD. For example, one study used the percentages of total energy intake to determine the quantities of food groups consumed,^16^ while others used the average quantities of food groups consumed per week or per day.^54^^,^^72^^,^^93^^,^^121^^,^^126^ Comparing the results based on these different methods would be challenging.^141^ Additionally, even if the same method was applied when investigating the quantities of food groups consumed in the TCD, the various standards for categorizing food groups would also possibly result in different outcomes. For example, leafy and nonleafy vegetables might be grouped into 1 food group (“vegetables”) or 2 different food groups, which might cause further complexity in how the TCD is defined. Therefore, further explorations are essential for defining the number of food groups that characterize the TCD, and the quantities that were consumed in the traditional pattern.

### Association between the TCD and health outcomes

This review also explored the association between traditional diets and health outcomes. Considering that approximately 80% of the included studies were observational in nature, and that the definition of the TCD was based on a posteriori analyses and data-driven methods, the derived TCD might reflect current dietary habits in China, rather than what can actually be considered “traditional.”^148^ Additionally, the various definitions of the TCD reported in these studies (such as the overall TCD, the traditional southern diet and the traditional northern diet) limited our ability to compare the results of studies assessing the same NCD outcomes. Hence, it was not deemed possible to reach robust evidence in this review regarding associations between the TCD and health outcomes. Nevertheless, the current evidence suggests that associations with hypertension, cancer, CVD, kidney disease, and other NCD-related outcomes are inconsistent. The studies showed both positive and negative, as well as null, associations. This might also be due to the differences in the characteristics of the populations and regions assessed across the studies. For example, a study focusing on Inner Mongolian adults showed that there was no association between the TCD and hypertension,^97^ while a study exploring older adults across the whole country reported an inverse relationship with hypertension.^103^ Therefore, these findings should be carefully interpreted, particularly because the number of studies measuring the same outcomes was often small.

The majority of the studies (n = 10 of 13) reported an inverse association between adherence to the TCD and obesity-related outcomes. As identified in this review, rice, fish and seafood, and leafy-fresh vegetables are the main components of the TCD, similar to the Mediterranean diet, which has been shown to have a protective benefit against weight gain.^149^ Nonetheless, the relationship between obesity and rice intake is still being disputed. A cross-sectional study conducted in Zhejiang Province found an inverse association between rice consumption and overweight.^88^ However, a cross-sectional study in Korea showed that a higher intake of white rice was positively associated with obesity among Korean adults.^150^ No association was reported between rice and obesity among Iranian adults in yet another study.^151^ It could be that gender influences the association between rice and obesity. Rice, when used as the staple food, has been cross-sectionally associated with greater risk of obesity in male but not female middle-aged and older adults in Shangai.^152^ These discrepancies might be attributable to ethnic differences, rice varieties, other food groups consumed alongside rice, and cooking methods.^152^ For example, a previous systematic review that included 4 cohort studies reported that white rice was heavily related to an increased risk of type 2 diabetes in Asian and Japanese populations.^68^ Therefore, more studies are needed in order to evaluate the influence of the food groups present in the TCD, and to provide robust evidence for associations of the TCD with health outcomes.

The food supply in the various study areas might also influence the associations between the TCD and health outcomes. For example, one study indicated that cadmium contamination in certain food supplies could be a contributing factor to a positive association between CKD and the traditional southern diet.^87^ Additionally, for geographical reasons, there has been a shortage of fresh vegetable supply in Inner Mongolia.^97^ The limited consumption of fresh vegetables might explain the pickled vegetable presence in the traditional diet in this area, which might contribute to the higher prevalence of hypertension in this region, compared with other regions.^153^

Nevertheless, the TCD, as defined in this review, could be considered a healthy dietary pattern, as many of its characteristics (eg, leafy fresh vegetables, rice, wheat and wheat products, poultry and seafood, in addition to specific fruits) have been considered “protective” in other diets.^154–156^ The TCD also contains legume products and coarse grains, which are recommended components of healthy diets.^157^ However, the quantities of the foods consumed in the TCD have not been determined, and quantities are essential for defining a dietary pattern. The preparation and cooking methods should also be assessed in future studies, because these aspects are regarded as an important component of traditional Chinese food culture.^112^ Different kinds of food preparation and cooking methods can affect the associations between diet and health outcomes. For example, deep-frying can produce acrylamide, which is a harmful organic chemical substance that increases the risk of several types of cancer.^158^ Therefore, the food preparation techniques and cooking methods that are most in line with dietary recommendations should potentially be prioritized and promoted as part of the TCD.

### Strengths and limitations

This is the first study that has systematically evaluated the existing literature on the TCD and its association with health outcomes, including a comprehensive gathering of related literature, with no restrictions regarding time-frame or geographical location. Other studies have explored traditional diets by interviewing elderly people (eg, in Greek villages to investigate the Mediterranean diet^159^) or by assessing the traditional and regional products still being used (eg, to determine the traditional Japanese diet^160^). However, eating habits in China have changed significantly over time, and differences have emerged in eating cultures and products between Chinese regions,^161^ so these methods were not considered appropriate for exploring the TCD. Hence, this review described the TCD according to the definitions, descriptions, and explorations reported in the existing literature. Moreover, the frequency of the consumption of food items and food groups was investigated in order to identify the consistent characteristics of the TCD. This type of method could provide a more comprehensive and less-biased definition of the TCD than that offered by personal perspectives. In addition to using the Qinling Mountain–Huaihe line to divide China into the south and north, this study used the 5-region classification method to recategorize regions when reporting traditional diets. This method could be more accurate in defining the TCD, as it considers the dietary preferences and geographical characteristics that might define a traditional dietary pattern.^22^

This review also has limitations. First, the included studies had different criteria for grouping foods, which might have led to biases. However, a standardized food classification system was used to address this limitation.^21^ For instance, some studies only used meat or red meat as a food group, while the food grouping method applied in this review categorized meat into pork, lamb, beef, poultry, and game. Although attempts were made to contact the authors of these studies to check the original data, with the aim of identifying appropriate food groups, there remained studies for which the food groups could not be accurately categorized. This was the case for vegetable classification, as some studies just cited vegetables as the food group, rather than separating them into leafy vegetables and nonleafy vegetables. Second, when using the 5-region method to recategorize the regions for subgroup analysis, there were only a few studies for some regions (one study in western China and 2 studies in central China), which may have limited the generalizability and representativeness of the food groups reported.

The majority of studies (n = 80) in this review used a posteriori approaches to define the TCD, which would have reflected current eating by participants.^148^ However, the traditional diet should take the foods/eating habits of the past into account; therefore, one could question whether the defined TCD was actually “traditional.” Future studies should utilize a clear definition of the TCD and consider employing an a priori approach when assessing the relationship between TCD and health outcomes. Additionally, nearly all studies (n = 53) that assessed associations with health outcomes were observational, and thus more prone to risk of bias. For example, none of the case–control studies reported response rates for either cases or controls, which limited the comparability between groups.

Another limitation of this review was that the impact of cooking practices was not assessed by the majority of the included studies. In addition to specific foods consumed, how people eat can be regarded as an important part of traditional diets.^162^ Specifically, food that takes a long time to prepare, food prepared “as one’s grandmother would have done,” and the use of steaming and boiling techniques have been characterized as traditional methods of food preparation and cooking.^162^^,^^163^ Instant foods and fried and grilled-style methods are commonly regarded as modern methods of cooking.^164^ However, as not all studies in this review mentioned cooking methods, with most of them only reporting on the dietary pattern, information on cooking practices could not be extracted. Future studies should combine the exploration of foods, food preparation, and cooking methods when assessing the TCD.

## CONCLUSION

This systematic review found that rice and leafy vegetables are the primary components of the TCD, with fish and seafood, pork and pork products, and wheat and wheat products also playing important roles in the TCD. Due to China’s vast territory and considerable regional differences, the TCD was found to vary from region to region. Wheat and wheat products, wheat with a filling, and corn and coarse grains were the most frequently consumed food groups in the north, while the diet in the south commonly involved rice, fish and seafood, leafy fresh vegetables, and pork and pork products. More studies are essential, to assess the consumption of food groups and specific food items, as well as the typical quantities consumed in the TCD. Given the heterogeneity among the included studies, no consensus could be reached on associations between the TCD and health outcomes. However, the available evidence indicates that the TCD is inversely associated with risk of obesity and weight gain, indicating its potential health benefits. Nevertheless, the findings of this review should be interpreted cautiously, because the TCD definitions, and the regions and populations, varied considerably in the studies reviewed. Future studies should further explore the TCD by using comprehensive methods and evaluating the quantities and cooking methods of the food groups consumed, which would be important for illustrating the health properties of this dietary pattern and its potential role in preventing NCDs in China.

## Supplementary Material

nuae013_Supplementary_Data

## Supporting Information

The following Supporting Information is available through the online version of this article at the publisher’s website.

**Supplementary materials I**: *Table S1* **PRISMA 2020 checklist** *Table S2* **Search strategy** *Table S3* **Data extraction form** *Table S4* **Food group classification system** *Table S5* **Adapted Newcastle–Ottawa Scale for observational studies** *Table S6* **List of excluded studies** *Table S7* **Characteristics of all included studies** *Table S8* **Explanations for specific foods** *Table S9* **Amounts of food groups** *Table S10* **Risk of bias for review question one** *Table S11* **Characteristics of the included studies for review question 2** *Table S12* **Risk of bias for review question 2** *Table S13* **Quality reporting** *Figure S1* **Proportion of reported items in observational studies (n = 53), based on the STROBE 2007 checklist**

**Supplementary materials II**: *Sheet S1*: Frequency of the citation of food items among all included studies; *Sheet S2*: Frequency of the citation of food groups among all included studies; *Sheet S3*: Frequency of the citation of food groups for whole country (TCD) according to method 1; *Sheet S4*: Frequency of the citation of food groups for the traditional southern diet according to method 1, *Sheet S5*. Frequency of the citation of food groups for the traditional northern diet according to method 1; *Sheet S6*: Frequency of the citation of food groups for the traditional minority diet according to method 1; *Sheet S7*: Frequency of the citation of food groups for the whole country (TCD) according to method 2; *Sheet S8*: Frequency of the citation of food groups for the traditional eastern diet according to method 2; *Sheet S9*: Frequency of the citation of food groups for the traditional western diet according to method 2; *Sheet S10*: Frequency of the citation of food groups for the traditional northern diet according to method 2; *Sheet S11*: Frequency of the citation of food groups for the traditional southern diet according to method 2; *Sheet S12*: Frequency of the citation of food groups for the traditional central diet according to method 2; *Sheet S13*: Frequency of food groups reported as inversely associated with TCD
