# Supplementary material for: Exploring the traditional Chinese diet and its association with health status—a systematic review
Source: Nutr Rev. 2024 Aug 2;83(2):e237–56. doi: 10.1093/nutrit/nuae013 (PMC11723156; doi:10.1093/nutrit/nuae013)
Supplement: nuae013_Supplementary_Data [file nuae013_supplementary_data.zip › nuae013_Supplementary_Data/Supplementary materials II.docx]

**Supplementary Materials II**

**Table S1. Frequency of the citation of food items anmong all included studies**

**Table S2. Frequency of the citation of food groups anmong all included studies**

**Table S3. Frequency of the citation of food groups for whole country (TCD) according to the Qinling Mountain Huaihe line classification method**

**Table S4. Frequency of the citation of food groups for the traditional southern diet according to the Qinling Mountain Huaihe line classification method**

**Table S5. Frequency of the citation of food groups for the traditional northern diet according to the Qinling Mountain Huaihe line classification method**

**Table S6. Frequency of the citation of food groups for the traditional minority diet according to the Qinling Mountain Huaihe line classification method**

**Table S7. Frequency of the citation of food groups for the whole country (TCD) according to the five-regions classification method**

**Table S8. Frequency of the citation of food groups for the traditional eastern diet according to the five-regions classification**

**Table S9. Frequency of the citation of food groups for the traditional western diet according to the five-regions classification**

**Table S10. Frequency of the citation of food groups for the traditional northern diet according to the five-regions classification**

**Table S11. Frequency of the citation of food groups for the traditional southern diet according to the five-regions classification**

**Table S12. Frequency of the citation of food groups for the traditional central diet according to the five-regions classification**

**Table S13. Frequency of the citation of food groups reported as inversely associated with TCD**
