# Supplementary material for: Exploring the traditional Chinese diet and its association with health status—a systematic review
Source: Nutr Rev. 2024 Aug 2;83(2):e237–56. doi: 10.1093/nutrit/nuae013 (PMC11723156; doi:10.1093/nutrit/nuae013)
Supplement: nuae013_Supplementary_Data [file nuae013_supplementary_data.zip › nuae013_Supplementary_Data/Supplementary materials I_.docx]

**Supplementary materials I:**

**Table S1. PRISMA 2020 Checklist**

| **Section and Topic** | **Item #** | **Checklist item** | **Location where item is reported** |
| --- | --- | --- | --- |
| **TITLE** | | |  |
| Title | 1 | Identify the report as a systematic review. | 1 |
| **ABSTRACT** | | |  |
| Abstract | 2 | See the PRISMA 2020 for Abstracts checklist. | 1-2 |
| **INTRODUCTION** | | |  |
| Rationale | 3 | Describe the rationale for the review in the context of existing knowledge. | 3-4 |
| Objectives | 4 | Provide an explicit statement of the objective(s) or question(s) the review addresses. | 4 |
| **METHODS** | | |  |
| Eligibility criteria | 5 | Specify the inclusion and exclusion criteria for the review and how studies were grouped for the syntheses. | 5, 47 |
| Information sources | 6 | Specify all databases, registers, websites, organisations, reference lists and other sources searched or consulted to identify studies. Specify the date when each source was last searched or consulted. | 5 |
| Search strategy | 7 | Present the full search strategies for all databases, registers and websites, including any filters and limits used. | Supplementary materials I |
| Selection process | 8 | Specify the methods used to decide whether a study met the inclusion criteria of the review, including how many reviewers screened each record and each report retrieved, whether they worked independently, and if applicable, details of automation tools used in the process. | 6 |
| Data collection process | 9 | Specify the methods used to collect data from reports, including how many reviewers collected data from each report, whether they worked independently, any processes for obtaining or confirming data from study investigators, and if applicable, details of automation tools used in the process. | 6 |
| Data items | 10a | List and define all outcomes for which data were sought. Specify whether all results that were compatible with each outcome domain in each study were sought (e.g. for all measures, time points, analyses), and if not, the methods used to decide which results to collect. | 7-9 |
|  | 10b | List and define all other variables for which data were sought (e.g. participant and intervention characteristics, funding sources). Describe any assumptions made about any missing or unclear information. | 7-9 |
| Study risk of bias assessment | 11 | Specify the methods used to assess risk of bias in the included studies, including details of the tool(s) used, how many reviewers assessed each study and whether they worked independently, and if applicable, details of automation tools used in the process. | 9-10 |
| Effect measures | 12 | Specify for each outcome the effect measure(s) (e.g. risk ratio, mean difference) used in the synthesis or presentation of results. | 6, Supplementary materials I |
| Synthesis methods | 13a | Describe the processes used to decide which studies were eligible for each synthesis (e.g. tabulating the study intervention characteristics and comparing against the planned groups for each synthesis (item #5)). | 6-9 |
|  | 13b | Describe any methods required to prepare the data for presentation or synthesis, such as handling of missing summary statistics, or data conversions. | 7-9 |
|  | 13c | Describe any methods used to tabulate or visually display results of individual studies and syntheses. | 7-9 |
|  | 13d | Describe any methods used to synthesize results and provide a rationale for the choice(s). If meta-analysis was performed, describe the model(s), method(s) to identify the presence and extent of statistical heterogeneity, and software package(s) used. | 7-9 |
|  | 13e | Describe any methods used to explore possible causes of heterogeneity among study results (e.g. subgroup analysis, meta-regression). | 7-9 |
|  | 13f | Describe any sensitivity analyses conducted to assess robustness of the synthesized results. | Not applicable |
| Reporting bias assessment | 14 | Describe any methods used to assess risk of bias due to missing results in a synthesis (arising from reporting biases). | 10 |
| Certainty assessment | 15 | Describe any methods used to assess certainty (or confidence) in the body of evidence for an outcome. | Not applicable |
| **RESULTS** | | |  |
| Study selection | 16a | Describe the results of the search and selection process, from the number of records identified in the search to the number of studies included in the review, ideally using a flow diagram. | 10, Figure 1 |
|  | 16b | Cite studies that might appear to meet the inclusion criteria, but which were excluded, and explain why they were excluded. | 10-11, Supplementary material I |
| Study characteristics | 17 | Cite each included study and present its characteristics. | 10-11, Supplementary material I |
| Risk of bias in studies | 18 | Present assessments of risk of bias for each included study. | 15, 21, Supplementary material I |
| Results of individual studies | 19 | For all outcomes, present, for each study: (a) summary statistics for each group (where appropriate) and (b) an effect estimate and its precision (e.g. confidence/credible interval), ideally using structured tables or plots. | 11-21 |
| Results of syntheses | 20a | For each synthesis, briefly summarise the characteristics and risk of bias among contributing studies. | 11-21 |
|  | 20b | Present results of all statistical syntheses conducted. If meta-analysis was done, present for each the summary estimate and its precision (e.g. confidence/credible interval) and measures of statistical heterogeneity. If comparing groups, describe the direction of the effect. | 11-21, Supplementary material I |
|  | 20c | Present results of all investigations of possible causes of heterogeneity among study results. | 11-21 |
|  | 20d | Present results of all sensitivity analyses conducted to assess the robustness of the synthesized results. | Not applicable |
| Reporting biases | 21 | Present assessments of risk of bias due to missing results (arising from reporting biases) for each synthesis assessed. | 21 |
| Certainty of evidence | 22 | Present assessments of certainty (or confidence) in the body of evidence for each outcome assessed. | Not applicable |
| **DISCUSSION** | | |  |
| Discussion | 23a | Provide a general interpretation of the results in the context of other evidence. | 21-30 |
|  | 23b | Discuss any limitations of the evidence included in the review. | 31-32 |
|  | 23c | Discuss any limitations of the review processes used. | 31-32 |
|  | 23d | Discuss implications of the results for practice, policy, and future research. | 22-30, 32 |
| **OTHER INFORMATION** | | |  |
| Registration and protocol | 24a | Provide registration information for the review, including register name and registration number, or state that the review was not registered. | 4 |
|  | 24b | Indicate where the review protocol can be accessed, or state that a protocol was not prepared. | 4 |
|  | 24c | Describe and explain any amendments to information provided at registration or in the protocol. | Not applicable |
| Support | 25 | Describe sources of financial or non-financial support for the review, and the role of the funders or sponsors in the review. | 33 |
| Competing interests | 26 | Declare any competing interests of review authors. | 33 |
| Availability of data, code and other materials | 27 | Report which of the following are publicly available and where they can be found: template data collection forms; data extracted from included studies; data used for all analyses; analytic code; any other materials used in the review. | 34-50, Supplementary materials I, II |

*From:*  Page MJ, McKenzie JE, Bossuyt PM, Boutron I, Hoffmann TC, Mulrow CD, et al. The PRISMA 2020 statement: an updated guideline for reporting systematic reviews. BMJ 2021;372:n71. doi: 10.1136/bmj.n71

**Supplementary materials I:**

**Table S2. Search strategy**

- **Anthropology Plus**

Date: 25^th^ April 2022

Search strategy used:

TX (Chinese OR (Chinese people) OR China)

AND

TX (traditional OR native OR regional OR indigenous)

AND

TX (diet* OR diet* pattern* OR eating pattern* OR food pattern* OR eating* OR eating habit* OR food habit* OR diet* habit* OR cuisine)

Expanders - Apply related words; Apply equivalent subjects

Search modes - Boolean/Phrase

***Total articles retrieved: 16***

- **PubMed**

Date: 25^th^ April 2022

Search strategy used:

((("Diet"[Mesh]) OR (diet*[Title/Abstract] OR diet* pattern*[Title/Abstract] OR eating pattern*[Title/Abstract] OR food pattern*[Title/Abstract] OR eating*[Title/Abstract] OR eating habit*[Title/Abstract] OR food habit*[Title/Abstract] OR diet* habit*[Title/Abstract] OR cuisine [Title/Abstract]))

AND

(traditional [Title/Abstract] OR native[Title/Abstract] OR regional[Title/Abstract] OR indigenous[Title/Abstract]))

AND

(Chinese [Title/Abstract] OR (Chinese people[Title/Abstract]) OR China[Title/Abstract])

***Total articles retrieved: 2353***

- **CINAHL**

Date: 25^th^ April 2022

Search strategy used:

(TI (Chinese OR (Chinese people) OR China) OR AB Chinese OR (Chinese people) OR China))

AND

(TI (traditional OR native OR regional OR indigenous) OR AB (traditional OR native OR regional OR indigenous))

AND

(TI (diet* OR diet* pattern* OR eating pattern* OR food pattern* OR eating* OR eating habit* OR food habit* OR diet* habit* OR cuisine) OR AB (diet* OR diet* pattern* OR eating pattern* OR food pattern* OR eating* OR eating habit* OR food habit* OR diet* habit* OR cuisine))

Expanders - Apply related words; Apply equivalent subjects

Search modes - Boolean/Phrase

***Total articles retrieved: 1392***

- **Web of Science Core Collection**

Date: 25^th^ April 2022

Search strategy used:

(TI=(Chinese or Chinese people or China)) OR AB=(Chinese or Chinese people or China)

AND

(TI=(traditional or native or regional or indigenous)) OR AB=(traditional or native or regional or indigenous)

AND

(TI=(diet* or diet* pattern* or eating pattern* or food pattern* or eating* or eating habit* or food habit* or diet* habit* or cuisine)) OR AB=(diet* or diet* pattern* or eating pattern* or food pattern* or eating* or eating habit* or food habit* or diet* habit* or cuisine)

***Total articles retrieved: 2986***

- **Medline on OvidSP**

Date: 25^th^ April 2022

Search strategy used:

(Chinese or Chinese people or China).mp. [mp=title, abstract, original title, name of substance word, subject heading word, floating sub-heading word, keyword heading word, organism supplementary concept word, protocol supplementary concept word, rare disease supplementary concept word, unique identifier, synonyms]

AND

(traditional or native or regional or indigenous).mp. [mp=title, abstract, original title, name of substance word, subject heading word, floating sub-heading word, keyword heading word, organism supplementary concept word, protocol supplementary concept word, rare disease supplementary concept word, unique identifier, synonyms]

AND

(diet* or diet* pattern* or eating pattern* or food pattern* or eating* or eating habit* or food habit* or diet* habit* or cuisine).mp. [mp=title, abstract, original title, name of substance word, subject heading word, floating sub-heading word, keyword heading word, organism supplementary concept word, protocol supplementary concept word, rare disease supplementary concept word, unique identifier, synonyms]

***Total articles retrieved: 2876***

- **Embase**

Date: 25^th^ April 2022

Search strategy used:

(Chinese or Chinese people or China).mp. [mp=title, abstract, heading word, drug trade name, original title, device manufacturer, drug manufacturer, device trade name, keyword heading word, floating subheading word, candidate term word]

AND

(traditional or native or regional or indigenous).mp. [mp=title, abstract, heading word, drug trade name, original title, device manufacturer, drug manufacturer, device trade name, keyword heading word, floating subheading word, candidate term word]

AND

(diet* or diet* pattern* or eating pattern* or food pattern* or eating* or eating habit* or food habit* or diet* habit* or cuisine).mp. [mp=title, abstract, heading word, drug trade name, original title, device manufacturer, drug manufacturer, device trade name, keyword heading word, floating subheading word, candidate term word]

***Total articles retrieved: 3964***

- **ProQuest Dissertations & Theses Global**

Date: 25^th^ April 2022

Search strategy used:

(ab(Chinese OR Chinese people OR China) OR ti(Chinese OR Chinese people OR China))

AND

(ab(traditional or native or regional or indigenous) OR ti(traditional or native or regional or indigenous))

AND

(ab(diet* OR diet* pattern* OR eating pattern* OR food pattern* OR eating* OR eating habit* OR food habit* OR diet* habit* OR cuisine) OR ti(diet* OR diet* pattern* OR eating pattern* OR food pattern* OR eating* OR eating habit* OR food habit* OR diet* habit* OR cuisine))

Filters

 Full-text

 Language Included: English and Chinese

***Total articles retrieved: 164***

- **PsycINFO**

Date: 25^th^ April 2022

Search strategy used:

(Chinese or Chinese people or China).mp. [mp=title, abstract, heading word, table of contents, key concepts, original title, tests & measures, mesh word] AND

(traditional or native or regional or indigenous).mp. [mp=title, abstract, heading word, table of contents, key concepts, original title, tests & measures, mesh word]

AND

(diet* or diet* pattern* or eating pattern* or food pattern* or eating* or eating habit* or food habit* or diet* habit* or cuisine).mp. [mp=title, abstract, heading word, table of contents, key concepts, original title, tests & measures, mesh word]

***Total articles retrieved: 168***

- **Scopus**

Date: 25^th^ April 2022

Search strategy used:

TITLE-ABS-KEY (Chinese or Chinese people or China)

AND

TITLE-ABS-KEY (traditional or native or regional or indigenous)

AND

TITLE-ABS-KEY (diet* or diet* pattern* or eating pattern* or food pattern* or eating* or eating habit* or food habit* or diet* habit* or cuisine)

***Total articles retrieved: 68***

- **CENTRAL and Cochrane Reviews**

Date: 25^th^ April 2022

Search strategy used:

Title Abstract Keyword (Chinese or Chinese people or China)

AND

Title Abstract Keyword (traditional or native or regional or indigenous)

AND

Title Abstract Keyword (diet* or diet* pattern* or eating pattern* or food pattern* or eating* or eating habit* or food habit* or diet* habit* or cuisine)

***Total articles retrieved in Cochrane Reviews:15***

***Total articles retrieved in Trials: 437***

- **Wangfang Data**

Date: 25^th^ April 2022

Search strategy used:

[主题]传统饮食+中国传统饮食【或】[主题]传统南方饮食+传统北方饮食【或】[主题]传统饮食模式+中国传统饮食模式【或】[主题]传统饮食习惯+中国传统饮食习惯【或】[主题]传统饮食习俗+中国传统饮食习俗

Filter: 模糊 (fuzzy for subject)

全文(full-text)

排除地方志 (exclude DifangZhi)

***Total articles retrieved in Cochrane Reviews: 129***

- **China National Knowledge Infrastructure (CNKI)**

Date: 25^th^ April 2022

Search strategy used:

[主题]传统饮食 OR [主题]中国传统饮食 OR [主题]传统南方饮食OR [主题]传统北方饮食OR [主题]传统饮食模式OR [主题]中国传统饮食模式OR [主题]传统饮食习惯OR [主题]中国传统饮食习惯OR [主题]传统饮食习俗OR [主题]中国传统饮食习俗

***Total articles retrieved: 1337***

- **Wei Pu (VP) Website**

Date: 25^th^ April 2022

Search strategy used:

[题名或关键词] 传统饮食+中国传统饮食+传统南方饮食+传统北方饮食+传统饮食模式+中国传统饮食模式+传统饮食习惯+中国传统饮食习惯+传统饮食习俗+中国传统饮食习俗

Filter: 模糊 (fuzzy for subject)

***Total articles retrieved: 825***

- **中国生物医学文献数据库（SinoMed）**

Date: 25^th^ April 2022

Search strategy used:

传统饮食"[关键词:智能] OR "中国传统饮食"[关键词:智能] OR "传统南方饮食"[关键词:智能] OR "传统北方饮食"[关键词:智能] OR "中国传统饮食模式"[关键词:智能] OR "传统饮食模式"[关键词:智能] OR "传统饮食习惯"[关键词:智能] OR "中国传统饮食习惯"[关键词:智能] OR “传统饮食习俗"[加权:扩展] OR "中国传统饮食习俗"[关键词:智能]

***Total articles retrieved: 2***

**Supplementary materials I:**

**Table S3. Data extraction form**

| **For review question 1: TCD definition** | **For review question 2: Association between TCD and health outcomes** |
| --- | --- |
| Study ID | Study ID |
| Author name and article title | Author name and article title |
| Year of publication | Year/period represented |
| Country | Country |
| Study aim | Characteristics of population, besides the exposure/intervention and comparator/control |
| Characteristics of population including age, sex, ethnicity, education, BMI, health status | Description of TCD |
| Study design | Outcome and outcome measure |
| Year/period represented | Comparison method |
| Geographical location represented | Covariates |
| Description of TCD | Statistics results with p-value |
| Food items contained | Adjusted model |
| Food groups contained | Other related outcomes |
| Quantity of each foods/food groups |  |
| Methods of identification of diet |  |
| Diet collection method |  |
| Analysis method |  |
| Positive/negative loadings |  |

BMI, body mass index; ID, identification; TCD, traditional Chinese diet

**Supplementary materials I:**

**Table S4. Food group classification system***

| **Food group** | **Examples of food items** |
| --- | --- |
| Rice | White and brown rice |
| Wheat and wheat products | Wheat noodles, wheat flour, breads, bing (without filling in it), steamed buns, butter bread, salty bread, Migao |
| Wheat with filling | Steamed stuffed bun, bing (with filling in it), dumpling, sweet dumpling |
| Cakes, cookies and pastries | Cookies, mooncake, fruit cake, chocolate cake, fruit pie, doughnuts |
| Deep-fried wheat | Deep-fried dough stick, deep-fried cake with red bean paste and sugar, deep-fried sweet sesame seed ball, Tsampa |
| Deep-fried rice and legumes | Deep-fried rice flour doughnut, deep-fried soybean, deep-fried broad bean |
| Corn and coarse grains | Corn, corn grits, corn flour, barley, oats, foxtail millet, maize, sorghum, buckwheat |
| Grains and cereals (not specified) |  |
| Starchy roots and tubers | Potato, yam, taro, lotus root, water chestnut, cassava, sweet potato |
| Starchy root and tuber- related products | Potato starch, lotus root starch, potato flour, corn starch, starch |
| Fresh legumes | Soybean sprouts, peas with pod, mung bean sprouts |
| Dried legumes | Soybean flour, dried beans, beans flour, roasted broad bean, miscellaneous bean |
| Legume products | Tofu, tofu products, red/mung bean paste, soya products |
| Nuts and seeds | Sesame, sunflower, watermelon seeds, lotus seeds, peanuts, walnuts, almonds, hazelnuts, pine-nuts, pistachios, cashew nuts |
| Fresh vegetables, non-leafy | Cauliflower, tomatoes, cucumber, zucchini |
| Fresh vegetables, leafy | Spinach, ‘bok choy’, cabbage |
| Fungi and algae | Mushrooms, kelp |
| Pickled, salted or canned vegetables | Canned tomato sauce, preserved vegetables, vegetables in soy sauce |
| Dried vegetables | Dried radish, dried bamboo shoot, dried lily |

**Table S4 (continued)**

| **Food group** | **Examples of food items** |
| --- | --- |
| Fruits | Fresh and canned (no added sugar) fruits |
| Dried fruits | Dates, dried longan, raisins |
| Preserved fruits with added sugar | Dried and canned fruit with added sugar |
| Other livestock | Donkey, horse |
| Mini livestock | Rabbit |
| Beef and beef products | Beef, Beef Meatballs, cattle, yak beef |
| Lamb and lamb products | Lamb, lamb meatballs |
| Pork and pork products | Pork tenderloin pork, pork tendons, Pork belly, leg, rib chop |
| Organ meats | Liver, kidney, large intestine, blood |
| Processed meats | Sausages, ham, luncheon meat, dried meat, smoked meat, bacon |
| Poultry and game | Chicken, duck, goose |
| Eggs and egg products | Whole eggs, yolk, white, preserved eggs |
| Fish and seafood | Fresh- and salt-water fish, dried fish, shellfish, shrimp |
| Soy milk | Sweetened and un-sweetened soy milk |
| Animal-based milk | Cow milk, goat milk, skim milk, flavored milk |
| Dairy products | Cheese, yogurt |
| Sweetened dairy products | Ice cream |
| Western-style fried/fast-foods | Fried chicken, fried meatballs, sandwich, hamburger, hotdog, pizza |
| Instant foods | Instant noodles, instant dumplings |
| Liquids | Soup |
| Ready-to-eat cereals/porridge | Instant multigrain porridge, corn flakes, instant oatmeal, laba porridge, babao porridge |
| Salty snacks | Corn crisps, onion rings, potato chips, |
| Candy, sugar and other high-sugar foods | Jelly, jam, chocolate, honey, sugar, candies |

**Table S4 (continued)**

| **Food group** | **Examples of food items** | |
| --- | --- | --- |
| Calorically-sweetened beverages | Fruit or flavored drinks, fruit juice, soft drinks, oiled tea |  |
| Low-caloric beverages | Tea, bottled water |  |
| Alcoholic beverages | Liquors, wine, vodka, cocktails, whiskey, beer, millet wine |  |
| Food supplement/functional food | Bird’s nest |  |
| Oils | Seeds oil, olive oil, vegetable oil |  |
| Condiments | Salt, vinegar |  |

 *Popkin BM, Lu B, Zhai F. Understanding the nutrition transition: measuring rapid dietary changes in transitional countries. Public Health Nutr. 2002;5(6A):947–53.

**Supplementary materials I:**

**Table S5. Adapted Newcastle-Ottawa scale for observational studies**

1. **Newcastle - Ottawa quality assessment scale, adapted for case control studies included in this review**

**Selection (maximum 6 points):**

1. Is the case definition adequate?

a. Yes, with independent validation (one star)

b. Yes, e.g. record linkage/medical record or based on self-reports

c. No description

2. Representativeness of the cases

a. Consecutive or obviously representative series of cases (one point)

b. Potential for selection biases or not stated

3. Selection of Controls

a. Community controls (one point)

b. Hospital controls

c. No description

4. Definition of Controls

a. No history of disease (endpoint) (one point)

b. No description of source

5. Ascertainment of exposure (risk factor)

a. Validated measurement tool. (two point)

b. Non-validated measurement tool, but the tool is available or described. (one point)

c. No description of the measurement tool.

**Comparability (maximum 2 points):**

1. Comparability of cases and controls on the basis of the design or analysis

a. Study controls for age, sex, BMI, and/or health status (one point)

b. Study controls for any additional factor b) including smoking, physical activity level, education level and family income (one point)

c. No information provided

**Exposure (maximum 3 points)**

1. Ascertainment of exposure

a. Secure record (e.g., medical records, independent medical assessment). (one point)

b. Structured interview where blind to case/control status. (one point)

c. Interview not blinded to case/control status

d. Written self-report or medical record only

e. No description

2. Same method of ascertainment for cases and controls

a. Yes (one point)

b. No

3. Non-response rate

a. Same rate for both groups (one point)

b. Non respondents described

c. Rate different and no designation

**Overall:**

Low risk: 8-11 points

Moderate risk: 5-7 points

High risk: 0-4 points

1. **Newcastle - Ottawa quality assessment scale, adapted for cohort studies included in this review**

**Selection (maximum 4 points)**

1. Representativeness of the exposed cohort

a. Truly representative (one point)

b. Somewhat representative (one point)

c. Selected group

d. No description of the derivation of the cohort

2. Selection of the non-exposed cohort

a. Drawn from the same community as the exposed cohort (one point)

b. Drawn from a different source

c. No description of the derivation of the non-exposed cohort

3. Ascertainment of exposure

a. Secure record (e.g., FFQ, family food investigation) (one point)

b. Structured interview (one point)

c. Written self-report

d. No description

e. Other

4. Demonstration that outcome of interest was not present at point of study

a. Yes (one point)

b. No

**Comparability (maximum 2 points)**

1. Comparability of cohorts on the basis of the design or analysis controlled for confounders Model 1 was adjusted for age and gender, Model 2 was further adjusted for education, income, urbanization, smoking, alcohol drinking, and physical activity, and Model 3 was further adjusted for BMI and hypertension

a. The study controls for age, sex, BMI, and/or health status (one point)

b. Study controls for other factors, including smoking, physical activity level, education level and family income (one point)

c. No information provided

**Outcome (maximum 3 points)**

1. Assessment of outcome

a. Independent medical assessment (one point)

b. Record linkage (one point)

c. Self-report

d. No description

e. Other

2. Was follow-up long enough for outcomes to occur

a. Yes (one point) 3 years 2006-2009

b. No

Indicate the median duration of follow-up and a brief rationale for the assessment above: _________________

3. Adequacy of follow-up of cohorts

a. Complete follow up- all subject accounted for (one point)

b. Subjects lost to follow up unlikely to introduce bias- number lost less than or equal to 20% or description of those lost suggested no different from those followed. (one point)

c. Follow up rate less than 80% and no description of those lost

d. No statement

**Overall:**

Low risk: 3 or 4 points in selection domain AND 1 or 2 points in comparability domain AND 2 or 3 points in outcome/exposure domain

Moderate risk: 2 points in selection domain AND 1 or 2 points in comparability domain AND 2 or 3 points in outcome/exposure domain

High risk: 0 or 1 point in selection domain OR 0 points in comparability domain OR 0 or 1 points in outcome/exposure domain

**c. Newcastle - Ottawa quality assessment scale, adapted for cross-sectional studies included in this review***

**Selection: (Maximum 5 points)**

1. Representativeness of the sample:

a. Truly representative of the average in the target population. (all subjects or random sampling or using a large sample from the CHNS) (one point)

b. Somewhat representative of the average in the target group. (non-random sampling) (one point)

c. Selected group of users/convenience sample.

d. No description of the derivation of the included subjects.

2. Sample size:

a. Justified and satisfactory (including sample size calculation) (one point)

b. Not justified.

c. No information provided

3. Non-respondents:

a. Proportion of target sample recruited attains pre-specified target or basic summary/description of non-respondent characteristics in sampling frame recorded. (one point)

b. Unsatisfactory recruitment rate, no summary/description data on non-respondents.

c. No information provided

4. Ascertainment of the exposure (risk factor):

a. Validated measurement tool (e.g. Valided FFQ). (two point)

b. Non-validated measurement tool, but the tool is available or described. (one point)

c. No description of the measurement tool.

**Comparability (Maximum 2 points)**

1.Comparability of subjects in different outcome groups on the basis of design or analysis. Confounding factors controlled.

a. Data/ results adjusted for age, sex, BMI, and/or health status(one point)

b. Data/results adjusted for smoking, physical activity level, education level and family income (one point)

c. No information provided

**Outcome: (maximum 3 points)**

1. Assessment of outcome:

a. Independent blind assessment using objective validated laboratory methods or medical diagnosis.(two points)

b. Unblinded assessment using objective validated laboratory methods or or medical diagnosis. (two points)

c. Used non-standard or non-validated laboratory methods with gold standard or self-reported (one point).

d. No description/non-standard laboratory methods used.

2. Statistical test:

a. Statistical test used to analyse the data clearly described, appropriate and measures of association presented including confidence intervals and probability level (p value and 95%CI). (one point)

b. Statistical test not appropriate, not described or incomplete.

**Overall**

Low risk: 8-10 points

Moderate risk: 5-7 points

High risk: 0-4 points

*This scale has been adapted from the Newcastle-Ottawa quality assessment scale for cohort studies to provide quality assessment of cross sectional studies.

**Supplementary materials I**

**Table S6. List of excluded studies (n=97)**

| **Author (year)** | **Title** | **Reason for exclusion** |
| --- | --- | --- |
| Liguori (2013) | Effect of a basic Chinese traditional diet in overweight patients | Duplicate report |
| Ali A et al. (2020) | Exploration of the Principal Component Analysis (PCA) Approach in Synthesizing the Diet Quality of the Malaysian Population | No description of the TCD |
| Ali Asma’ et al. (2020) | Exploration of the Principal Component Analysis (PCA) Approach in Synthesizing the Diet Quality of the Malaysian Population | No description of the TCD |
| Balasubramanian G. V. et al. (2020) | Associations of Eating Mode Defined by Dietary Patterns with Cardiometabolic Risk Factors in the Malaysia Lipid Study Population | Diet identified could not be recognised as characteristics of China |
| Batis and Carolina (2013) | Dietary pattern trajectories over time and diabetes among Chinese adults | Duplicate report |
| Cao et al.(2021) | Association between dietary patterns and risk of breast cancer in Chinese female population: a latent class analysis | Duplicate report |
| Chau and Christina (2019) | The Chinese traditional diet: a socioecological approach | Focus on single nutrient/item instead of the whole diet |
| Chen et al. (2018) | Association between Dietary Patterns and Precocious Puberty in Children: A Population-Based Study | Population were not adults or population were breastfeeding women |
| Chen and Xu (1996) | Historical development of Chinese dietary patterns and nutrition from the ancient to the modern society | No description of TCD |
| Chen and Gao (1993) | The Chinese total diet study in 1990. Part II. Nutrients | Focus on single nutrient/item instead of the whole diet |
| Du et al. (2017) | Association of Dietary Pattern during Pregnancy and Gestational Diabetes Mellitus: A Prospective Cohort Study in Northern China | Population were not adults or population were breastfeeding women |

**Table S6 (continued)**

| **Author (year)** | **Title** | **Reason for exclusion** |
| --- | --- | --- |
| Fang et al. (2020) | Association between dietary pattern and the risk of type 2 diabetes mellitus in Zhejiang Province, China: A case-control study | Duplicate report |
| Fox et al. (2018) | The Low- FODMAP diet and traditional dietary advice reduce symptoms of diarrhea predominant irritable bowel syndrome in Chinese population: A randomized controlled trial with analysis to identify factors associated with efficacy | Diet identified could not be recognised as characteristics of China |
| He et al. (2016) | Association between predominantly plant-based diets and iron status in Chinese adults: a cross-sectional analysis | No description of TCD |
| He et al. (2012) | Association between high fat-Low carbohydrate diet score and newly diagnosed type 2 diabetes in Chinese population | No description of TCD |
| Ho et al. (2011) | The Association between Traditional Chinese Dietary and Herbal Therapies and Uterine Involution in Postpartum Women | No description of TCD |
| Hong et al. (2016) | Dietary patterns and the incidence of hyperglyacemia in China | Duplicate report |
| Hsu et al. (2014) | Associations between dietary patterns and kidney function indicators in type 2 diabetes | Diet identified could not be recognised as characteristics of China |
| Huang et al. (2019) | Blood biomarkers of various dietary patterns correlated with metabolic indicators in Taiwanese type 2 diabetes | Diet identified could not be recognised as characteristics of China |

**Tables S6 (continued)**

| **Author (year)** | **Title** | **Reason for exclusion** |
| --- | --- | --- |
| Huang et al. (2012) | Association between dietary patterns and renal function indicators in type 2 diabetes | No description of TCD |
| Jia et al. (2010) | Traditional Cantonese diet and nasopharyngeal carcinoma risk: a large-scale case-control study in Guangdong, China | Focus on single nutrient/item instead of the whole diet |
| Khor et al. (1998) | Dietary practices in nutritional transition: The case of Malaysian urban Chinese | No description of TCD |
| Laura et al. (2012) | Traditional Chinese diet (TCD) by the TCM-CAO method: Clinical cases | Diet identified could not be recognised as characteristics of China |
| Leung et al. (2000) | Fat intake in Hong Kong Chinese children | Population were not adults or population were breastfeeding women |
| Li et al. (2018) | Dietary pattern is associated with overweight/obesity among school-aged children in Southwest China | Population were not adults or population were breastfeeding women |
| Li et al. (2021) | Dietary patterns of Chinese puerperal women and their association with postpartum weight retention: Results from the mother-infant cohort study | Population were not adults or population were breastfeeding women |
| Liou et al. (2018) | Dietary Acculturation of Obesity Risk Reduction Behaviours in Chinese Americans | No description of TCD |
| Liu et al. (2017) | The Significance of Restoring the Traditional Diet of Chinese People from the High Incidence of Modern Diseases | Focus on single nutrient/item instead of the whole diet |

**Tables S6 (continued)**

| **Author (year)** | **Title** | **Reason for exclusion** |
| --- | --- | --- |

| Liu et al. (2018) | Dietary Patterns and Association with Obesity of Children Aged 6⁻17 Years in Medium and Small Cities in China: Findings from the CNHS 2010⁻2012 | Population were not adults or population were breastfeeding women |
| --- | --- | --- |
| Liu et al. (1994) | The relationship between dietary factors and serum lipids in southern Chinese population samples | Focus on single nutrient/item instead of the whole diet |
| Liu et al. (2018) | An observational study on the association between major dietary patterns and non-alcoholic fatty liver disease in Chinese adolescents | Population were not adults or population were breastfeeding women |
| Lu et al. (2016) ^S35^ | Maternal Dietary Patterns and Fetal Growth: A Large Prospective Cohort Study in China | Diet identified could not be recognised as characteristics of China |
| Lu et al. (2017)^71^ | The association of dietary pattern and breast cancer in Jiangsu, China: A population-based case-control study | Duplicate report |
| Mao et al. (2016) | Self-reported health problems related to traditional dietary practices in postpartum women from urban, suburban and rural areas of Hubei province, China: the 'zuòyuèzi' | Population were not adults or population were breastfeeding women |
| Mau and Ching (2021) | Dietary Patterns among East Asian Children Living in the US Affiliated Pacific Region | Population were not adults or population were breastfeeding women |
| Min et al. (2021) | Dietary patterns and their associations with overweight/obesity among preschool children in Dongcheng District of Beijing: a cross-sectional study | Population were not adults or population were breastfeeding women |
| Newman and Jacqueline (1980) | Chinese immigrant food habits: a study of the nature and direction of change | No description of TCD |
| Parpia and Banoo (1995) | Socioeconomic determinants of food and nutrient intakes in rural China | No description of TCD |

**Tables S6 (continued)**

| **Author (year)** | **Title** | **Reason for exclusion** |
| --- | --- | --- |

| Rutayisire et al. (2018) | Dietary patterns are not associated with overweight and obesity in a sample of 8900 Chinese preschool children from four cities | Population were not adults or population were breastfeeding women |
| --- | --- | --- |
| Shen et al. (2021) | Association between dietary patterns and glycaemic control in a middle-aged Chinese population | Duplicate report |
| Song and Jiang (2015) | Association between sleep and dietary patterns in preschool children | Population were not adults or population were breastfeeding women |
| Su et al. (2012) | Study on dietary patterns and its effect on infant health among left-behind children aged 1-4 years old with both parents working out in rural Anhui | Population were not adults or population were breastfeeding women |
| Tan et al. (2021) | Dietary quality is associated with reduced risk of diabetes among adults in Northern China: a cross-sectional study | Duplicate report |
| Teo et al. (2018) | Prospective Associations of Maternal Dietary Patterns and Postpartum Mental Health in a Multi-Ethnic Asian Cohort: The Growing up in Singapore towards Healthy Outcomes (GUSTO) Study | No description of TCD |
| Thompson et al. (2010) | Maternal dietary patterns in pregnancy and the association with small-for-gestational-age infants. | Diet identified could not be recognised as characteristics of China |
| Wang et al. (2017) | Optimal dietary macronutrient distribution in China (ODMDC): a randomised controlled-feeding trial protocol | Focus on single nutrient/item instead of the whole diet |
| Wang et al. (2011) | Dietary patterns and hypertension among Chinese adults: a nationally representative cross-sectional study | Duplicate report |

**Tables S6 (continued)**

| **Author (year)** | **Title** | **Reason for exclusion** |
| --- | --- | --- |

| Wang et al. (2015) | Dietary patterns and cardio-cerebrovascular disease in a Chinese population | Duplicate report |
| --- | --- | --- |
| Wang et al. (2012a) | Influence of different dietary patterns on bone mineral density and body mass index of college freshmen in urban and rural areas of China | Duplicate report |
| Wang et al.(2012b) | Dietary patterns in college freshmen and its relation to bone mineral density | Duplicate report |
| Wang et al.(2012c) | Is there any relationship between dietary patterns and depression and anxiety in Chinese adolescents? | Population were not adults or population were breastfeeding women |
| Wesley and Dearth (2012) | Comparison of dietary and physical activity behaviors and their associations with body mass index in Chinese mothers and children | Population were not adults or population were breastfeeding women |
| Whang (1981) | Chinese traditional food therapy | Diet identified could not be recognised as characteristics of China |
| Wilson et al. (2020) | Diet and the Human Gut Microbiome: An International Review | Focus on single nutrient/item instead of the whole diet |
| Woo et al. (2003) | Diet and glucose tolerance in a Chinese population | No description of TCD |

**Tables S6 (continued)**

| **Author (year)** | **Title** | **Reason for exclusion** |
| --- | --- | --- |
| Wu-Jung and C.J(1994) | Understanding food habits of Chinese Americans | Focus on single nutrient/item instead of the whole diet |
| Xia et al. (2020) | Intermediary effect of inflammation on the association between dietary patterns and non-alcoholic fatty liver disease | Diet identified could not be recognised as characteristics of China |
| Yan et al.(2020a) | Dietary patterns of Chinese women of childbearing age during pregnancy and their relationship to the neonatal birth weight | Population were not adults or population were breastfeeding women |
| Yan et al.(2020b) | Differential effects of Chinese high-fat dietary habits on lipid metabolism: mechanisms and health implications | Population were not adults or population were breastfeeding women |
| Ye et al. (2019) | Joint associations of dietary pattern and television viewing with CVD risk factors among urban men and women in China: a cross-sectional study | No description of TCD |
| Qin (2012) | Zinc Intake and Dietary Pattern in Jiangsu Province, China: Consequences of Nutrition Transition | Duplicate report |
| Yu et al. (2019) | Association between dietary magnesium and the risk of type 2 diabetes in Harbin residents | Full text could not be available |

**Tables S6 (continued)**

| **Author (year)** | **Title** | | | **Reason for exclusion** |
| --- | --- | --- | --- | --- |
| Zhai et al. (2013) | The evolution of the Chinese diet: 1991-2011 | | | Focus on single nutrient/item instead of the whole diet |
| Zhang et al. (2015a) | Dietary patterns and their associations with childhood obesity in China | | | Population were not adults or population were breastfeeding women |
| Zhang et al.(2015b) | The Difference in Nutrient Intakes between Chinese and Mediterranean, Japanese and American Diets | | | Focus on single nutrient/item instead of the whole diet |
| Zhao et al. (2022) | The Association between Postpartum Practice and Chinese Postpartum Depression: Identification of a Postpartum Depression-Related Dietary Pattern | | | Population were not adults or population were breastfeeding women |
| Zhen et al. (2018) | Dietary pattern is associated with obesity in Chinese children and adolescents: data from China Health and Nutrition Survey (CHNS) | | | Population were not adults or population were breastfeeding women |
| Zou et al. (2021) | Associations between dietary patterns and anaemia in 6- to 23-month-old infants in central South China | | | Population were not adults or population were breastfeeding women |
| He (2016) | 日常传统饮食对乳腺癌风险影响的研究现状 | | | No description of TCD |
| Ling (2014) | | 节气饮食:被遗忘的传统食之道 | No description of TCD | |

**Tables S6 (continued)**

| **Author (year)** | **Title** | | | | **Reason for exclusion** |
| --- | --- | --- | --- | --- | --- |
| Liu (2010) | | 中国传统饮食与西方现代饮食的差异 | No description of TCD | | |
| Bian (2013) | | 产后营养饮食与传统饮食的比较研究 | Focus on single nutrient/item instead of the whole diet | | |
| Lv et al. (2003) | | 一种2型糖尿病的新型饮食疗法 | No description of TCD | | |
| Song (2003) | | 剖宫产患者术后首次进普通饮食与传统饮食的对照研究 | Population were not adults or population were breastfeeding women | | |
| Zhang (2019) | | 传统与现代:民国时期太原市民的饮食 | Focus on single nutrient/item instead of the whole diet | | |
| Shi (2007) | | 传统饮食习惯惹的祸 | Focus on single nutrient/item instead of the whole diet | | |
| Li et al. (2019) | | 日常传统饮食对肝癌风险影响的研究现状 | Focus on single nutrient/item instead of the whole diet | | |
| Li (2012) | | 中华传统饮食文化与现代饮食营养 | | Focus on single nutrient/item instead of the whole diet | |
| Zhi (2004) | | 传统饮食观未必可靠 | | Focus on single nutrient/item instead of the whole diet | |
| Wang (2018) | | 对于心脏病患者来说,传统的北方饮食是不利的 | | No description of TCD | |

**Tables S6 (continued)**

| **Author (year)** | **Title** | | | **Reason for exclusion** |
| --- | --- | --- | --- | --- |
| Wang (2002) | | 从传统文化看中国饮食 | Focus on single nutrient/item instead of the whole diet | |
| Bai (2021) | | 中华传统饮食文化在海外传播的影响研究——评《中国文化系列丛书：中国文化·饮食》 | No description of TCD | |
| Bai and Si (2020) | | 蒙古族传统饮食伦理研究 | Focus on single nutrient/item instead of the whole diet | |
| Guan (2003) | | 餐前保健挑战传统饮食 | No description of TCD | |
| Ke D (2007) | | 传统饮食习惯中的健康误区 | Focus on single nutrient/item instead of the whole diet | |
| Zhao (2007) | | 让“谷香”重回您的餐桌 | Focus on single nutrient/item instead of the whole diet | |
| Guo (2009) | | 中国的传统饮食 | Focus on single nutrient/item instead of the whole diet | |
| Chen (2001) | | 中国、亚洲和地中海传统饮食模式国际研讨会在京召开 | No description of TCD | |
| Chen and Ma (20021) | | 从传统饮食习惯和当今饮食潮流看高校食堂膳食供给改革 | Focus on single nutrient/item instead of the whole diet | |
| Gao and Wen (2020) | | 孕期行为因素对婴儿超重肥胖影响的研究 | Population were not adults or population were breastfeeding women | |

**Tables S6 (continued)**

| **Author (year)** | **Title** | | | **Reason for exclusion** |  |
| --- | --- | --- | --- | --- | --- |
| Qi et al. (2008) | | 饮食与肿瘤 | No description of TCD | |  |
| Gong (2001) | | 食补雌激素改善更年期 | No description of TCD | |  |
| Gong (2013) | | 内蒙古通辽地区蒙古民族饮食习惯与食管癌的相关研究 | No description of TCD | |  |
| NR | | 坚持传统饮食结构提高全民健康素质 | Focus on single nutrient/item instead of the whole diet | | |
| Lv et al. (1998) | | 传统饮食习惯最健康 | Focus on single nutrient/item instead of the whole diet | | |
| Nong et al. (2004) | | 不益健康的传统饮食 | Focus on single nutrient/item instead of the whole diet | | |

**Supplementary materials I: Table S7. Characteristics of all included studies (n=99)***

| **Author/Year** | **Country** | **Study design** | **Years/**  **period represented** | **Geographical location represented** | **Ethnicity** | **Method used to define diet** | **Definition of diet** |
| --- | --- | --- | --- | --- | --- | --- | --- |
| Batis et al. (2014) ^S1^ | China | Cohort study | 1991-2009 | Whole country | HAN | FA | Traditional southern diet; traditional northern diet |
| Batis et al. (2016) ^S2^ | United States | Cohort study | 2006-2009 | Whole country | NR | PCA | Traditional southern diet |
| Batis Ruvalcaba (2013) ^S3^ | China | Cohort study | 1991-2009 | Whole country | HAN | FA | Traditional southern pattern |
| Cao et al. (2020a) ^S4^ | China | Cross-sectional study | 2013 | Jiangsu Province, eastern China | HAN | PCA | TCD |
| Cao et al. (2020b) ^S5^ | Australia | Cohort study | 1991-2009 | Whole country | NR | FA | TCD |
| Cao et al. (2021) ^S6^ | China | Case-control study | 2013 | Wuxi, Jiangsu Province, eastern China | HAN | Latent class analysis | TCD |
| Chen (2005) ^S7^ | China | Editorial | NR | Whole country | NR | Descriptive method | TCD |
| Chen et al. (2017)^S8^ | China; Taiwan | Prospective cohort study | 2011-2015 | Taiwan, China | HAN | FA | TCD |
| Chen et al. (2020)^S9^ | China | Cross-sectional, case-control study | 2013-2016 | Haikou, Hainan Province, eastern China | HAN | PCA | TCD |

**Tables S7 (continued)**

| **Author/Year** | **Country** | **Study design** | **Years/**  **period represented** | **Geographical location represented** | **Ethnicity** | **Method used to define diet** | **Definition of diet** |
| --- | --- | --- | --- | --- | --- | --- | --- |
| Chung et al. (2018)^S10^ | Australia | Cohort study | 2008-2010 | Taiwan | NR | PCA | TCD |
| Du et al. (2001)^S11^ | China | Literature review | NR | Whole country | HAN | Historical data | TCD |
| Fan et al. (2016)^S12^ | China | Randomised, single-blind, multi-arm parallel trial | NR | Whole country | NR | PCA | TCD |
| Fang (1999)^S13^ | China | Editorial | NR | Whole country | NR | Descriptive methods | TCD |
| Fu (2015)^S14^ | China | Editorial | NR | Whole country | HAN | Descriptive method | TCD |
| Guo et al. (2020)^S15^ | China | Cross-sectional study | 2012 | Whole country | HAN:95%, Other:5% | PCA | TCD |
| He et al. (2017)^S16^ | China | Cross-sectional study | 2014-2016 | Hangzhou, Zhejiang Province, eastern China | HAN | PCA | TCD |
| Hong et al. (2013)^S17^ | China | Cohort study | 2007-2010 | Nanjing, eastern China | NR | PCA | TCD |
| Hong et al. (2016)^S18^ | China | Cross-sectional study | 2014 | Nanjing, eastern China | HAN | FA | TCD |
| Hu et al. (2019)^S19^ | China | Cross-sectional study | 2017 | Shenyang, northern China | HAN:77.9%, Minority:22.1% | PCA | TCD |

**Tables S7 (continued)**

| **Author/Year** | **Country** | **Study design** | **Years/**  **period represented** | **Geographical location represented** | **Ethnicity** | **Method used to define diet** | **Definition of diet** |
| --- | --- | --- | --- | --- | --- | --- | --- |
| Koo (1976)^S20^ | China | Literature review | NR | Whole country | NR | Historical data | Traditional northern diet; traditional southern diet |
| Lan et al. (2018)^S21^ | China | Case-control study | 2018 | Guangzhou, eastern China | HAN | PCA | Traditional Cantonese dietary pattern |
| Leonetti et al. (2016)^S22^ | Italy | RCT | 2018 | Whole country | NR | List foods in daily receipt | TCD |
| Li (2011)^S23^ | China | Literature review | NR | Near the Wuling Mountain, central China | Tu Jia | Historical and ethnographic data | Traditional Tujia diet (minority group) |
| Li and Shi (2017)^S24^ | Australia | Cohort study | 1991-2011 | Whole country | NR | PCA | TCD |
| Li et al. (2011)^S25^ | China | Cross-sectional study | 2002 | Whole country | NR | FA combined with cluster analysis | Traditional northern diet; traditional southern diet |
| Li et al. (2019)^S26^ | China | Cross-sectional study | 2016-2018 | Hangzhou, Zhejiang Province, eastern China | HAN | PCA | Traditional southern diet |
| Li et al. (2021)^S27^ | China | Cross-sectional study | 2017 | Xinxiang, Henan, northern China | NR | PCA | TCD |

**Tables S7 (continued)**

| **Author/Year** | **Country** | **Study design** | **Years/**  **period represented** | **Geographical location represented** | **Ethnicity** | **Method used to define diet** | **Definition of diet** |
| --- | --- | --- | --- | --- | --- | --- | --- |
| Li et al. (2022)^S28^ | China | Cross-sectional study | 2018 | Sichuan, central China | NR | FA | TCD |
| Liang (2022)^S29^ | China | Literature review | Shang dynasty; Northern Song dynasty to mid-Qing dynasties | Whole country | NR | Historical data | TCD |
| Liao et al. (2019)^S30^ | China | Cross-sectional study | 2013-2016 | Tianjin, northern China | HAN | PCA | Traditional Tianjin diet |
| Lin et al. (2019)^S31^ | China | Cross-sectional study | 2018 | Binzhou, Shandong, northern China | HAN | PCA | TCD |
| Liu and Li (2000)^S32^ | China | Cross-sectional epidemiological and nutritional research | 1994 | Meixian, Guangdong Province, eastern China | Hakka | NR | Traditional minority diet (Hakka) |
| Liu et al. (2021a)^S33^ | China | Cross-sectional study | 2019 | Gongcheng Yao Autonomous County, Guangxi, southern China | HAN | FA | TCD |
| Liu et al. (2021b)^S34^ | China | Cross-sectional study | 2017-2019 | Tianjin, northern China | NR | FA | Traditional Tianjin diet |

**Tables S7 (continued)**

| **Author/Year** | **Country** | **Study design** | **Years/**  **period represented** | **Geographical location represented** | **Ethnicity** | **Method used to define diet** | **Definition of diet** |
| --- | --- | --- | --- | --- | --- | --- | --- |
| Lu et al. (2016)^S35^ | China | Population-based case-control study | 2013-2014 | Wuxi, Jiangsu Province, eastern China | HAN | PCA | TCD |
| Lyu et al. (2014)^S36^ | China | Cross-sectional study | 2010 | Jiangsu, eastern China | HAN | PCA | TCD |
| Melaku et al. (2017)^S37^ | Australia | Cohort study | 1989-2011 | Whole country | HAN | PCA | TCD |
| Mi et al. (2017)^S38^ | China | Cross-sectional study | 2015 | Yantai, Shandong Province, northern China | HAN | PCA | TCD |
| Mu et al. (2014)^S39^ | China | Cross-sectional study | 2010 | Hefei, Anhui Province, eastern China | HAN | FA | TCD |
| Qi Mudedaoerji (2002)^S40^ | China | Literature review | Early 13th century | Menggu Province, northern China | Mongolian | Historical data | Traditional minority diet (Mongolian) |
| Qin et al. (2012)^S41^ | China | Cross-sectional study | 2002 | Jiangsu Province, eastern China | HAN | PCA | TCD |
| Qin et al. (2014)^S42^ | China | Cross-sectional study | 2002 | Jiangsu, eastern China | HAN | PCA | TCD |

**Table S7 (continued)**

| **Author/Year** | **Country** | **Study design** | **Years/**  **period represented** | **Geographical location represented** | **Ethnicity** | **Method used to define diet** | **Definition of diet** |
| --- | --- | --- | --- | --- | --- | --- | --- |
| Qin et al. (2021)^S43^ | China | Cohort study | 2004-2008 | Whole country | NR | PCA | Traditional northern diet |
| Ru et al. (2021)^S44^ | China | Case-control study | 2016-2018 | Hangzhou, Zhejiang Province, eastern China | NR | Clustering analysis | TCD |
| Shen et al. (2020)^S45^ | China | Cross-sectional study | 2016 | Hangzhou, Zhejiang Province, eastern China | HAN | PCA | Traditional southern diet |
| Shen et al. (2021)^S46^ | China | Cross-sectional study | 2016 | Hangzhou, Zhejiang Province, eastern China | HAN | PCA | Traditional southern diet |
| Shi (2021) ^S47^ | Australia | Cross-sectional study | 2009 | Whole country | NR | PCA | Traditional southern diet |
| Shi and Ganji (2020)^S48^ | Australia | Prospective cohort study | 1991-2011 | Whole country | NR | FA | TCD |
| Shi et al. (2006)^S49^ | Australia | Cross-sectional study | 2002 | Jiangsu Province, eastern China | NR | PCA | TCD |
| Shi et al. (2011)^S50^ | Australia | Cohort study | 2002-2007 | Jiangsu Province, eastern China | NR | PCA | TCD |

**Table S7 (continued)**

| **Author/Year** | **Country** | **Study design** | **Years/**  **period represented** | **Geographical location represented** | **Ethnicity** | **Method used to define diet** | **Definition of diet** |
| --- | --- | --- | --- | --- | --- | --- | --- |
| Shi et al. (2012)^S51^ | Australia | Cohort study | 2002-2007 | Jiangsu Province, eastern China | NR | PCA | TCD |
| Shi et al. (2018)^S52^ | Australia | Cross-sectional study | 2009 | Whole country | NR | PCA | Traditional southern diet |
| Shu et al. (2015)^S53^ | China | Cross-sectional study | July 2014 through June 2015 | Hangzhou, Zhejiang Province, eastern China | HAN | PCA | TCD |
| Shu et al. (2017)^S54^ | China | Cross-sectional study | 2015 | Hangzhou, Zhejiang Province, eastern China | HAN | PCA | Traditional southern diet |
| Sun et al. (2013)^S55^ | Australia | Cross-sectional study | 2012 | Changshu, eastern China | NR | FA | TCD |
| Tan et al. (2021)^S56^ | China | Cross-sectional study | 2015 | Mongolia, northern China | Han:80.8%, Mongolian:15.6%  Other minority: 3.6% | PCA | Traditional northern diet |
| Tang et al. (2020)^S57^ | China | Cross-sectional study | 2011 | Whole country | NR | PCA | Traditional southern diet; traditional northern diet |
| Tian et al. (2011)^S58^ | China | Cross-sectional study | 2002 | Jiangsu Province, eastern China | NR | Clustering analysis | TCD |

**Table S7 (continued)**

| **Author/Year** | **Country** | **Study design** | **Years/**  **period represented** | **Geographical location represented** | **Ethnicity** | **Method used to define diet** | **Definition of diet** |
| --- | --- | --- | --- | --- | --- | --- | --- |
| Wang (1994)^S59^ | China | Editorial | NR | Whole country | NR | Descriptive method | TCD |
| Wang et al. (2011)^S60^ | China | Cross-sectional study | 2002 | Whole country | NR | PCA | Traditional northern diet; traditional southern diet |
| Wang et al. (2014)^S61^ | China | Cross-sectional study | 2013 | Baoji City, northern China | HAN | PCA | TCD |
| Wang et al. (2018)^S62^ | China | Cross-sectional study | 2016 | Linyi, Shandong Province, northern China | HAN | PCA | TCD |
| Wang et al. (2020a)^S63^ | China | Literature review | Early 1960s to late 1970s | Around the downstream reaches of the Yangtze River, eastern China | HAN | Historical data and ethnographic data | TCD; traditional southern diet |
| Wang et al. (2020b)^S64^ | China | Cross-sectional study | 2015 | Inner Mongolia, northern China | Han:81.78%, Mongolian: 14.56%,Other minorities:3.66% | PCA | Traditional northern diet |
| Wang et al. (2021)^S65^ | China | Cross-sectional study | 2014 | Jiangsu Province, eastern China | NR | EFA and CFA | TCD |

**Table S7 (continued)**

| **Author/Year** | **Country** | **Study design** | **Years/**  **period represented** | **Geographical location represented** | **Ethnicity** | **Method used to define diet** | **Definition of diet** |
| --- | --- | --- | --- | --- | --- | --- | --- |
| Wang et al. (2022)^S66^ | China | Cross-sectional study | 2014 | Jiangsu Province, eastern China | NR | FA | Traditional Jiangsu diet |
| Wei et al. (2018)^S67^ | China | Cross-sectional study | 2015 | Linyi, Shandong Province, northern China | HAN | PCA | TCD |
| Xu et al. (2015)^S68^ | Australia | Cross-sectional study | 2009 | Whole country | NR | PCA | TCD |
| Xu et al. (2016)^S69^ | Australia | Cohort study | 2004-2011 | Whole country | HAN | PCA | TCD |
| Xu et al. (2017)^S70^ | Australia | Cross-sectional study | 2009 | Whole country | HAN | PCA | TCD |
| Xu et al. (2018a)^S71^ | Australia | Cross-sectional study | 2009 | Whole country | NR | FA | TCD |
| Xu et al. (2018b)^S72^ | Australia | Cohort study | 1997-2006 | Whole country | HAN | FA | TCD |
| Xu et al. (2020)^S73^ | China | Cross-sectional study | 2016 | Hangzhou, Zhejiang Province, eastern China | HAN | PCA | Traditional southern diet |
| Xu et al. (2021)^S74^ | China | Cross-sectional study | 2015 | Linyi, Shandong Province, northern China | HAN | PCA | TCD |

**Table S7 (continued)**

| **Author/Year** | **Country** | **Study design** | **Years/**  **period represented** | **Geographical location represented** | **Ethnicity** | **Method used to define diet** | **Definition of diet** |
| --- | --- | --- | --- | --- | --- | --- | --- |
| Xue et al. (2017)^S75^ | China | Cross-sectional study | 2013 | Southern China | HAN | PCA | TCD |
| Yan et al. (2019)^S76^ | China | Cross-sectional study | 2013 | Shanxi Province, northern China | HAN | PCA | TCD |
| Yang et al. (2015)^S77^ | China | Cross-sectional study | 2012 | Hefei, Anhui Province, eastern China | NR | PCA | TCD |
| Yu et al. (2015)^S78^ | China | Cross-sectional study | 2004-2008 | Whole country | NR | FA combined with cluster analysis | Traditional southern diet; traditional northern diet |
| Yu et al. (2017)^S79^ | China | Cohort study | 2008-2013 | Whole country | HAN | PCA | Traditional northern diet |
| Yu et al. (2018)^S80^ | China | Cross-sectional study | 2016 | Linyi, Shandong Province, northern China | HAN | PCA | TCD |
| Yu et al. (2022)^S81^ | China | Cohort study | 2004-2008 | Whole country | NR | PCA | Traditional northern diet |
| Zeng et al. (2013)^S82^ | China | 1:1 age- (±3 years) and gender-matched case-control study | 2009-2012 | Guangdong Province, eastern China | HAN | PCA | TCD |

**Table S7 (continued)**

| **Author/Year** | **Country** | **Study design** | **Years/**  **period represented** | **Geographical location represented** | **Ethnicity** | **Method used to define diet** | **Definition of diet** |
| --- | --- | --- | --- | --- | --- | --- | --- |
| Zhang et al. (2015)^S83^ | China | Cross-sectional study | 2011 | Whole country | HAN | PCA | Traditional southern diet; traditional northern diet |
| Zhang et al. (2016a)^S84^ | Australia | Cross-sectional study | 2009 | Whole country | HAN | PCA | Traditional southern diet |
| Zhang et al. (2016b)^S85^ | China | Cross-sectional study | 2010-2012 | Yunnan, southern China | NR | FA | TCD |
| Zhang et al. (2017)^S86^ | China | Literature review | 1980-2016 | Whole country | HAN | Literature review | TCD |
| Zhang et al. (2020)^S87^ | China | Cross-sectional study | 2018 | Whole country | HAN | PCA | TCD |
| Zhang et al. (2021a)^S88^ | China | Cohort study | 1991-2015 | Whole country | HAN | FA | Traditional southern diet |
| Zhang et al. (2021b)^S89^ | China | Cross-sectional study | 2019 | Yunnan, southern China | A Chang: 33.6%, De Ang: 33.7%, Jing Po: 32.7% | PCA | TCD |
| Zhao (2000)^S90^ | China | Editorial | NR | Whole country | NR | Descriptive method | TCD |
| Zhao and Bao (2004)^S91^ | China | Literature review | NR | Whole country | NR | Historical data | TCD |
| Zhao and Bao (2009)^S92^ | China | Editorial | NR | Guang Xi, southern China | NR | Descriptive method | TCD |

**Table S7 (continued)**

| **Author/Year** | **Country** | **Study design** | **Years/**  **period represented** | **Geographical location represented** | **Ethnicity** | **Method used to define diet** | **Definition of diet** |
| --- | --- | --- | --- | --- | --- | --- | --- |
| Zheng et al. (2016)^S93^ | China | Cross-sectional study | 2015 | Hangzhou, Zhejiang Province, eastern China | HAN | PCA | TCD |
| Zhou (1998)^S94^ | China | Editorial | NR | Whole country | NR | Descriptive methods | TCD |
| Zhou et al. (2019a)^S95^ | China | Cross-sectional study | 2012 | Hefei, Anhui Province, eastern China | NR | FA | TCD |
| Zhou et al. (2019b)^S96^ | China | Cross-sectional study | 2012 | Hefei, Anhui Province, eastern China | HAN | FA | TCD |
| Zhou et al. (2021)^S97^ | China | Cross-sectional study | 2020 | Tibet, western China | HAN, Tibetan | PCA | TCD |
| Zuo et al. (2013)^S98^ | China | Cross-sectional study | 2006 | Jiangsu, eastern China | HAN | PCA | TCD |
| Zuo et al. (2014)^S99^ | China | Cross-sectional study | 2006 | Jiangsu Province, eastern China | NR | PCA | TCD |

CFA, Confirmatory factor analysis; EFA, Exploratory factor analysis; FA, Factor analysis; HAN, Han ethnicity; NA, Not reported; PCA, Principal component analysis; RCT, Randomised controlled trial; TCD, Traditional Chinese diet

* Reference list shown at the end of the supplementary material

**Supplementary materials I:**

**Table S8. Explanations for specific foods**

| **Food name** | **Explanation** |
| --- | --- |
| Babao porridge | A traditional Chinese porridge or congee made with a combination of eight precious ingredients. |
| Bing (with filling in it) | A plain or plain-flavored pastry without any filling |
| Bing (without filling in it) | A type of pastry that consists of a crust or shell filled with a variety of ingredients. |
| Ci | A traditional Chinese glutinous rice cake or rice ball commonly consumed as a dessert or snack |
| Dinggang | A traditional Chinese dish that is cooked and served in a special cooking vessel called "鼎罐" (dǐng guàn), combines rice, meat, vegetables, and seasonings |
| Gary tofu | A type of Chinese tofu that is traditionally made by fermenting tofu with ash |
| Köröngge | The cream’s top usually made by boiling cow's or sheep's milk until a thin layer form on the surface |
| Kumis | A traditional fermented dairy beverage, made from mare's milk, although variations using other types of milk, such as cow or goat milk, can also be found. |
| Laba porridge | A traditional Chinese dish that is mainly consumed on Laba Festival |
| Migao | A kind of food made from rice that has been processed in various ways. Depending on the region and culture, rice cakes can vary significantly in their preparation, texture, and taste. |
| Oil tea | A traditional beverage made by frying tea leaves in oil, adding water, and then boiling the mixture. |
| Tsampa | A traditional Chinese snack that made from glutinous rice flour which is often steamed, and then pounded or kneaded until it becomes a soft, sticky texture. |
| Zong | A traditional Chinese food made of glutinous rice stuffed with different fillings and wrapped in bamboo leaves. They are cooked by steaming or boiling. |

**Supplementary materials I:**

**Table S9. Amounts of food groups (reference list shown at the end of the supplementary material)**

1. **Amounts reported as percentages of food groups energy intake**

| **Food Groups** | **Ru et al. 2021^S44 *^** |
| --- | --- |
| **Rice** | 28.6 (18.7, 39.1) |
| **Congee** | 1.4 (0.2, 3.3)^*^ |
| **Wheat products** | 8.3 (4.0, 14.4)^*^ |
| **Desserts** | 2.6 (0.7, 8.6)^*^ |
| **Deep fried foods** | 0.9 (0.0, 2.4)^*^ |
| **Stuffed foods** | 3.2 (1.4, 6.2)^*^ |
| **Whole grain foods** | 2.2 (0.8, 5.7)^*^ |
| **Tubers and roots** | 0.7 (0.3, 1.3)^*^ |
| **Dairy products** | 1.5 (0.1, 5.0)^*^ |
| **Eggs** | 2.5 (1.1, 4.6)^*^ |
| **Red meat** | 8.1 (4.0, 13.6)^*^ |
| **Poultry** | 2.1 (0.9, 4.1)^*^ |
| **Processed me*t** | 0.5 (0.0, 1.3)^*^ |
| **Fresh water foods** | 1.2 (0.5, 2.0)^*^ |
| **Seafood** | 0.5 (0.2, 1.2)^*^ |
| **Soy products** | 2.3 (1.1, 4.3)^*^ |
| **Nuts** | 2.9 (0.8, 7.3)^*^ |
| **Dark-coloured vegetables** | 1.7 (1.1, 2.8)^*^ |
| **Light-coloured vegetables** | 0.6 (0.3, 1.2)^*^ |
| **Fungus** | 0.1 (0.0, 0.2)^*^ |
| **Fruits** | 2.6 (1.2, 4.2)^*^ |
| **Soft beverage** | 0.0 (0.0, 0.5)^*^ |
| **Beer** | 0.0 (0.0, 0.2)^*^ |
| **Red wine** | 0.0 (0.0, 0.1) |

*Results were expressed as median (first quartile, third quartile).

1. **Amounts reported as average daily food groups consumption (grams/day)**

| **Food Groups** | **Cao et al. 2021^S6^** | **Fan et al. 2016^S12^** | **Li et al. 2022^S28^*** | **Tian et al. 2011^S58^** | **Melaku et al. 2017^S37^*** |
| --- | --- | --- | --- | --- | --- |
| **Rice** | 280.10 | - | 126 (72) | - | 286.9 (211.4) |
| **Cereals** | 18.44 | 335 | 200 (63) | - | 26.7 (82.4) |
| **Wheat and Wheat products** | - | - | - | 488.36 | - |
| **Starchy and tuber** | - | 74 | - | 4.89 | - |
| **Dried legumes and products** | - | 34 | 23 (6) | 48.82 | 10.0 (26.3) |
| **Fruits** | 132.38 | 214 | 214 (32) | 6.55 | 19.4 (72.2) |
| **Fried food** | 1.93 | - | - | - | - |
| **Vegetables** | 57.912 | 528 | 500 (131) | 385.17 | 279.1(179.0) |
| **Nuts and seeds** | 11.46 | - | 31 (14) | 20.48 | - |
| **Livestock and its products** | 23.91 | 274 | 70 (31) | 64.96 | - |
| **Poultry and its products** | 9.35 |  | - | 21.18 | - |
| **Pickled foods** | 3.43 | - | - | - | 15.7(46.7) |
| **Aquatic** | 29.69 | 96 | 27 (13) | 52.49 | 24.1 (47.2) |
| **Dairy products** | 91.35 | 188 | 237 (43) | 5.89 | 5.8 (35.3) |
| **Soy foods** | 19.48 | - | - | - | 22.7(42.4) |
| **Egg and its products** | 33.58 | 51 | 38 (9) | 28.13 | - |
| **Cakes and snacks** | 10.05 | - | - | 4.51 | - |
| **Fresh juice** | 5.65 | - | - | - | - |
| **Soft drink** | 5.68 | - | - | 0.17 | - |
| **Oil** | - | 23 | - | - | - |

*Results were expressed as mean (standard deviation).

1. **Amounts reported as quartiles/quintiles daily consumed (grams/day)**

| **Food Groups** | **Zhou et al. 2019a^S95^*** | | | | | **Xu et al. 2020^S73^**** | | | |
| --- | --- | --- | --- | --- | --- | --- | --- | --- | --- |
| **Adherence to the TCD  (lowest adherence T1 to highest adherence T4/T5)** | **T1** | **T2** | **T3** | **T4** | **T5** | **T1** | **T2** | **T3** | **T4** |
| **Rice** | 181.4 (10.7, 221.4) | 246.4 (222.1, 278.6) | 314.3 (279.5, 348.6) | 402.3 (349.9, 453.2) | 528.6 (453.6, 1906.6) | 282 (149.9) | - | - | 365.8 (188.2) |
| **Cereals** | 0 (0, 25) | 42.9 (25.7, 60.7) | 89.3 (61.4, 109.3) | 128.6 (110.0, 171.4) | 226.8 (172.1, 1400.0) | 24.1 (6.4) | - | - | 26.9 (5.9) |
| **Wheat and Wheat products** | 90 (0, 150) | 185 (160.0, 190.0) | 224 (191, 250) | 305 (251, 375) | 400 (378, 1600) | - | - | - | - |
| **Starchy and tuber** | 0.2 (0, 1.7) | 2.9 (1.7, 7.1) | 8.9 (8.1, 10.7) | 18.2 (12.4, 21.4) | 42.9 (24.6, 1000.0) | 17 (12.5) | - | - | 20.9 (11.2) |
| **Dried legumes/soy foods** | 4.3 (0, 22.5) | 26.8 (22.6, 33.9) | 46.9 (34, 52.1) | 60.7 (52.2, 78.6) | 110.6 (79.3, 1750.0) | - | - | - | - |
| **Fried food** | 0.4 (0, 0.9) | 3.6 (1.1, 6.4) | 10.7 (7.1, 500) | – | – | - | - | - | - |
| **Fruits** | 6 (0, 21.4) | 42.9 (25.0, 64.3) | 98.2 (75.0, 117.9) | 150 (125.0, 196.4) | 175.4 (200.1, 500.0) | 67.3 (28.4) | - | - | 86.2 (23.1) |
| **Vegetables** | 0 (27.1, 241.4) | 282.1 (242.9, 328.6) | 371.4 (330.0, 417.9) | 454.5 (418.2, 535.4) | 671.4 (535.7, 4507.1) | 377.8 (135) | - | - | 408.2 (166.9) |
| **Cakes** | - | - | - | - | - | - | - | - | - |
| **Animal meat** | 33.9 (0, 5) | 61.6 (50.4, 72.3) | 85.7 (72.8, 100.0) | 121.4 (100.4, 150.0) | 197.8 (150.4, 1026.8) | 69.6 (39.2) | - | - | 65.5 (30.2) |

**Table S9. (continued)**

| **Food Groups** | **Zhou et al. 2019 ^S95^*** | | | | | **Xu et al. 2020^S73^**** | | | |
| --- | --- | --- | --- | --- | --- | --- | --- | --- | --- |
| **Adherence to the TCD**  (lowest adherence T1 to highest adherence T4/T5)** | **T1** | **T2** | **T3** | **T4** | **T5** | **T1** | **T2** | **T3** | **T4** |
| **Poultry and its products** | 8.6 (0, 42.9) | 57.1 (43.6, 64.3) | 75 (65.0, 85.7) | 110.7 (86.2, 132.1) | 175 (133.0, 1017.9) |  | - | - |  |
| **Pickled foods** | 0.1 (0, 0.9) | 2 (0.9, 5.0) | 8.6 (5.4, 17.9) | 22.2 (19.6, 25.0) | 30 (25, 400) | - | - | - | - |
| **Aquatic** | 0.4 (0, 10.3) | 15.2 (10.7, 21.4) | 25 (22.4, 32.1) | 38.4 (32.5, 50.9) | 75 (51.8, 1107.1) | 120.9 (35.5) | - | - | 144.9 (32.8) |
| **Dairy products** | 0.7 (0, 2.5) | 5.4 (2.6, 10) | 35.7 (10.7, 63.6) | 122.9 (64.3, 200.0) | 250 (203.6, 1000.0) | - | - | - | - |
| **Egg and its products** | - | - | - | - | - | 50.3 (13.2) | - | - | 71 (10.6) |
| **Soft drinks** | - | - | - | - | - | 10.6 (8.5) | - | - | 13.2 (7.1) |
| **Oil and fat** | 1.4 (0.1, 1.8) | 2.3 (2, 2.5) | 2.9 (2.7, 3.8) | 4.5 (4, 5) | 9 (5.5, 25.0) | - | - | - | - |

**Table S9. (continued)**

| **Food Groups** | **Xu et al., 2015^S68^** for women** | | | | **Xu et al., 2015^S68^** for men** | | | |
| --- | --- | --- | --- | --- | --- | --- | --- | --- |
| **Adherence to the TCD**  (lowest adherence T1 to highest adherence T4/T5)** | **T1** | **T2** | **T3** | **T4** | **T1** | **T2** | **T3** | **T4** |
| **Rice** | 194.5 (103.5) | 176 (92) | 239.5 (88.5) | 359.5 (165.5) | 68 (82) | 194.5 (103.5) | 261 (103.5) | 400.5 (182.5) |
| **Cereals** | 45.5 (85) | 17 (425.5) | 7.5 (24) | 6 (26) | 51.5 (88) | 20.5 (53.5) | 11.5 (36) | 5.5 (21) |
| **Wheat and Wheat products** | 143.5 (125.5) | 62.5 (62) | 44 (48.5) | 23.5 (38) | 183 (163.5) | 68.5 (68.5) | 60.5 (56.5) | 28.5 (45.5) |
| **Starchy and tuber** | - | - | - | - | - | - | - | - |
| **Dried legumes/soy foods** | - | - | - | - | - | - | - | - |
| **Fried food** | - | - | - | - | - | - | - | - |
| **Fruits** | - | - | - | - | - | - | - | - |
| **Vegetables** | 75 (83.5) | 100.5 (88) | 154.5 (98) | 231 (156.5) | 80.5 (84.5) | 100.5 (105.5) | 148.5 (93) | 230 (136.5) |
| **Cakes** | - | - | - | - | - | - | - | - |

**Table S9. (continued)**

| **Food Groups** | **Xu et al., 2015^S68^** for women** | | | | **Xu et al., 2015^S68^** for men** | | | |
| --- | --- | --- | --- | --- | --- | --- | --- | --- |
| **Adherence to the TCD**  (lowest adherence T1 to highest adherence T4/T5)** | **T1** | **T2** | **T3** | **T4** | **T1** | **T2** | **T3** | **T4** |
| **Animal meat** | 19.5 (27) | 37 (39.5) | 65.5 (45) | 113.5 (85) | 24.5 (33.5) | 40 (44) | 67.5 (49) | 110 (84) |
| **Poultry and its products** | 2.5 (10) | 5.5 (19) | 11.5 (27) | 25.5 (46) | 5.5 (18.5) | 6.5 (23) | 10.5 (27) | 24 (47) |
| **Pickled foods** | - | - | - | - | - | - | - | - |
| **Aquatic** | 6.5 (20.5) | 18 (38) | 34.5 (53) | 55.5 (87) | 9 (37.5) | 27 (48.5) | 39 (57.5) | 56 (79) |
| **Dairy products** | - | - | - | - | - | - | - | - |
| **Egg and its products** | - | - | - | - | - | - | - | - |
| **Soft drinks** | - | - | - | - | - | - | - | - |
| **Oil and fat** | 52.4 (178.7) | 37.3 (39.9) | 39.2 (28.5) | 53.9 (157) | 60.4 (219.8) | 45 (65.3) | 44.8 (45.5) | 52.5 (140.5) |

**Table S9. (continued)**

| **Food Groups** | **Shu et al. 2015^S53^**** | | | | **Shi et al. 2011^S50^***** | | | |
| --- | --- | --- | --- | --- | --- | --- | --- | --- |
| **Adherence to the TCD**  (lowest adherence T1 to highest adherence T4/T5)** | **T1** | **T2** | **T3** | **T4** | **T1** | **T2** | **T3** | **T4** |
| **Rice** | 282 (149.9) | - | - | 365.8 (188.2) | 123 (6) | 299 (6) | 382 (6) | 478 (6) |
| **Cereals** | 19.3 (20.8) | - | - | 36.2 (35.1) | 15 (1) | 5 (1) | 2 (1) | 3 (1) |
| **Wheat and Wheat products** | - | - | - | - | 298 (6) | 40 (6) | 20 (6) | 21 (6) |
| **Starchy and tuber** | 18.7 (17.5) | - | - | 28.9 (34.6) | 21 (1) | 10 (1) | 10 (1) | 9 (1) |
| **Dried legumes/soy foods** | - | - | - | - | - | - | - | - |
| **Fried food** | - | - | - | - | - | - | - | - |
| **Fruits** | 327.3 (169.1) | - | - | 457.8 (149.6) | 58 (5) | 44 (5) | 45 (5) | 80 (5) |
| **Vegetables** |  | - | - |  | 177 (8) | 225 (8) | 272 (8) | 365 (8) |
| **Cakes** | - | - | - | - | - | - | - | - |
| **Animal meat** | 69.6 (39.2) | - | - | 65.5 (30.2) | 21 (2) | 33 (2) | 43 (2) | 59 (2) |

**Table S9. (continued)**

| **Food Groups** | **Shu et al. 2015^S53^**** | | | | **Shi et al. 2011^S50^***** | | | |
| --- | --- | --- | --- | --- | --- | --- | --- | --- |
| **Adherence to the TCD**  (lowest adherence T1 to highest adherence T4/T5)** | **T1** | **T2** | **T3** | **T4** | **T1** | **T2** | **T3** | **T4** |
| **Poultry and its products** | - | - | - | - | - | - | - | - |
| **Pickled foods** | - | - | - | - | 18 (2) | 14 (2) | 16 (2) | 26 (2) |
| **Aquatic** | - | - | - | - | 24 (2) | 28 (2) | 30 (2) | 35 (2) |
| **Dairy products** | - | - | - | - | - | - | - | - |
| **Egg and its products** | - | - | - | - | - | - | - | - |
| **Soft drinks** | 17.2 (56.6) | - | - | 4.5 (20.8) | - | - | - | - |
| **Oil and fat** | - | - | - | - | 75 (2) | 80 (0) | 85 (2) | 87 (2) |

**Table S9. (continued)**

| **Food Groups** | **Shi et al. 2006^S49^**** | | | | **Qin et al. 2021^S43^** | | | | |
| --- | --- | --- | --- | --- | --- | --- | --- | --- | --- |
| **Adherence to the TCD**  (lowest adherence T1 to highest adherence T4/T5)** | **T1** | **T2** | **T3** | **T4** | **T1** | **T2** | **T3** | **T4** | **T5** |
| **Rice** | 87 (78) | - | - | 467 (143) | 240.9 | 240.2 | 238.2 | 146 | 58.9 |
| **Cereals** | 16 (31) | - | - | 4 (18) | 4 | 5.8 | 9.7 | 18.4 | 87.9 |
| **Wheat and Wheat products** | 377 (176) | - | - | 22 (34) | 6 | 10.8 | 27.2 | 57.2 | 69.9 |
| **Starchy and tuber** | - | - | - | - | - | - | - | - | - |
| **Dried legumes/soy foods** | - | - | - | - | 24.8 | 23.7 | 26 | 25.5 | 16.4 |
| **Fried food** | - | - | - | - | - | - | - | - | - |
| **Fruits** | 66 (66) | - | - | 72 (129) | 25.5 | 31.3 | 49.9 | 59.1 | 33 |
| **Vegetables** | 154 (105) | - | - | 375 (205) | 89.8 | 98.2 | 101.9 | 97.5 | 100.1 |
| **Cakes** | - | - | - | - | - | - | - | - | - |
| **Animal meat** | 33 (40) | - | - | 87 (78) | 75 | 88.9 | 109.3 | 101.4 | 55.8 |

**Table S9. (continued)**

| **Food Groups** | **Shi et al. 2006^S49^**** | | | | **Qin et al 2021^S43^** | | | | |
| --- | --- | --- | --- | --- | --- | --- | --- | --- | --- |
| **Adherence to the TCD**  (lowest adherence T1 to highest adherence T4/T5)** | **T1** | **T2** | **T3** | **T4** | **T1** | **T2** | **T3** | **T4** | **T5** |
| **Poultry and its products** | - | - | - | - | 21.7 | 18.3 | 23.1 | 20.4 | 7.2 |
| **Pickled foods** | - | - | - | - | 184.7 | 67.7 | 57.2 | 57.6 | 24.5 |
| **Aquatic** | - | - | - | - | 23.9 | 18.6 | 21.5 | 15.7 | 5 |
| **Dairy products** | 4 (26) | - | - | 26 (83) | 2.3 | 4.9 | 15.4 | 39.8 | 44 |
| **Egg and its products** |  | - | - | - | 24.4 | 33.6 | 49.7 | 55.9 | 72.1 |
| **Soft drinks** | - | - | - | - | - | - | - | - | - |
| **Oil and fat** | - | - | - | - | - | - | - | - | - |

**Table S9. (continued)**

| **Food Groups** | **Lu et al 2016^S35^**** | | | |
| --- | --- | --- | --- | --- |
| **Adherence to the TCD**  (lowest adherence T1 to highest adherence T4/T5)** | **T1** | **T2** | **T3** | **T4** |
| **Rice** | - | - | - | - |
| **Cereals** | 56 (101) | - | - | 85 (132) |
| **Wheat and Wheat products** | 219 (106) | - | - | 387 (295) |
| **Starchy and tuber** | - | - | - | - |
| **Dried legumes/soy foods** | 25 (124) | - | - | 23 (25) |
| **Fried food** | 9 (23) | - | - | 12 (22) |
| **Fruits** | 21 (46) | - | - | 19 (30) |
| **Vegetables** |  | - | - |  |
| **Cakes** | 1 (6) | - | - | 4 (11) |
| **Animal meat** | 373 (510) | - | - | 644 (648) |
| **Poultry and its products** | 25 (28) | - | - | 99 (206) |
| **Pickled foods** | 2 (5) | - | - | 8 (17) |
| **Aquatic** | 4 (7) | - | - | 21 (28) |
| **Dairy products** | 10 (14) | - | - | 45 (41) |
| **Egg and its products** | 25 (32) | - | - | 83 (172) |
| **Soft drinks** | 9 (47) | - | - | 4 (23) |
| **Oil and fat** | 19 (18) | - | - | 21 (19) |

TCD, Traditional Chinese diet.

*Results were expressed as median (range);**Results were expressed as mean (standard deviation); ***Results were expressed as mean (standard error).

1. **Amounts reported as daily consumed (grams/day)**

| **Food Groups** | **Leonetti et al. 2016^S22^** |
| --- | --- |
| **Milk** | 202.76 |
| **Bread** | 90 |
| **Ham** | 50 |
| **“Robiola” cheese** | 50 |
| **Beans** | 30.6 |
| **Shallot powder** | 3.3 |
| **Black sesame** | 3 |
| **Buckwheat flour** | 3 |
| **Corn flour/oat flour** | 3.6 |
| **Mushroom** | 1.2 |
| **Almond** | 1.2 |
| **Kelp** | 1.2 |
| **Salt** | 1.2 |
| **Soya** | 27 |
| **Vegetables** | 600-1000 |

**Supplementary materials I:**

**Table S10. Risk of bias assessment for review question 1 (reference list shown at the end of the supplementary material)**

| Name (Year) | Description of what food items are included in the dietary pattern? | Description of what food groups are included in the dietary pattern? | Description of proportions, quantities, or frequencies of foods included in the pattern? | Clear description of the methodology used to identify the pattern? | Description of what the geographical area(s) the dietary pattern was consumed in? | Description of sectors of the population represented? | Clear identification that the data/ descriptions of diet are nationally or regionally representative | Description of what year(s) data were collected? |  |  |
| --- | --- | --- | --- | --- | --- | --- | --- | --- | --- | --- |
| Cao et al. (2021)^S6^ | ⬤ | ⬤ | ⬤ | ⬤ | ⬤ | ⬤ | ⬤ | ⬤ |  |  |
| Cao et al. (2020a)^S4^ | ⬤ | ⬤ | ⬤ | ⬤ | ⬤ | ⬤ | ⬤ | ⬤ |  |  |
| Cao et al. (2020b)^S5^ | ⬤ | ⬤ | ⬤ | ⬤ | ⬤ | ⬤ | ⬤ | ⬤ |  |  |
| Batis et al. (2014) ^S1^ | ⬤ | ⬤ | ⬤ | ⬤ | ⬤ | ⬤ | ⬤ | ⬤ |  |  |
| Batis et al. (2016)^S2^ | ⬤ | ⬤ | ⬤ | ⬤ | ⬤ | ⬤ | ⬤ | ⬤ |  |  |
| Batis Ruvalcaba (2013)^S3^ | ⬤ | ⬤ | ⬤ | ⬤ | ⬤ | ⬤ | ⬤ | ⬤ |  |  |
| Chen (2005)^S7^ | ⬤ | ⬤ | ⬤ | ⬤ | ⬤ | ⬤ | ⬤ | ⬤ |  |  |
| Chen et al. (2017)^S8^ | ⬤ | ⬤ | ⬤ | ⬤ | ⬤ | ⬤ | ⬤ | ⬤ |  |  |
| Chen et al. (2020)^S9^ | ⬤ | ⬤ | ⬤ | ⬤ | ⬤ | ⬤ | ⬤ | ⬤ |  |  |
| Chung et al. (2018)^S10^ | ⬤ | ⬤ | ⬤ | ⬤ | ⬤ | ⬤ | ⬤ | ⬤ |  |  |
| Du et al. (2001)^S11^ | ⬤ | ⬤ | ⬤ | ⬤ | ⬤ | ⬤ | ⬤ | ⬤ |  |  |
| Fan et al. (2016)^S12^ | ⬤ | ⬤ | ⬤ | ⬤ | ⬤ | ⬤ | ⬤ | ⬤ |  |  |
| Fang (1999)^S13^ | ⬤ | ⬤ | ⬤ | ⬤ | ⬤ | ⬤ | ⬤ | ⬤ |  |  |
| Leonetti et al. (2016)^S22^ | ⬤ | ⬤ | ⬤ | ⬤ | ⬤ | ⬤ | ⬤ | ⬤ |  |  |
| Fu (2015)^S14^ | ⬤ | ⬤ | ⬤ | ⬤ | ⬤ | ⬤ | ⬤ | ⬤ |  |  |

**Table S10 (continued)**

| Name (Year) | Description of what food items are included in the dietary pattern? | Description of what food groups are included in the dietary pattern? | Description of proportions, quantities, or frequencies of foods included in the pattern? | Clear description of the methodology used to identify the pattern? | Description of what the geographical area(s) the dietary pattern was consumed in? | Description of sectors of the population represented? | Clear identification that the data/ descriptions of diet are nationally or regionally representative | Description of what year(s) data were collected? |
| --- | --- | --- | --- | --- | --- | --- | --- | --- |
| Guo et al. (2020)^S15^ | ⬤ | ⬤ | ⬤ | ⬤ | ⬤ | ⬤ | ⬤ | ⬤ |
| He et al. (2017)^S16^ | ⬤ | ⬤ | ⬤ | ⬤ | ⬤ | ⬤ | ⬤ | ⬤ |
| Hong et al. (2016)^S18^ | ⬤ | ⬤ | ⬤ | ⬤ | ⬤ | ⬤ | ⬤ | ⬤ |
| Hong et al. (2013)^S17^ | ⬤ | ⬤ | ⬤ | ⬤ | ⬤ | ⬤ | ⬤ | ⬤ |
| Hu et al. (2019)^S19^ | ⬤ | ⬤ | ⬤ | ⬤ | ⬤ | ⬤ | ⬤ | ⬤ |
| Lan et al. (2018)^S21^ | ⬤ | ⬤ | ⬤ | ⬤ | ⬤ | ⬤ | ⬤ | ⬤ |
| Li et al. (2021)^S27^ | ⬤ | ⬤ | ⬤ | ⬤ | ⬤ | ⬤ | ⬤ | ⬤ |
| Li (2011)^S23^ | ⬤ | ⬤ | ⬤ | ⬤ | ⬤ | ⬤ | ⬤ | ⬤ |
| Li et al. (2019)^S26^ | ⬤ | ⬤ | ⬤ | ⬤ | ⬤ | ⬤ | ⬤ | ⬤ |
| Li and Shi (2017)^S24^ | ⬤ | ⬤ | ⬤ | ⬤ | ⬤ | ⬤ | ⬤ | ⬤ |
| Li et al. (2022)^S28^ | ⬤ | ⬤ | ⬤ | ⬤ | ⬤ | ⬤ | ⬤ | ⬤ |
| Li et al. (2011)^S25^ | ⬤ | ⬤ | ⬤ | ⬤ | ⬤ | ⬤ | ⬤ | ⬤ |
| Liang (2022)^S29^ | ⬤ | ⬤ | ⬤ | ⬤ | ⬤ | ⬤ | ⬤ | ⬤ |
| Liao et al. (2019)^S30^ | ⬤ | ⬤ | ⬤ | ⬤ | ⬤ | ⬤ | ⬤ | ⬤ |
| Lin et al. (2019)^S31^ | ⬤ | ⬤ | ⬤ | ⬤ | ⬤ | ⬤ | ⬤ | ⬤ |
| Koo (1976)^S20^ | ⬤ | ⬤ | ⬤ | ⬤ | ⬤ | ⬤ | ⬤ | ⬤ |

**Table S10 (continued)**

| Name (Year) | Description of what food items are included in the dietary pattern? | Description of what food groups are included in the dietary pattern? | Description of proportions, quantities, or frequencies of foods included in the pattern? | Clear description of the methodology used to identify the pattern? | Description of what the geographical area(s) the dietary pattern was consumed in? | Description of sectors of the population represented? | Clear identification that the data/ descriptions of diet are nationally or regionally representative | Description of what year(s) data were collected? | | |
| --- | --- | --- | --- | --- | --- | --- | --- | --- | --- | --- |
| Liu et al. (2021a)^S33^ | ⬤ | ⬤ | ⬤ | ⬤ | ⬤ | ⬤ | ⬤ | ⬤ |  |  |
| Liu and Li (2000)^S32^ | ⬤ | ⬤ | ⬤ | ⬤ | ⬤ | ⬤ | ⬤ | ⬤ |  |  |
| Liu et al. (2021b)^S34^ | ⬤ | ⬤ | ⬤ | ⬤ | ⬤ | ⬤ | ⬤ | ⬤ |  |  |
| Lu et al. (2016)^S35^ | ⬤ | ⬤ | ⬤ | ⬤ | ⬤ | ⬤ | ⬤ | ⬤ |  |  |
| Lyu et al. (2014)^S36^ | ⬤ | ⬤ | ⬤ | ⬤ | ⬤ | ⬤ | ⬤ | ⬤ |  |  |
| Mi et al. (2017)^S38^ | ⬤ | ⬤ | ⬤ | ⬤ | ⬤ | ⬤ | ⬤ | ⬤ |  |  |
| Mu et al. (2014)^S39^ | ⬤ | ⬤ | ⬤ | ⬤ | ⬤ | ⬤ | ⬤ | ⬤ |  |  |
| Qi Mudedaoerji (2002)^S40^ | ⬤ | ⬤ | ⬤ | ⬤ | ⬤ | ⬤ | ⬤ | ⬤ |  |  |
| Qin et al. (2021)^S43^ | ⬤ | ⬤ | ⬤ | ⬤ | ⬤ | ⬤ | ⬤ | ⬤ |  |  |
| Qin et al. (2012) ^S41^ | ⬤ | ⬤ | ⬤ | ⬤ | ⬤ | ⬤ | ⬤ | ⬤ |  |  |
| Qin et al. (2014)^S42^ | ⬤ | ⬤ | ⬤ | ⬤ | ⬤ | ⬤ | ⬤ | ⬤ |  |  |
| Ru et al. (2021)^S44^ | ⬤ | ⬤ | ⬤ | ⬤ | ⬤ | ⬤ | ⬤ | ⬤ |  |  |
| Shen et al. (2021)^S46^ | ⬤ | ⬤ | ⬤ | ⬤ | ⬤ | ⬤ | ⬤ | ⬤ |  |  |
| Shen et al. (2020)^S45^ | ⬤ | ⬤ | ⬤ | ⬤ | ⬤ | ⬤ | ⬤ | ⬤ |  |  |
| Shi (2021)^S47^ | ⬤ | ⬤ | ⬤ | ⬤ | ⬤ | ⬤ | ⬤ | ⬤ |  |  |
| Shi and Ganji (2020)^S48^ | ⬤ | ⬤ | ⬤ | ⬤ | ⬤ | ⬤ | ⬤ | ⬤ |  |  |

**Table S10 (continued)**

| Name (Year) | Description of what food items are included in the dietary pattern? | Description of what food groups are included in the dietary pattern? | Description of proportions, quantities, or frequencies of foods included in the pattern? | Clear description of the methodology used to identify the pattern? | Description of what the geographical area(s) the dietary pattern was consumed in? | Description of sectors of the population represented? | Clear identification that the data/ descriptions of diet are nationally or regionally representative | Description of what year(s) data were collected? | |
| --- | --- | --- | --- | --- | --- | --- | --- | --- | --- |
| Shi et al. (2006)^S49^ | ⬤ | ⬤ | ⬤ | ⬤ | ⬤ | ⬤ | ⬤ | ⬤ |  |
| Shi et al. (2018)^S52^ | ⬤ | ⬤ | ⬤ | ⬤ | ⬤ | ⬤ | ⬤ | ⬤ |  |
| Shi et al. (2011)^S50^ | ⬤ | ⬤ | ⬤ | ⬤ | ⬤ | ⬤ | ⬤ | ⬤ |  |
| Shi et al. (2012)^S51^ | ⬤ | ⬤ | ⬤ | ⬤ | ⬤ | ⬤ | ⬤ | ⬤ |  |
| Shu et al. (2015)^S53^ | ⬤ | ⬤ | ⬤ | ⬤ | ⬤ | ⬤ | ⬤ | ⬤ |  |
| Shu et al. (2017)^S54^ | ⬤ | ⬤ | ⬤ | ⬤ | ⬤ | ⬤ | ⬤ | ⬤ |  |
| Sun et al. (2013)^S55^ | ⬤ | ⬤ | ⬤ | ⬤ | ⬤ | ⬤ | ⬤ | ⬤ |  |
| Tan et al. (2021)^S56^ | ⬤ | ⬤ | ⬤ | ⬤ | ⬤ | ⬤ | ⬤ | ⬤ |  |
| Tang et al. (2020)^S57^ | ⬤ | ⬤ | ⬤ | ⬤ | ⬤ | ⬤ | ⬤ | ⬤ |  |
| Wang et al. (2011)^S60^ | ⬤ | ⬤ | ⬤ | ⬤ | ⬤ | ⬤ | ⬤ | ⬤ |  |
| Wang et al. (2018)^S62^ | ⬤ | ⬤ | ⬤ | ⬤ | ⬤ | ⬤ | ⬤ | ⬤ |  |
| Wang et al. (2014)^S61^ | ⬤ | ⬤ | ⬤ | ⬤ | ⬤ | ⬤ | ⬤ | ⬤ |  |
| Wang et al. (2020a)^S63^ | ⬤ | ⬤ | ⬤ | ⬤ | ⬤ | ⬤ | ⬤ | ⬤ |  |
| Wang et al. (2020b)^S64^ | ⬤ | ⬤ | ⬤ | ⬤ | ⬤ | ⬤ | ⬤ | ⬤ |  |
| Wang et al. (2022)^S66^ | ⬤ | ⬤ | ⬤ | ⬤ | ⬤ | ⬤ | ⬤ | ⬤ |  |
| Wang et al. (2021)^S65^ | ⬤ | ⬤ | ⬤ | ⬤ | ⬤ | ⬤ | ⬤ | ⬤ |  |

**Table S10 (continued)**

| Name (Year) | Description of what food items are included in the dietary pattern? | Description of what food groups are included in the dietary pattern? | Description of proportions, quantities, or frequencies of foods included in the pattern? | Clear description of the methodology used to identify the pattern? | Description of what the geographical area(s) the dietary pattern was consumed in? | Description of sectors of the population represented? | Clear identification that the data/ descriptions of diet are nationally or regionally representative | Description of what year(s) data were collected? | | |  |
| --- | --- | --- | --- | --- | --- | --- | --- | --- | --- | --- | --- |
| Wang (1994)^S59^ | | ⬤ | ⬤ | ⬤ | ⬤ | ⬤ | ⬤ | ⬤ | ⬤ |  |  |
| Wei et al. (2018)^S67^ | | ⬤ | ⬤ | ⬤ | ⬤ | ⬤ | ⬤ | ⬤ | ⬤ |  |  |
| Xu et al. (2021)^S74^ | | ⬤ | ⬤ | ⬤ | ⬤ | ⬤ | ⬤ | ⬤ | ⬤ |  |  |
| Xu et al. (2020)^S73^ | | ⬤ | ⬤ | ⬤ | ⬤ | ⬤ | ⬤ | ⬤ | ⬤ |  |  |
| Xu et al. (2018b)^S72^ | | ⬤ | ⬤ | ⬤ | ⬤ | ⬤ | ⬤ | ⬤ | ⬤ |  |  |
| Xu et al. (2018a)^S71^ | | ⬤ | ⬤ | ⬤ | ⬤ | ⬤ | ⬤ | ⬤ | ⬤ |  |  |
| Xu et al. (2017)^S70^ | | ⬤ | ⬤ | ⬤ | ⬤ | ⬤ | ⬤ | ⬤ | ⬤ |  |  |
| Xu et al. (2015)^S68^ | | ⬤ | ⬤ | ⬤ | ⬤ | ⬤ | ⬤ | ⬤ | ⬤ |  |  |
| Xu et al. (2016)^S69^ | | ⬤ | ⬤ | ⬤ | ⬤ | ⬤ | ⬤ | ⬤ | ⬤ |  |  |
| Xue et al. (2017)^S75^ | | ⬤ | ⬤ | ⬤ | ⬤ | ⬤ | ⬤ | ⬤ | ⬤ |  |  |
| Yan et al. (2019)^S76^ | | ⬤ | ⬤ | ⬤ | ⬤ | ⬤ | ⬤ | ⬤ | ⬤ |  |  |
| Yang et al. (2015)^S77^ | | ⬤ | ⬤ | ⬤ | ⬤ | ⬤ | ⬤ | ⬤ | ⬤ |  |  |
| Tian et al. (2011)^S58^ | | ⬤ | ⬤ | ⬤ | ⬤ | ⬤ | ⬤ | ⬤ | ⬤ |  |  |
| Melaku et al. (2017)^S37^ | | ⬤ | ⬤ | ⬤ | ⬤ | ⬤ | ⬤ | ⬤ | ⬤ |  |  |
| Yu et al. (2015)^S78^ | | ⬤ | ⬤ | ⬤ | ⬤ | ⬤ | ⬤ | ⬤ | ⬤ |  |  |
| Yu et al. (2018)^S80^ | | ⬤ | ⬤ | ⬤ | ⬤ | ⬤ | ⬤ | ⬤ | ⬤ |  |  |

**Table S10 (continued)**

| Name (Year) | Description of what food items are included in the dietary pattern? | Description of what food groups are included in the dietary pattern? | Description of proportions, quantities, or frequencies of foods included in the pattern? | Clear description of the methodology used to identify the pattern? | Description of what the geographical area(s) the dietary pattern was consumed in? | Description of sectors of the population represented? | Clear identification that the data/ descriptions of diet are nationally or regionally representative | Description of what year(s) data were collected? |  |
| --- | --- | --- | --- | --- | --- | --- | --- | --- | --- |
| Yu et al. (2017)^S79^ | | ⬤ | ⬤ | ⬤ | ⬤ | ⬤ | ⬤ | ⬤ | ⬤ |
| Yu et al. (2022)^S81^ | | ⬤ | ⬤ | ⬤ | ⬤ | ⬤ | ⬤ | ⬤ | ⬤ |
| Zeng et al. (2013)^S82^ | | ⬤ | ⬤ | ⬤ | ⬤ | ⬤ | ⬤ | ⬤ | ⬤ |
| Zhang et al. (2017)^S86^ | | ⬤ | ⬤ | ⬤ | ⬤ | ⬤ | ⬤ | ⬤ | ⬤ |
| Zhang et al. (2015)^S83^ | | ⬤ | ⬤ | ⬤ | ⬤ | ⬤ | ⬤ | ⬤ | ⬤ |
| Zhang et al. (2021a)^S88^ | | ⬤ | ⬤ | ⬤ | ⬤ | ⬤ | ⬤ | ⬤ | ⬤ |
| Zhang et al. (2016a)^S84^ | | ⬤ | ⬤ | ⬤ | ⬤ | ⬤ | ⬤ | ⬤ | ⬤ |
| Zhang et al. (2020)^S87^ | | ⬤ | ⬤ | ⬤ | ⬤ | ⬤ | ⬤ | ⬤ | ⬤ |
| Zhang et al. (2021b)^S89^ | | ⬤ | ⬤ | ⬤ | ⬤ | ⬤ | ⬤ | ⬤ | ⬤ |
| Zhang et al. (2016b)^S85^ | | ⬤ | ⬤ | ⬤ | ⬤ | ⬤ | ⬤ | ⬤ | ⬤ |
| Zhao and Bao (2009)^S92^ | | ⬤ | ⬤ | ⬤ | ⬤ | ⬤ | ⬤ | ⬤ | ⬤ |
| Zhao and Bao (2004)^S91^ | | ⬤ | ⬤ | ⬤ | ⬤ | ⬤ | ⬤ | ⬤ | ⬤ |
| Zhao (2000)^S90^ | | ⬤ | ⬤ | ⬤ | ⬤ | ⬤ | ⬤ | ⬤ | ⬤ |
| Zheng et al. (2016)^S93^ | | ⬤ | ⬤ | ⬤ | ⬤ | ⬤ | ⬤ | ⬤ | ⬤ |
| Zhou et al. (2021)^S97^ | | ⬤ | ⬤ | ⬤ | ⬤ | ⬤ | ⬤ | ⬤ | ⬤ |
| Zhou et al. (2019a)^S95^ | | ⬤ | ⬤ | ⬤ | ⬤ | ⬤ | ⬤ | ⬤ | ⬤ |

**Table S10 (continued)**

| Name (Year) | Description of what food items are included in the dietary pattern? | | Description of what food groups are included in the dietary pattern? | | Description of proportions, quantities, or frequencies of foods included in the pattern? | Clear description of the methodology used to identify the pattern? | Description of what the geographical area(s) the dietary pattern was consumed in? | Description of sectors of the population represented? | Clear identification that the data/ descriptions of diet are nationally or regionally representative | Description of what year(s) data were collected? |
| --- | --- | --- | --- | --- | --- | --- | --- | --- | --- | --- |
| Zhou et al. (2019b)^S96^ | | ⬤ | | ⬤ | ⬤ | ⬤ | ⬤ | ⬤ | ⬤ | ⬤ |
| Zhou (1998)^S94^ | | ⬤ | | ⬤ | ⬤ | ⬤ | ⬤ | ⬤ | ⬤ | ⬤ |
| Zuo et al. (2013)^S98^ | | ⬤ | | ⬤ | ⬤ | ⬤ | ⬤ | ⬤ | ⬤ | ⬤ |
| Zuo et al. (2014)^S99^ | | ⬤ | | ⬤ | ⬤ | ⬤ | ⬤ | ⬤ | ⬤ | ⬤ |

**Supplementary materials I:**

**Table S11. Characteristics of the included studies for review question 2 (n=54) ( (reference list shown at the end of the supplementary material)**

| **First author/Year** | **Country** | **Assessment method used to define diet** | **Definition of diet** | **Participants characteristics (sample size, age, sex)** | **Comparator/Control** | **Results** | **Covariates** |
| --- | --- | --- | --- | --- | --- | --- | --- |
| **Case-control studies** | | | | | | | |
| Cao et al. (2021)^S6^ | China | Latent class analysis | TCD: Featured by the preference of white meat (as poultry) over red meat and the general willingness to take soya foods, with the lowest probabilities in non-consumption of the specific foods | 1499 women (cases: 695, controls: 804), mean age in cases: 55.07±11.27, mean age in controls: 54.42±11.28 | TCD (lowest quartile) | BC risk for all population:  OR: 0.86 (95% CI: 0.65 - 1.14);  for premenopausal (n= 556): OR: 0.76 (95% CI:0.47 - 1.23);  for postmenopausal (n= 943): OR: 0.90 (95% CI: 0.63 - 1.29) | Age, area, education, tobacco smoking, tea intake, alcohol intake, moderate physical activity, oral contraceptives use, hormone replacement therapy, family history of breast cancer, history of benign breast disease, age at menarche, parity, age at first full-term delivery, breast-feeding, height, BMI, energy intake, postmenopausal additional adjusted for the menopausal age |
| Lu et al. (2016)^S35^ | China | PCA | TCD: Mainly includes meat, vegetable/fruit, whole cereal (food made from rice, wheat flour and corn, etc.) and eggs | 1753 participants (case:818, control:935), mean age in case: 54.8±11.1. in control: 54.3±11.3, female:100% | TCD (lowest quartile) | BC risk: in menopausal women: OR:0.68 (95% CI: 0.48 - 0.97);  in pre-menopausal women: OR: 0.47 (95% CI: 0.29 - 0.76) | Age, education level, household income 5 years ago and residence |

**Table S11 (continued)**

| **First author/Year** | **Country** | **Assessment method used to define diet** | **Definition of diet** | **Participants characteristics (sample size, age, sex)** | **Comparator/Control** | **Results** | **Covariates** |
| --- | --- | --- | --- | --- | --- | --- | --- |
| **Case-control studies** | | | | | | | |
| Lan et al. (2018)^S21^ | China | PCA | Traditional Cantonese dietary pattern: Consisting of a high intake of fruit and vegetables, fish, Cantonese soup, and Chinese herb tea | 1564 participants (782 in case and 782 in control), mean age in case:52.71±11.27, in control:53.02±10.20, male:87% | Urban prudent dietary pattern, meat and preserved food pattern | Primary liver cancer risk:  OR: 0.61 (95% CI:0.46 - 0.82) | Sex, age, BMI, education level, income level, smoking, drinking, drink tea, physical activity, marital status, multivitamin use, and hypertension and diabetes status |
| Ru et al. (2021)^S44^ | China | Cluster analysis | TCD: Characterized by high intake of rice, congee, stuffed foods (i.e., dumplings), desserts, deep fried foods, poultry, and meats | 5145 participants (discovery cohort:2521, external validation cohort: 2624), mean age:53.6±13.8, male:41.5% | Mixed diet, high-alcohol diet | Association between T2DM and TCD was not found | Age, gender, educational level, physical activity, marital status, smoking status, BMI, and total energy intake |
| **Cohort studies** | | | | | | | |
| Batis et al. (2016)^S2^ | United States | PCA | Traditional southern diet: Positively related to intake of rice, high-fat pork, organ meats, poultry and game, and fish and seafood; and inversely associated with intake of wheat flour, wheat buns and breads, and corn and coarse grains | 4316 participants, mean age: 46.8±10.5, male: 45.6% | Traditional southern diet (lowest quartile) | Hba1c : % change:  −2.18 (95% CI : −3.34 - −1.01);  HOMA-IR : % change :  −10.34 (95% CI : −16.92 -−3.24);  Diabetes risk: OR: 0.86 (95% CI: 0.54 - 1.35) | Gender, age, geographic region, urbanicity scale, education level, income, smoking status, physical activity, alcohol intake, BMI |

**Table S11 (continued)**

| **First author/Year** | **Country** | **Assessment method used to define diet** | **Definition of diet** | **Participants characteristics (sample size, age, sex)** | **Comparator/Control** | **Results** | **Covariates** |
| --- | --- | --- | --- | --- | --- | --- | --- |
| **Cohort studies** | | | | | | | |
| Hong et al. (2013)^S17^ | China | PCA | TCD: Heavily loaded on leaf vegetables, rice, dry food, other vegetables, Aquatic products | 2093 participants, mean age:50.4±9.2, male:41.7% | TCD (lowest quartile) | HG risk in women:  RR: 0.59 (95%CI:0.35 - 0.99) | Age, education level, occupation, moderate PA, smoking, drinking and BMI |
| Cao et al. (2020b) ^S5^ | Australia | FA | TCD: Rice, pork, fish, poultry, and fresh vegetable were positively and heavily loaded while wheat, whole grain, and deep-fried products were negatively loaded | 6943 participants, age ＞ 50 years, male: 48.5% | TCD (low and rapid increase) | Decreased BMI in mean:  Β: -1.07 (95% CI: −1.33 - −0.81) | Urbanization levels: education level, smoking status, PA level, blood pressure, drinking, health status |

**Table S11 (continued)**

| **First author/Year** | **Country** | **Assessment method used to define diet** | **Definition of diet** | **Participants characteristics (sample size, age, sex)** | **Comparator/Control** | **Results** | **Covariates** |
| --- | --- | --- | --- | --- | --- | --- | --- |
| **Cohort studies** | | | | | | | |
| Li and Shi (2017) ^S24^ | Australia | PCA | TCD: Positively loaded for food groups of rice, pork, fish, poultry, tofu, and vegetables and negatively load with wheat, whole grains, and deep-fried food | 1991:11112 participants, mean age: 40.1±16.4, male: 48.6%; 1993:10816 participants, mean age:40.6 ±16.5, male;1997:11880 participants, mean age:41.8 ±16.7, male:50%; 2000:13312 participants, mean age:42.6 ±16.6, male: 48.9%; 2004:13183 participants, mean age:44.3 ±17.0, male:49%; 2006: 15911 participants, mean age:43.5±16.8, male:47.3%; 2009:16305 participants, mean age:44.5 ±16.9, male:46.7%; 2011:19721 participants, mean age: 46.0±16.6, male: 47% | TCD (lowest quartile) | Abdominal overweight/obesity:  Β: −0.3 3 (95%CI: −0.39 -−0.27);  Association (beta coef.) Between TCD and cardio metabolic profiles:  Β: −0.35 (95%CI: −0.37 - −0.32);  Hypertension:  Β: -0.15 (95%CI: −0.18 -  −0.12);  TG:  Β:0.03 (95%CI: −0.04 - 0.09);  Low HDL:  Β: −0.04 (95%CI: −0.11 - 0.02);  High LDL:  Β: −0.04 (95%CI: −0.10 - 0.02);  High cholesterol:  Β:0.01 (95%CI: −0.05 - 0.07);  Metabolic syndrome: β: −0.18 (95%CI: −0.25 - −0.12) | Urbanization, education level, annual family income, smoking status, physical activity level, alcohol consumption |
| Shi et al. (2011) ^S50^ | Australia | PCA | TCD: Loaded heavily on rice and fresh vegetables and inversely on wheat flour | 1231 participants, mean age:49.3, male:41.4% | TCD (lowest quartile) | Decreased weight in mean: −2·18 (95%CI: − 2.91 - −1.45) | Smoking, drinking, physical activity, inactivity, socio-economic status |

**Table S11 (continued)**

| **First author/Year** | **Country** | **Assessment method used to define diet** | **Definition of diet** | **Participants characteristics (sample size, age, sex)** | **Comparator/Control** | **Results** | **Covariates** |
| --- | --- | --- | --- | --- | --- | --- | --- |
| **Cohort studies** | | | | | | | |
| Xu et al. (2016) ^S69^ | Australia | PCA | TCD: Loaded heavily on rice, pork, and vegetables, and inversely on wheat flour and wheat buns | 6348 participants, aged ≥ 60 years | TCD (lowest quartile) | Decreased BMI in mean:  −0.23 (95%CI: −0.44 - −0.02);  Decrease WC in mean:  −1.57 (95%CI: −2.32 - −0.83);  Decrease weight in mean:  −0.90 (95%CI: −1.42 - −0.37) | Adjusted for age, urbanization, gender, marital status, work status, education level, smoking, physical activity, modern dietary pattern and energy, and other NCDs (known diabetes, myocardial infarction and stoke) |
| Chung et al. (2018)^S10^ | Australia | PCA | TCD: Characterized by soy products, processed gluten products, low nitrogen staple foods, low calorie desserts, seeds and nuts, root vegetables, starchy foods, pickled vegetables and tea | 785 participants, mean age: 59.5±8.9, male: 47.5% | TCD (lowest quartile) | Albuminuria risk:  OR: 1.02 (95%CI:0.82 - 1.26);  Kidney dysfunction:  OR: 0.81 (95%CI:0.63 - 1.06) | Age, sex, duration of diabetes, and education, smoking, drinking, BMI, hypertension, hba1c, serum triglycerides, |
| Qin et al. (2021) ^S43^ | China | PCA | Traditional northern diet: Featured with high intakes of wheat, other staples and egg, moderate intake of dairy products, and low intakes of rice and preserved vegetables | 477465 participants, mean age:51±10.5, male:40.9% | Traditional northern diet (lowest quartile) | Reduced risk of CVD: 8% (95%CI: 5 - 11%);  Reduced risk of HS:  12% (95%CI: 11 - 32%);  Reduced risk of IS:  14% (95%CI: 8 - 19%);  Reduced risk of diabetes: 15% (95%CI: 6 - 24%) | Age at recruitment, gender, and education level, smoking, alcohol drinking, spicy food consumption, and physical activity, diagnosed hypertension and diagnosed diabetes, family history of CVD and diabetes, and menopause status (only for women). |

**Table S11 (continued)**

| **First author/Year** | **Country** | **Assessment method used to define diet** | **Definition of diet** | **Participants characteristics (sample size, age, sex)** | **Comparator/Control** | **Results** | **Covariates** |
| --- | --- | --- | --- | --- | --- | --- | --- |
| **Cohort studies** | | | | | | | |
| Shi and Ganji (2020) ^S48^ | Australia | FA | TCD: Characterized by high intake of rice, pork, fish, and vegetables and low intake of wheat | 13055 participants, aged ≥20 years | TCD (lowest quartile) | CVD risk:  HR: 0.58 (95%CI: 0.42 - 0.79) | Age, gender, energy intake, intake of fat, smoking, alcohol drinking, income, urbanicity, education, physical activity, BMI, diabetes, and hypertension |
| Shi et al. (2012)^S51^ | Australia | PCA | TCD: Featured by rice, fresh vegetables and inversely on wheat flour | 1486 participants, aged ≥20 years, male:43.9% | TCD (lowest tertile) | Asthma risk: OR: 2.25(95%CI:1.45 - 3.51) | Age, gender, energy intake, smoking, alcohol drinking, income, manual job, overweight/obesity, |
| Xu et al. (2018b)^S72^ | Australia | FA | TCD: Loaded heavily on rice, pork and fish, and inversely on wheat and whole grain | 4847 participants aged ≥55 years, | TCD (lowest quartile) | Increased cognitive global scores: Β: 1.32 (95%CI:0.90 - 1.73), p＜0.001; Increased cognitive verbal memory scores: Β: 0.44 (95%CI:0.14 - 0.74), p=0.77 | Age, gender, urbanization index, marital status, work status, education levels, BMI, alcohol drinking, smoking status, survey year, and hypertension, diabetes |
| Yu et al. (2022) ^S81^ | China | PCA | Traditional northern diet: High intake of wheat and other staple foods, and a low intake of rice | 421426 participants, mean age: 50.64±10.28, male: 40.15% | Traditional northern diet (lowest quintile) | COPD:  HR: 0.91 (95%CI:0.79 - 1.05) | Sex, education level, marital status, household income, tobacco smoking, alcohol consumption, nutritional supplement, BMI, physical activity, daily energy intake, passive smoking, cook fuel pollution, and heat fuel pollution. |

**Table S11 (continued)**

| **First author/Year** | **Country** | **Assessment method used to define diet** | **Definition of diet** | **Participants characteristics (sample size, age, sex)** | **Comparator/Control** | **Results** | **Covariates** |
| --- | --- | --- | --- | --- | --- | --- | --- |
| **Cross-sectional studies** | | | | | | | |
| Cao et al. (2020a)^S4^ | China | PCA | TCD: Mainly including rice and flour, vegetables, and beans | 7555 participants, mean age: 51.27±14.79, male: 45.11% | TCD (lowest quartile) | Prediabetes risk:  OR: 1.057(95% CI: 0.933-1.199) | Gender, age, hypertension, dyslipidaemia, urban or rural, marriage, career, family history of Hypertension, family history of diabetes, alcohol drinker, and current smoker |
| Li et al. (2021)^S27^ | China | PCA | TCD: Heavily loaded on vegetables, staple foods and miscellaneous grains | 7604 participants, aged 45-59:50.3%, 60-79:49.7%, male:40.7% | TCD (lowest quartile) | Diabetes risk:  OR: 0.810(95%CI:0.690 - 0.952) | Age, gender, education level, occupation, monthly income, smoking and drinking status, and physical activity, BMI, occurrence of hypertension and hyperlipidaemia. |
| Shen et al. (2020)^S45^ | China | PCA | Traditional southern diet: High consumption of refined grains, vegetables, fruits, pickled vegetables, fish and shrimp, bacon and salted fish, salted and preserved eggs, milk, soya bean and its products, miscellaneous bean, fats and drinks | 1761 participants, aged between 45-59 years, male:52% | Traditional southern diet (lowest quartile) | Prediabetes risk:  OR: 1.17(95%CI:0.757 - 1.826) | Age, gender, income, education level, physical activity level, smoking status, hypertension, BMI, total energy intake |

**Table S11 (continued)**

| **First author/Year** | **Country** | **Assessment method used to define diet** | **Definition of diet** | **Participants characteristics (sample size, age, sex)** | **Comparator/Control** | **Results** | **Covariates** |
| --- | --- | --- | --- | --- | --- | --- | --- |
| **Cross-sectional studies** | | | | | | | |
| Shen et al. (2021)^S46^ | China | PCA | Traditional southern diet: high consumption of refined grains, vegetables, fruits, pickled vegetables, fish and shrimp, bacon and salted fish, salted and preserved eggs, milk, soya bean and its products, miscellaneous bean, fats and drinks | 1739 participants, median age:50(47-55), male:52.6% | Traditional southern diet (lowest quartile) | Glycaemic control (hba1c < 7·0 %):  OR: 1.08(95%CI:0.889 - 1.322) | Age, gender, income, education level, physical activity level, smoking status, hypertension, BMI, total energy intake |
| Shu et al. (2017)^S54^ | China | PCA | Traditional southern diet: High in refined grains, vegetables, fruits, pickled vegetables, fish and shrimp, bacon and salted fish, salted and preserved eggs, milk, soya bean and its products, miscellaneous bean, fats, and drinks | 1918 participants, aged between 45-59 years, male:51.9% | Traditional southern diet (lowest quartile) | T2dms risk:  OR: 1.09(95%CI:0.825 - 1.495) | Age, sex, education level, PA level, smoking status, BMI, total energy intake |
| Tan et al. (2021)^S56^ | China | PCA | Traditional northern diet: High intake of starchy roots and products, pork, pickled vegetables/dried vegetables, maize and desserts | 1861 participants, mean age:52.5, male:47.7% | Traditional northern diet (lowest quartile) | Diabetes risk:  OR: 0.811(95%CI:0.489 - 1.345) | Sex, age, residence, educational level, marital status, ethnic group, smoking status, abdominal obesity and dyslipidaemia |

**Table S11 (continued)**

| **First author/Year** | **Country** | **Assessment method used to define diet** | **Definition of diet** | **Participants characteristics (sample size, age, sex)** | **Comparator/Control** | **Results** | **Covariates** |
| --- | --- | --- | --- | --- | --- | --- | --- |
| **Cross-sectional studies** | | | | | | | |
| Zhou et al. (2019b)^S96^ | China | FA | TCD: High intakes of whole grains and related products, vegetables and fruit, beans and their products | 1744 participants, aged 40–60 years, male:59.2% | TCD (lowest quintile) | Diabetes risk:  OR: 1.20(95%CI:0.60 - 2.41) | Sex, age, education level, per capita income, marital status, residence place, smoking status, alcohol consumption, physical activities, obesity status, hypertension status, hypercholesterolaemia status and hypertriglyceridemia status |
| Zuo et al. (2013)^S98^ | China | PCA | TCD: High in eggs, tofu, organ meat and pickled vegetables | 1070 participants, aged ＞18 years, male:45.6% | TCD (lowest quartile) | Insulin resistance risk:  OR: 1.05 (95%CI:0.62 - 1.76) | Age, gender, income, energy intake, physical activity, smoking status and BMI |
| Xu et al. (2015)^S68^ | Australia | PCA | TCD: Loaded heavily on rice, pork, and vegetables, and inversely on wheat flour and wheat buns | 2745 participants, aged ≥ 60 years, male:47.4% | TCD (lowest quartile) | Prevalence of central obesity:  PR:0.80 (95%CI:0.64 - 0.97);  Prevalence of central obesity risk: PR:0.81 (95%CI:0.72 -0.90).  Decreased BMI:  Β: −0.41 (95% CI: −0.56- −0.27);  Decreased WC:  Β: −1.70 (95% CI: −2.12 -−1.29) | Age, marital status, work status, education level, smoking, physical activity, modern diet pattern, energy and urbanization levels |

**Table S11 (continued)**

| **First author/Year** | **Country** | **Assessment method used to define diet** | **Definition of diet** | **Participants characteristics (sample size, age, sex)** | **Comparator/Control** | **Results** | **Covariates** |
| --- | --- | --- | --- | --- | --- | --- | --- |
| **Cross-sectional studies** | | | | | | | |
| Li et al. (2022)^S28^ | China | FA | TCD: Consists of whole grains, vegetables, and legume products, emphasizing on various plant-based foods | 1136 participants, mean age:72±9.0, male:100% | TCD (lowest quartile) | Obesity/overweight risk:  OR: 0.51(95%CI:0.36 - 0.72);  Dyslipidaemia risk:  OR: 0.5 (95%CI:0.35 - 0.75);  HBP risk:  OR: 0.54 (95%CI:0.38 - 0.77);  IFG risk:  OR: 1.29 (95%CI:0.79 - 2.15);  Hyperuricemia risk:  OR: 0.67 (95%CI:0.47 - 0.97);  Decreased SMM: OR: 2.21(95%CI:1.36 -3 .16) | Age, living status, education level, smoking status, alcohol consumption and total energy intake |
| Shu et al. (2015)^S53^ | China | PCA | TCD: Characterized by high intakes of rice, steamed bun/noodles, coarse grains, tubers, fresh vegetables and fruits, fish and shrimp, miscellaneous beans and tea | 2560 participants, aged between 45-60 years, male: 59.3% | TCD (lowest quartile) | Central obesity risk: OR: 0.63 (95%CI:0.441 - 0.901) | Age, smoking status, economic income, educational level, physical activity and total energy intake |
| Wang et al. (2021)^S65^ | China | EFA and CFA | TCD: Loaded heavily on poultry, oils, condiment, light-coloured vegetables, dark-coloured vegetables, red meat and its products, cereals and tubers products | 1739 participants, aged over 25 years, male:46.2% | TCD (lowest quartile) | Obesity and obese risk:  OR in male: 1.954 (95%CI:1.258 - 3.036),  OR in female:1.114 (95%CI: 0.759 - 1.636) | Energy intake, age group, education level, job, smoking and income status |

**Table S11 (continued)**

| **First author/Year** | **Country** | **Assessment method used to define diet** | **Definition of diet** | **Participants characteristics (sample size, age, sex)** | **Comparator/Control** | **Results** | **Covariates** |
| --- | --- | --- | --- | --- | --- | --- | --- |
| **Cross-sectional studies** | | | | | | | |
| Tang et al. (2020)^S57^ | China | PCA | Traditional northern diet: Abundant in wheat and soybeans  Traditional southern diet: Rich in rice, vegetables, meat and poultry | 10863(north:4411, south:6452), aged 18-65 years, | Traditional northern diet (lowest quartile), traditional southern diet (lowest quartile) | Overweight risk: OR for northern diet: 1.33 (95%CI: not reported), for southern diet:1.17(95%CI: not reported);  Obesity: OR for northern diet: 1.33 (95%CI: not reported), for southern diet:0.98 (95%CI: not reported) | Household income, age, education level, sedentary time, parents’ histories of obesity and chronic health-related information |
| Yu et al. (2015)^S78^ | China | FA combined with cluster analysis | Traditional southern diet: Characterized by high intakes of rice but low intakes of wheat as staples  Traditional northern diet: Characterized by high intakes of wheat and other staples, but low intakes of rice, meat, poultry, fish and fresh fruit. | 474192 participants, aged 30-79 years, male:41% | Traditional northern diet (lowest quartile), traditional southern diet (lowest quartile) | Prevalence of general obese in traditional northern diet: PR:1.05 (95% CI:1.02 - 1.09);  Prevalence of central obese risk for traditional northern diet: PR:1.17 (95%CI:1.15 - 1.18) | Age, sex, study area, marital status, education level, household income, alcohol consumption, smoking status, and physical activity |
| Zhang et al. (2015) ^S83^ | China | PCA | Traditional southern diet: mainly includes rice, vegetables and pork  Traditional northern diet: Characterized by high intakes of wheat, other cereals and tubers | 2363 participants, aged 18-44 years, female:100% | Traditional southern diet (lowest quintile), traditional northern diet (lowest quintile) | General obesity risk in traditional southern diet: OR:0.48 (95%CI:0.29 - 0.78);  General obesity risk in traditional northern diet: OR:2.28 (95%CI:1.38 - 3.74);  Abdominal obesity risk in traditional southern diet: OR:0.64 (95%CI:0.46 - 0.90);  Abdominal obesity risk in traditional northern diet: OR:2.32 (95%CI:1.66 - 3.24) | Age, education, living area, annual household income per family member, physical activity, current smoker, current drinker and total energy intake |

**Table S11 (continued)**

| **First author/Year** | **Country** | **Assessment method used to define diet** | **Definition of diet** | **Participants characteristics (sample size, age, sex)** | **Comparator/Control** | **Results** | **Covariates** |
| --- | --- | --- | --- | --- | --- | --- | --- |
| **Cross-sectional studies** | | | | | | | |
| Zhang et al. (2016b)^S85^ | China | FA | TCD: Characterized by a high intake of wheat, cakes, and oil | 1604 participants, mean age:46.1±12.6, male:41.4% | TCD (lowest quartile) | General obesity risk:  OR: 1 (95%CI:0.56 - 1.81);  Central obesity risk:  OR: 0.92 (95%CI: 0.063 - 1.34) | Age, sex, energy intake, physical activity, smoking and alcohol intake |
| Shi et al. (2018)^S52^ | Australia | PCA | Traditional southern diet: Loaded heavily for rice, pork, and vegetable, with inverse loadings for wheat flour | 8429 participants, mean age:51±15, male:47.2% | Traditional southern diet (lowest quartile) | CKD risk:  OR :4.56 (95%CI:3.18 - 6.56) | Age, gender, energy intake, education, income, urbanization level, physical activity, smoking, and alcohol drinking. |
| Xu et al. (2020)^S73^ | China | PCA | Traditional southern diet: High intake of refined grains, vegetables, fruits, pickled vegetables, fish and shrimp, bacon and salted fish, salted and preserved eggs, milk, soyabean and its products, miscellaneous bean, fats, drinks | 2004 participants, aged 45–59 years, male:50% | Traditional southern diet (lowest quartile) | CKD risk:  OR: 0.94 (95%CI:0.78 - 1.11) | Age, gender, income, education level, physical activity level, smoking status, BMI, type 2 diabetes mellitus, hypertension and total energy intake |
| He et al. (2017)^S16^ | China | PCA | TCD: High intake of rice and rice products, coarse grains, starchy tubers, vegetables, pickled vegetables, pork, soybean and soybean products, and tea | 1204 participants, mean age: with hyperuricemia: 50.79±4.65, without hyperuricemia:51.21±4.69, male:61.7% | TCD (lowest quartile) | Hyperuricemia risk:  OR: 0.82 (95% CI:0.426 - 0.922) | Age, sex, education level, PA level, smoking status, BMI, total energy intake, alcohol use, hypertension |

**Table S11 (continued)**

| **First author/Year** | **Country** | **Assessment method used to define diet** | **Definition of diet** | **Participants characteristics (sample size, age, sex)** | **Comparator/Control** | **Results** | **Covariates** |
| --- | --- | --- | --- | --- | --- | --- | --- |
| **Cross-sectional studies** | | | | | | | |
| Shi (2021) ^S47^ | Australia | PCA | Traditional southern diet: High consumption of rice, pork and vegetable, but with inverse loadings for wheat flour and whole grain | 8429 participants, mean age:51±15, male:47.2% | Traditional southern diet (lowest quartile) | Hyperuricemia risk: OR: 3.24 (95%CI: 2.61 - 4.01) | Age, gender, intake of energy, education, income, urbanization level, smoking, alcohol drinking, physical activity, overweight/obesity, hypertension, and diabetes |
| Qin et al. (2014) ^S42^ | China | PCA | TCD: Characterized primarily by consumption of rice and freshly cooked vegetables, secondary of pork and fish, and lastly of root vegetable and wheat flour | 2518 participants, mean age:47.0±14.5, male:45.5% | TCD (lowest quartile) | Prevalence of hypertension:  PR:1.47 (95%CI:1.18 - 1.82) | Age, gender and other known risk factors including SES, salt and potassium intake, physical activity and alcohol use |
| Wang et al. (2020b) ^S64^ | China | PCA | Traditional northern diet: High intakes of starchy roots and products, pork, pickled vegetables/dried vegetables and corns | 1861 participants, mean age:52.45, male:47.77% | Traditional northern diet (lowest quartile) | Hypertension risk:  OR for male: 0.677 (95%CI: 0.452 - 1.014);  OR for female: 0.782 (95%CI: 0.511 -1.198) | Age place of residence, ethnicity, marital status, educational level, family history, dyslipidaemia, smoking status, physical activity, weight control, salt control, and BMI |

**Table S11 (continued)**

| **First author/Year** | **Country** | **Assessment method used to define diet** | **Definition of diet** | **Participants characteristics (sample size, age, sex)** | **Comparator/Control** | **Results** | **Covariates** |
| --- | --- | --- | --- | --- | --- | --- | --- |
| **Cross-sectional studies** | | | | | | | |
| Wang et al. (2022)^S66^ | China | FA | Traditional Jiangsu dietary pattern: Loaded heavily on rice, fruits, dark-coloured vegetables, light-coloured vegetables, animal meats, poultry, oil, and condiment | 4951 participants, aged over 18 years, male:44.7% | Traditional Jiangsu diet (lowest quartile) | High SBP risk:  OR: 1.095 (95%CI: 0.90  0 - 1.332);  High DBP risk:  OR: 0.877 (95%CI: 0.737 -1.043);  High TC risk:  OR: 1.001 (95%CI: 0.834 -1.201);  High TG risk:  OR: 0.940 (95%CI: 0.779 -1.133);  Low HDL-C risk:  OR: 1.395 (95%CI:1.067 -1.825);  High LDL-C risk:  OR: 0.938 (95%CI:0.783 -1.123);  High FPG risk:  OR: 0.870 (95%CI:0.728 -1.039); | Energy intake, gender, age, education, marital status, central obesity, smoking, drinking, BMI |
| Xu et al. (2018a)^S71^ | Australia | FA | TCD: High intake of rice, pork and vegetables | 2634 participants, median age in male: 67(63, 74), in female:68(63,74), male: 47.3% | TCD (lowest quartile) | Hypertension risk: RR: 0.69 (95% CI: 0.50 - 0.95) | Age, gender, marital status, work status, urbanization levels, education level, smoking, alcohol drinking, physical activity, modern diet pattern, energy, salt, lead exposure |

**Table S11 (continued)**

| **First author/Year** | **Country** | **Assessment method used to define diet** | **Definition of diet** | **Participants characteristics (sample size, age, sex)** | **Comparator/Control** | **Results** | **Covariates** |
| --- | --- | --- | --- | --- | --- | --- | --- |
| **Cross-sectional studies** | | | | | | | |
| Wang et al. (2011)^S60^ | China | PCA | Traditional northern diet: Characterized by high intakes of wheat flour products and starchy tubers, combined with low consumption of protein products such as pork, beef, poultry, aquatic products, or milk and milk products.  Traditional southern diet: Characterized by high intakes of fruit, pork, poultry, rice, vegetables, aquatic products and nuts | 23671 participants, mean age:39.8±0.1, male:53% | Traditional southern diet (lowest quartile), traditional northern diet (lowest quartile) | Newly diagnosed hypertension risk:  OR for traditional northern pattern:1.30 (95%CI:1.11 - 1.53);  OR for traditional southern diet: 0.73 (95%CI: 0.59 - 0.89) | Age, gender, living area, education level, family history of hypertension, income, PAL, alcohol consumption, total energy intake, and smoking status |
| Zheng et al. (2016)^S93^ | China | PCA | TCD: Characterized to have high loadings of foods such as rice, steamed bun/noodles, coarse grains, tubers, fresh vegetables and fruits, fish and shrimp, miscellaneous bean, and tea | 2560 participants, aged 45–60 years, male:52.2% | TCD (lowest quartile) | Prevalence of hypertension:  PR:1.14 (95%CI:0.76 - 1.652) | Gender, age, physical activity level, BMI and total energy intake |
| Liu and Li (2000)^S32^ | China | NR | Traditional Hakka diet: Mainly use of rice, fish, vegetables and fruit, and wide use of soybeans, extensive consumption o visceral organs; regularly eat fruits rich in dietary fibres | 200 participants, aged 35-54 | Diets in other cities (detailed information not reported) | Prevalence of hypertension in Hakka: lower than other cities;  The sodium/potassium ratio: lower than Guangzhou;  Mortality of CVD: lower than US/Canada | NA |

**Table S11 (continued)**

| **First author/Year** | **Country** | **Assessment method used to define diet** | **Definition of diet** | **Participants characteristics (sample size, age, sex)** | **Comparator/Control** | **Results** | **Covariates** |
| --- | --- | --- | --- | --- | --- | --- | --- |
| **Cross-sectional studies** | | | | | | | |
| Wang et al. (2014)^S61^ | China | PCA | TCD: High positive loadings on wheat, whole grains, fried pasta, pork, fresh vegetables, and pickled vegetables | 4968 participants， mean age:41.6±16.3, male:50.7% | TCD (lowest tertile) | Prevalence of hypertension:  23.9% (highest) | NA |
| Liu et al. (2021b)^S34^ | China | FA | Traditional Tianjin diet: High intake of animal liver, animal blood, seafood, sea-fish, freshwater fish | 2346 participants, mean age:59.7±6.29, male:55.1% | Traditional Tianjin diet (lowest quartile) | Carotid atherosclerosis risk:  OR in males:1.07 (95%CI:0.73 -1.56);  OR in females: 0.90 (95%CI: 0.61 - 1.33) | Age, sex, health status, drinking, smoking, PA level, depressive symptoms, education level, income level |
| Guo et al. (2020)^S15^ | China | PCA | TCD: High in grain, meat, pickled food, fried food, alcohol and salt while low in vegetables and fruits | 21472 participants, mean age in male:47.7±7.9, female:48.2± 8.3; male: 72.3% | TCD (lowest quartile) | High TC risk:  OR in male:1.10 (95%CI: 1.00 -1.21);  OR in female: 0.88 (95%CI:0.75 - 1.03)  High TG risk:  OR in male:1.21 (95%CI:1.10 -1.33);  OR in female: 0.96 (95%CI: 0.79 - 1.16);  Low HDL-C risk:  OR in male: 1.05 (95%CI:0.95 -1.17);  OR in female: 1.03 (95%CI: 0.88 - 1.22)  High LDL-C risk:  OR in male:1 (95%CI:0.91-1.09);  OR in female: 1.01(95%CI: 0.86 -1.19) | Age, BMI, WC, ethnicity, education, alcohol drinking status and smoking status |

**Table S11 (continued)**

| **First author/Year** | **Country** | **Assessment method used to define diet** | **Definition of diet** | **Participants characteristics (sample size, age, sex)** | **Comparator/Control** | **Results** | **Covariates** |
| --- | --- | --- | --- | --- | --- | --- | --- |
| **Cross-sectional studies** | | | | | | | |
| Lyu et al. (2014)^S36^ | China | PCA | TCD: Mainly includes cereal (food made from rice or wheat flour), potatoes (cassava, yams, taro, and so on), bean products, and vegetables | 8392 participants, mean age in male:53±14.9, in female:52±14.8, male:46.4% | TCD (lowest quartile) | High TG and HDL-C risk:  OR in male:0.77 (95%CI: 0.62 - 0.95);  OR in female:0.86 (95%CI: 0.69 - 1.07) | Age, HIL, residence, region, education, BMI, WC, smoking status, and alcohol drinking status |
| Zhang et al. (2016a)^S84^ | Australia | PCA | Traditional southern diet: High intakes of rice, pork, vegetables, and aquatic products and low consumption of wheat and other cereals | 2468 participants, aged 18-80 years, female:100% | Traditional southern diet (lowest quartile) | Association between traditional southern diet and HDL-C in female:  β: −1.86 (95% CI: −3.39 - −0.33) | Age, education level, living area, smoking status, drinking status, physical activity, annual household income, BMI and total energy intake |
| Li et al. (2011)^S25^ | China | FA combined with cluster analysis | Traditional northern diet: Characterized by high intakes of wheat flour products and (sweet) potato, combined with low consumption of protein products such as pork, beef, poultry, seafood, or milk and milk products.  Traditional southern diet: High intakes of rice, vegetables, seafood, pork, and poultry | 26276 participants aged ≥45 years, male in traditional southern dietary pattern:50.2%, male in traditional northern pattern: 46.1% | Western diet | Prevalence of stroke: traditional southern diet: 0.87%;  Traditional northern diet: 1.68%;  Stroke risk:  OR for traditional northern diet (compared to the traditional southern diet):1.82 (95%CI:1.36 – 2.43) | Age, sex, family income, educational level, current smoking, drinking, physical activity level, dietary salt, and salted vegetable consumption and family history of stroke |

**Table S11 (continued)**

| **First author/Year** | **Country** | **Assessment method used to define diet** | **Definition of diet** | **Participants characteristics (sample size, age, sex)** | **Comparator/Control** | **Results** | **Covariates** |
| --- | --- | --- | --- | --- | --- | --- | --- |
| **Cross-sectional studies** | | | | | | | |
| Lin et al. (2019)^S31^ | China | PCA | TCD: High consumption of vegetables, wheat products and red meat | 574 participants, age18–44：35.9%, 45–54:34.3%, ≥55:29.8%, male:55.4% | TCD (lowest tertile) | Endoscopic gastric mucosal atrophy:  OR: 1.00 (95%CI:0.59 - 1.70) | Age, gender, year of education, residence, marital status, occupation, income, BMI, smoking, drinking, H. Pylori infection, history of diabetes, history of hypertension, history of taking anticoagulants and total energy intake. |
| Shi et al. (2006) ^S49^ | Australia | PCA | TCD: Loaded heavily on rice and fresh vegetable and inversely on wheat flour | 2849 participants, aged ≥20 years, male:46% | TCD (lowest quartile) | Anaemia risk:  OR in male: 2.60 (95%CI:1.38 -4.88);  OR in female: 3.40 (95%CI:2.14 - 5.69) | Residence, age, socioeconomic status, region and education. |
| Wei et al. (2018) ^S67^ | China | PCA | TCD: High in whole grains, tubers, vegetables, fruit, pickled vegetables, mushrooms, bacon and salted fish, salted and preserved eggs, soyabean and its products, miscellaneous beans, vegetable oil and tea | 1918 participants, aged 45–59 years, male:51.69% | TCD (lowest quartile) | Metabolic syndrome risk:  OR:0.72 (95%CI:0.596 - 0.952) | Age, gender, education level, physical activity level, smoking status, total energy intake |
| Yang et al. (2015) ^S77^ | China | PCA | TCD: High intakes of staple food, coarse grains, fruits, eggs, fish and shrimp, milk, and tea | 999 participants, aged 45–60 years, male:46.5% | TCD (lowest quartile) | Prevalence of non-alcohol fatty liver disease risk:  PR: 0.837 (95%CI:0.660 -1.063) | Gender, age, physical activity, smoking status, BMI and blood pressure. |

**Table S11 (continued)**

| **First author/Year** | **Country** | **Assessment method used to define diet** | **Definition of diet** | **Participants characteristics (sample size, age, sex)** | **Comparator/Control** | **Results** | **Covariates** |
| --- | --- | --- | --- | --- | --- | --- | --- |
| **Randomised controlled study** | | | | | | | |
| Leonetti et al. (2016) ^S22^ | Italy | List foods in daily receipt | TCD: defined in terms of types of food and the calories contained in the three meals, including (breakfast, lunch and dinner, and adopted typical foods of traditional Chinese dietetics, including soybean, sesame, flour products, etc. | 284 participants, mean:47±8.7, male:39.4% | western diet | BMI decreased: 0.46 kg/m2 | NA |

BC, Breast cancer; BMI, Body mass index; CI, Confidence interval; CKD, Chronic kidney disease; COPD, Chronic obstructive pulmonary disease; CVD, Cardiovascular disease; DBP, Diastolic blood pressure; FA, Factor analysis; FPG, Fasting plasma glucose; HbA1c, Haemoglobin A1C; HDL-C, High-density lipoprotein cholesterol; HOMA-IR, Homeostatic model assessment insulin resistance; HR, Hazard ratio; HS, haemorrhagic stroke; IS, ischaemic stroke; LBM, Lean body mass; LDL-C Low-density lipoprotein cholesterol; MCE, Major coronary events; OR, Odds ratio; PA, Physical activity; PCA, Principal component analysis; PR, Prevalence ratio; RR, Relative risk; SBP, Systolic blood pressure; T2DM, Type 2 diabetes mellitus; TC Total cholesterol; TCD, Traditional Chinese diet; TG, Triglyceride; WC, Waist circumstance; Wt., Weight

**Supplementary materials I:**

**Table S12. a. Risk of bias for review question 2: case-control studies (11-point scale) (reference list shown at the end of the supplementary material)**

| Name (Year) | Adequate definition | Representativeness of cases | Selection of controls | Definitions of controls | Ascertainment of exposure | Comparability of cases and controls | Ascertainment of exposure | Same method of ascertainment for cases and controls | Non- responses rate | Overall |
| --- | --- | --- | --- | --- | --- | --- | --- | --- | --- | --- |
| Cao et al. (2021) ^S6^ | 0 | 1 | 1 | 1 | 0 | 1 | 0 | 0 | 0 | High risk |
| Lan et al. (2018)^S21^ | 0 | 1 | 0 | 1 | 2 | 2 | 1 | 2 | 0 | Low risk |
| Lu et al. (2016)^S35^ | 0 | 1 | 0 | 1 | 2 | 0 | 1 | 1 | 0 | Moderate risk |
| Ru et al. (2021)^S44^ | 1 | 1 | 1 | 1 | 2 | 1 | 1 | 1 | 0 | Low risk |

1. **Risk of bias for review question 2: cohort studies (9-point scale) (reference list shown at the end of the supplementary material)**

| Name (Year) | Representativeness of the exposed cohort | Selection of the non-exposed cohort | Ascertainment of exposure | Demonstration that outcome of interest was not present at start of study | Comparability | Assessment of outcome | Adequacy of follow-up period | Adequacy of follow up of cohorts | Overall |
| --- | --- | --- | --- | --- | --- | --- | --- | --- | --- |
| Batis et al. (2016) ^S2^ | 1 | 1 | 1 | 1 | 2 | 1 | 1 | 0 | Low risk |
| Cao et al. (2020b) ^S5^ | 1 | 1 | 1 | 1 | 2 | 1 | 1 | 0 | Low risk |
| Chung et al. (2018) ^S10^ | 0 | 0 | 1 | 1 | 1 | 1 | 1 | 1 | Moderate risk |
| Hong et al. (2013) ^S17^ | 1 | 1 | 1 | 1 | 1 | 1 | 1 | 0 | Low risk |

**Table S12 (continued)**

| Name (Year) | Representativeness of the exposed cohort | Selection of the non-exposed cohort | Ascertainment of exposure | Demonstration that outcome of interest was not present at start of study | Comparability | Assessment of outcome | Adequacy of follow-up period | Adequacy of follow up of cohorts | Overall |
| --- | --- | --- | --- | --- | --- | --- | --- | --- | --- |
| Li and Shi (2017) ^S24^ | 1 | 1 | 1 | 1 | 2 | 1 | 1 | 0 | Low risk |
| Qin et al. (2021) ^S43^ | 1 | 0 | 0 | 1 | 2 | 0 | 1 | 1 | Moderate risk |
| Shi and Ganji (2020)^S48^ | 1 | 0 | 0 | 1 | 2 | 0 | 1 | 0 | Moderate risk |
| Shi et al. (2011) ^S50^ | 1 | 1 | 1 | 0 | 1 | 1 | 1 | 1 | Low risk |
| Shi et al. (2012)^S51^ | 1 | 1 | 1 | 1 | 1 | 0 | 1 | 0 | Moderate risk |
| Xu et al. (2016) ^S69^ | 1 | 1 | 1 | 1 | 1 | 1 | 1 | 0 | Low risk |
| Xu et al. (2018b)^S72^ | 1 | 0 | 1 | 1 | 1 | 1 | 1 | 0 | Moderate risk |
| Yu et al. (2022)^S81^ | 1 | 0 | 1 | 1 | 1 | 1 | 1 | 0 | Moderate risk |

1. **Risk of bias for review question 2: cross-sectional studies (10-point scale) (reference list shown at the end of the supplementary material)**

| Name (Year) | Representativeness of the sample | Sample size | Non-respondents | Ascertainment of the exposure (risk factor) | Comparability | Assessment of outcome | Statistical test | Overall |
| --- | --- | --- | --- | --- | --- | --- | --- | --- |
| Cao et al. (2020a)^S4^ | 1 | 0 | 1 | 2 | 1 | 2 | 1 | Low risk |
| Guo et al. (2020)^S15^ | 1 | 0 | 0 | 2 | 0 | 1 | 1 | Moderate risk |
| He et al. (2017)^S16^ | 1 | 0 | 1 | 2 | 2 | 2 | 1 | Low risk |
| Li et al. (2011)^S25^ | 1 | 0 | 1 | 2 | 0 | 2 | 1 | Moderate risk |
| Li et al. (2021)^S27^ | 1 | 0 | 0 | 1 | 2 | 2 | 1 | Moderate risk |
| Li et al. (2022)^S28^ | 1 | 0 | 1 | 2 | 0 | 2 | 1 | Moderate risk |
| Lin et al. (2019)^S31^ | 0 | 0 | 1 | 2 | 2 | 2 | 1 | Low risk |
| Liu and Li (2000) ^S32^ | 1 | 0 | 0 | 0 | 0 | 2 | 0 | High risk |
| Liu et al. (2021b)^S34^ | 1 | 0 | 1 | 1 | 2 | 2 | 1 | Low risk |
| Lyu et al. (2014)^S36^ | 1 | 0 | 0 | 2 | 0 | 2 | 1 | Moderate risk |
| Qin et al. (2014) ^S42^ | 1 | 0 | 1 | 1 | 1 | 1 | 1 | Moderate risk |
| Shen et al. (2020)^S45^ | 0 | 0 | 1 | 2 | 1 | 2 | 1 | Moderate risk |
| Shen et al. (2021)^S46^ | 0 | 0 | 1 | 2 | 2 | 2 | 1 | Low risk |
| Shi (2021) ^S47^ | 1 | 0 | 1 | 2 | 2 | 2 | 1 | Low risk |
| Shi et al. (2006) ^S49^ | 0 | 1 | 1 | 2 | 0 | 2 | 1 | Moderate risk |
| Shi et al. (2018)^S52^ | 1 | 0 | 1 | 2 | 2 | 1 | 1 | Low risk |
| Shu et al. (2015)^S53^ | 0 | 0 | 1 | 1 | 1 | 1 | 1 | Moderate risk |

**Table S12 (continued)**

| Name (Year) | Representativeness of the sample | Sample size | Non-respondents | Ascertainment of the exposure (risk factor) | Comparability | Assessment of outcome | Statistical test | Overall |  |
| --- | --- | --- | --- | --- | --- | --- | --- | --- | --- |
| Shu et al. (2017)^S54^ | 1 | 0 | 1 | 2 | 2 | 2 | 1 | Low risk | |
| Tan et al. (2021)^S56^ | 1 | 1 | 0 | 2 | 1 | 1 | 1 | Moderate risk | |
| Tang et al. (2020)^S57^ | 1 | 0 | 0 | 2 | 1 | 2 | 1 | Moderate risk | |
| Wang et al. (2011)^S60^ | 1 | 0 | 0 | 2 | 2 | 2 | 1 | Low risk | |
| Wang et al. (2014) ^S61^ | 1 | 0 | 0 | 1 | 0 | 0 | 0 | High risk | |
| Wang et al. (2020b)^S64^ | 1 | 0 | 0 | 1 | 1 | 1 | 1 | Moderate risk | |
| Wang et al. (2021)^S65^ | 1 | 0 | 1 | 2 | 0 | 2 | 1 | Moderate risk | |
| Wang et al. (2022)^S66^ | 1 | 0 | 1 | 2 | 1 | 2 | 1 | Low risk | |
| Wei et al. (2018) ^S67^ | 1 | 0 | 1 | 2 | 0 | 2 | 1 | Moderate risk | |
| Xu et al. (2015) ^S68^ | 1 | 0 | 0 | 2 | 1 | 2 | 1 | Moderate risk | |
| Xu et al. (2018a)^S71^ | 1 | 0 | 1 | 2 | 1 | 2 | 1 | Low risk | |
| Xu et al. (2020)^S73^ | 0 | 0 | 1 | 2 | 2 | 2 | 1 | Low risk | |
| Yang et al. (2015) ^S77^ | 1 | 0 | 1 | 1 | 1 | 2 | 1 | Moderate risk | |
| Yu et al. (2015)^S78^ | 1 | 0 | 1 | 1 | 2 | 2 | 1 | Low risk | |
| Zhang et al. (2015) ^S83^ | 1 | 0 | 0 | 2 | 1 | 2 | 1 | Moderate risk | |
| Zhang et al. (2016a)^S84^ | 1 | 0 | 0 | 2 | 2 | 2 | 1 | Low risk | |
| Zhang et al. (2016b)^S85^ | 1 | 0 | 1 | 1 | 1 | 2 | 1 | Moderate risk | |

**Table S12 (continued)**

| Name (Year) | Representativeness of the sample | Sample size | Non-respondents | Ascertainment of the exposure (risk factor) | Comparability | Assessment of outcome | Statistical test | Overall |  |
| --- | --- | --- | --- | --- | --- | --- | --- | --- | --- |
| Zheng et al. (2016)^S93^ | 0 | 0 | 1 | 1 | 1 | 1 | 1 | Moderate risk | |
| Zhou et al. (2019b)^S96^ | 1 | 0 | 1 | 2 | 2 | 2 | 1 | Low risk | |
| Zuo et al. (2013)^S98^ | 1 | 0 | 1 | 2 | 1 | 2 | 1 | Low risk | |

1. **Risk of bias for review question 2: randomised controlled study(reference list shown at the end of the supplementary material)**

| Name (Year) | Randomization process | Deviations from intended interventions | Missing outcome data | Measurement of the outcome | Selection of the reported result | Overall |
| --- | --- | --- | --- | --- | --- | --- |
| Leonetti et al. (2016) ^S22^ | Low | No information | High | Some high | Low | High risk |

1. **Table S13. a. Quality reporting of observational studies, based on the STROBE 2007 checklist (n=53) (reference list shown at the end of the supplementary material)**

| **First author (year)** | **Items reported** | **Items partially reported** | **Items not reported** |
| --- | --- | --- | --- |
| Batis et al. (2016)^S2^ | 1,2,3,4,5,6,7,11,15,16,18,19,20,21,22 | 8,9,13,14,12 | 10,17 |
| Cao et al. (2020a)^S4^ | 1,2,3,4,6,7,9,11,15,16,17,18,19,21,22 | 5,8,12,13,14,20 | 10 |
| Cao et al. (2020b) ^S5^ | 1,2,3,4,5,6,7,11,16,17,18,19,21,22 | 8,9,12,14,15,20 | 10,13 |
| Cao et al. (2021)^S6^ | 1,2,3,4,5,11,13,15,16,17,18,19,20,21,22 | 6,7,8,11,12,14 | 9,10 |
| Chung et al. (2018) ^S10^ | 1,2,3,4,7,11,15,18,19,22 | 5,8,9,12,13,14,16,20,21 | 6,10,17 |
| Guo et al. (2020)^S15^ | 1,2,3,4,6,7,11,15,16,17,18,19,21,22 | 5,9,12,13,14,20 | 8,10 |
| He et al. (2017)^S16^ | 1,2,3,4,6,7,9,11,13,15,16,17,18,19,20,21,22 | 5,8,12,14 | 10 |
| Hong et al. (2013)^S17^ | 1,2,3,4,5,6,7,11,15,16,17,18 | 9,12,14,19,20 | 10,13,21,22 |

**Table S13 (continued)**

| **First author (year)** | **Items reported** | **Items partially reported** | **Items not reported** |
| --- | --- | --- | --- |
| Lan et al. (2018)^S21^ | 1,2,3,4, 7,8,11,14,15,16,17,18,19,20 | 5,60,12,13,21,22 | 9,10 |
| Li and Shi (2017)^S24^ | 1,2,3,4,5,7,11,12,13,15,16,17,18,19,20,22 | 8,9,14 | 10,21 |
| Li et al. (2011) ^S25^ | 1,2,3,4,6,7,9,15,16,17,18,19,20 | 5,11,12,14,21 | 8,10,13,22 |
| Li et al. (2021)^S27^ | 1,2,3,4,6,7,9,11,15,16,18,19 | 5,8,12,14,20 | 10,13,17,21,22 |
| Li et al. (2022)^S28^ | 1,2,3,4,6,7,9,11,13,15,16,17,18,19,20,22 | 5,12,14,21 | 8,10 |
| Lin et al. (2019)^S31^ | 1,2,3,4,7,9,11,12,13,14,15,16,18,19,20,22 | 5,8,21 | 6,10,17 |
| Liu and Li (2000)^S32^ | 2,18 | 1,3, 5,6,12,14,21 | 4,7,8,9,10,11,13,15,16,17,19,20,22 |
| Liu et al. (2021b)^S34^ | 1,2,3,4,6,7,8,9,11,13,15,16,18,19,20,21,22 | 5,12,14 | 10,17 |
| Lu et al. (2016)^S35^ | 1,2,3,4,7,8,9,11,13,14,15,16,18,19,20,21,22 | 5,6,7,12 | 10,17 |
| Lyu et al. (2014)^S36^ | 1,2,3,4,6,7,9,13,15,16,18,19,20,21 | 5,11,12,14 | 8,10,17,22 |
| Qin et al. (2014) ^S42^ | 1,2,3,4,5,6,7,15,16,18,19,21,22 | 8,9,11,12,13,14,20 | 10,17 |
| Qin et al. (2021) ^S43^ | 1,2,3,4,6,7,8,11,14,15,16,17,18,222 | 5,12,13,17,20,21 | 9,10 |
| Ru et al. (2021) ^S44^ | 1,2,3,4,6,7,11,15,16,18,19,20,21,22 | 5,8,12,13,14 | 9,10,17 |
| Shen et al. (2020)^S45^ | 1,2,3,4,7,9,13,15,16,18,19,20,21,22 | 5,6,8,11,12,14 | 10,17 |
| Shen et al. (2021)^S46^ | 1,2,3,4,7,9,13,15,16,18,19,20,21,22 | 5,6,8,11,12,14 | 10,17 |
| Shi (2021)^S47^ | 1,2,3,4,6,7,8,9,11,13,15,16,18,19,20,22 | 5,12,14,21 | 10,17 |
| Shi and Ganji (2020) ^S48^ | 1,2,3,4,5,6,7,8,11,13,15,16,17,18,19,20 | 12,14,21 | 9,10,22 |
| Shi et al. (2006) ^S49^ | 1,2,3,4,7,9,10,15,16,18,19,20,21,22 | 5,6,11,12,13,14 | 8,17 |

**Table S13 (continued)**

| **First author (year)** | **Items reported** | **Items partially reported** | **Items not reported** |
| --- | --- | --- | --- |
| Shi et al. (2011) ^S50^ | 1,2,3,4,5,6,9,11,15,16,18,19,20,21,22 | 7,9,12,13,14 | 8,10,17 |
| Shi et al. (2012)^S51^ | 1,2,3,4,5,6,7,11,13,15,16,17,18,19,20,21,22 | 8,12 | 9,10,14 |
| Shi et al. (2018)^S52^ | 1,2,3,4,6,7,9,11,12,13,14,15,16,17,18,19,20,22 | 5,9,21 | 10 |
| Shu et al. (2015)^S53^ | 1,2,3,4,7,11,13,14,15,16,17,18,19,20,21 | 5,6,8,9,12 | 10 |
| Shu et al. (2017)^S54^ | 1,2,3,4,6,7,8,9,13,15,16,18,19,20,21,22 | 5,11,12,14 | 10,17 |
| Tan et al. (2021)^S56^ | 1,2,3,4,6,7,11,15,16,18,19,20,22 | 5,8,9,12,13,14,21 | 10,17 |
| Tang et al. (2020)^S57^ | 1,2,3,4,6,7,9,11,13,15,16,17,18,19,20,21,22 | 5,8,12,14 | 10 |
| Wang et al. (2011)^S60^ | 1,2,3,4,6,7,8,9,11,13,15,16,17,18,19,20,21 | 5,12,14 | 10,22 |
| Wang et al. (2014)^S61^ | 1,2,3,4,6,15,18,19,20 | 5,7,11,12,13,14,16, | 8,10,17,21,22 |
| Wang et al. (2020b) ^S64^ | 1,2,3,4,6,7,11,13,15,16,17,18,19,20,22 | 5,8,9,12,14,21 | 10 |
| Wang et al. (2021)^S65^ | 1,2,3,4,6,7,11,13,14,15,18,19,20,21,22 | 5,12,16, | 8,10,17 |
| Wang et al. (2022)^S66^ | 1,2,3,4,6,7,9,11,15,16,17,18,19,20,21,22 | 5,8,12,13,14 | 10 |
| Wei et al. (2018) ^S67^ | 1,2,3,4,6,7,9,11,13,14,15,16,18,19,20,22 | 5,12,21 | 8,10,17 |
| Xu et al. (2015)^S68^ | 1,2,3,4,6,7,9,11,13,14,15,16,17,18,19,20,21,22 | 5,8,12 | 10 |
| Xu et al. (2016) ^S69^ | 1,2,3,4,5,6,7,11,13,15,16,18,19,20,21,22 | 8,9,12,17 | 10 |
| Xu et al. (2018a)^S71^ | 1,2,3,4,6,7,9,11,13,15,16,17,18,19,20,21 | 5,8,12,14 | 10,22 |

**Table S13 (continued)**

| **First author (year)** | **Items reported** | **Items partially reported** | **Items not reported** |
| --- | --- | --- | --- |
| Xu et al. (2018b)^S72^ | 1,2,3,4,5,6,7,11, 12,13,14,15,16,17,18,19,20,21 | 8,9,22 | 10 |
| Xu et al. (2020)^S73^ | 1,2,3,4,8,9,11,13,14,15,16,18,19,20,21,22 | 5,6,12 | 10,17 |
| Yang et al. (2015) ^S77^ | 1,2,3,4,6,7,9,13,14,15,16,18,19,20,22 | 5,11,12,21 | 10,17 |
| Yu et al. (2015)^S78^ | 1,2,3,4,5,6,7,8,9,11,13,14,15,16,17,18,19,20,21,22 | 12 | 10 |
| Yu et al. (2022)^S81^ | 1,2,3,4,5,7, 8,13,14,15,16,17,18,19,20,21,22 | 6 | 10 |
| Zhang et al. (2015) ^S83^ | 1,2,3,4,6,7,8,9,15,16,18,19,20,21 | 5,11, 12,14,22 | 10,13,17 |
| Zhang et al. (2016a)^S84^ | 1,2,3,4,6,7,8,9,11,15,16,18,19,20,21 | 5,11,13,14 | 10,17, 22 |
| Zhang et al. (2016b)^S85^ | 1,2,3,4,6,7,9,11,13,14,15,16,18,19,20,21 | 5,12,22,17 | 10 |
| Zheng et al. (2016) ^S93^ | 1,2,3,4,7,8,11,13,15,16,18,19,20,21,22 | 5,6,9,12,14,17 | 10 |
| Zhou et al. (2019b)^S96^ | 1,2,3,4,6,7,8,9,11,13,15,16,18,19,20,22 | 5,12,14 | 10,17 |
| Zuo et al. (2013)^S98^ | 1,2,3,4,6,7,9,11,13,15,16,18,19,20,21 | 5,8,12,14,17,22 | 10 |

1. **Quality reporting of randomised controlled trial, based on the CONSORT 2010 checklist (n=1)(reference list shown at the end of the supplementary material)**

| **First author (year)** | **Items reported** | **Items partially reported** | **Items not reported** |
| --- | --- | --- | --- |
| Leonetti et al. (2016) ^S22^ | 1a,2a,2b,3a,4a,4b,6a,8a,12a,13a,21 | 5,7b,10,17a,22 | 1b,3b,6b,7a,8b,9,12b,13b,14a,14b,15,16,18,19, 20 |

**Figure S1. Proportion of reported items in observational studies (n=53), based on the STROBE 2007 checklist**

**References (Supplementary I)**

S1. Batis C, Sotres-Alvarez D, Gordon-Larsen P, Mendez MA, Adair L, Popkin B. Longitudinal analysis of dietary patterns in Chinese adults from 1991 to 2009. *British Journal of Nutrition*. 2014;111(8):1441-1451

S2. Batis C, Mendez MA, Gordon-Larsen P, Sotres-Alvarez D, Adair L, Popkin B. Using both principal component analysis and reduced rank regression to study dietary patterns and diabetes in Chinese adults. *Public Health Nutr*. Feb 2016;19(2):195-203. doi:10.1017/s1368980014003103

S3. Batis Ruvalcaba C. *Dietary pattern trajectories over time and diabetes among Chinese adults*. 2013.

S4. Cao Y, Chen C, Cui L, et al. A population-based survey for dietary patterns and prediabetes among 7555 Chinese adults in urban and rural areas in Jiangsu Province. *Sci Rep*. Jun 26 2020;10(1):10488. doi:10.1038/s41598-020-67028-z

S5. Cao Y, Xu X, Shi Z. Trajectories of Dietary Patterns, Sleep Duration, and Body Mass Index in China: A Population-Based Longitudinal Study from China Nutrition and Health Survey, 1991-2009. *Nutrients*. Jul 27 2020;12(8)doi:10.3390/nu12082245

S6. Cao S, Lu S, Zhou J, et al. Association between dietary patterns and risk of breast cancer in Chinese female population: a latent class analysis. *Public Health Nutr*. Oct 2021;24(15):4918-4928. doi:10.1017/s1368980020004826

S7. Chen C. The challenge of introducing Western food to the traditional Chinese diet. Business and Economics 2005.

S8. Chen YC, Jung CC, Chen JH, et al. Association of Dietary Patterns With Global and Domain-Specific Cognitive Decline in Chinese Elderly. *J Am Geriatr Soc*. Jun 2017;65(6):1159-1167. doi:10.1111/jgs.14741

S9. Chen X, Ding Y, Shi L, et al. Dietary patterns and gestational hypertension in nulliparous pregnant Chinese women: A CONSORT report. *Medicine (Baltimore)*. Jul 17 2020;99(29):e20186. doi:10.1097/md.0000000000020186

S10. Chung HF, Hsu CC, Mamun AA, et al. Dietary patterns, dietary biomarkers, and kidney disease in patients with type 2 diabetes: a repeated-measure study in Taiwan. *Asia Pac J Clin Nutr*. 2018;27(2):366-374. doi:10.6133/apjcn.042017.15

S11. Du S, Lü B, Wang Z, Zhai F. [Transition of dietary pattern in China]. *Wei Sheng Yan Jiu*. Jul 2001;30(4):221-5.

S12. Fan R, Xu M, Wang J, et al. Sustaining Effect of Intensive Nutritional Intervention Combined with Health Education on Dietary Behavior and Plasma Glucose in Type 2 Diabetes Mellitus Patients. *Nutrients*. Sep 13 2016;8(9)doi:10.3390/nu8090560

S13. Fang L. The advantages and disadvatages of traditonal Chinese eating habits. Healthy lifestyle 1999.

S14. Fu K. Discussion the traditional Chinese eating habits and health. Yi Yuan Za Tan 2015.

S15. Guo Q, Ma Z, Zhu C, Zeng Q. Association of dietary pattern and physical activity with lipid-related indices among Chinese population: a cross-sectional study. *Lipids Health Dis*. Nov 23 2020;19(1):244. doi:10.1186/s12944-020-01420-6

S16. He F, Wang LL, Yu XL. Dietary patterns associated hyperuricemia among Chinese aged 45 to 59 years: An observational study. *Medicine (Baltimore)*. Dec 2017;96(50):e9248. doi:10.1097/md.0000000000009248

S17. Hong X, Li JQ, Wang ZY, Liang YQ, Yang HF, Xu F. [Dietary patterns and hyperglycemia in a follow-up study in Nanjing city]. *Zhonghua Yu Fang Yi Xue Za Zhi*. Feb 2013;47(2):135-41.

S18. Hong X, Ye Q, Wang Z, et al. Reproducibility and validity of dietary patterns identified using factor analysis among Chinese populations. *Br J Nutr*. Sep 2016;116(5):842-52. doi:10.1017/s000711451600249x

S19. Hu J, Oken E, Aris IM, et al. Dietary Patterns during Pregnancy Are Associated with the Risk of Gestational Diabetes Mellitus: Evidence from a Chinese Prospective Birth Cohort Study. *Nutrients*. Feb 15 2019;11(2)doi:10.3390/nu11020405

S20. Koo LC. Traditional Chinese diet and its relationship to health. *Kroeber Anthropological Society Papers*. 1976;48:116-147.

S21. Lan QY, Liao GC, Zhou RF, et al. Dietary patterns and primary liver cancer in Chinese adults: a case-control study. *Oncotarget*. Jun 12 2018;9(45):27872-27881. doi:10.18632/oncotarget.23910

S22. Leonetti F, Liguori A, Petti F, et al. Effects of basic traditional Chinese diet on body mass index, lean body mass, and eating and hunger behaviours in overweight or obese individuals. *J Tradit Chin Med*. Aug 2016;36(4):456-63. doi:10.1016/s0254-6272(16)30062-0

S23. Li K. On the Unique Traditional Catering of the Tujia Ethnic Group in Yanhe. *Journal of Nanning Polytechnic*. 2011;16(2):8-9. doi:1009-3621(2011)02-0007-06

S24. Li M, Shi Z. Dietary Pattern during 1991-2011 and Its Association with Cardio Metabolic Risks in Chinese Adults: The China Health and Nutrition Survey. *Nutrients*. Nov 6 2017;9(11)doi:10.3390/nu9111218

S25. Li Y, He Y, Lai J, et al. Dietary patterns are associated with stroke in Chinese adults. *The Journal of nutrition*. 2011;141(10):1834-1839.

S26. Li L, Huang A-P, Wang L-Q, Yu X-L. Empirically derived dietary patterns and constipation among a middle-aged population from China, 2016–2018. *Nutrition Journal*. 2019/12/26 2019;18(1):88. doi:10.1186/s12937-019-0512-9

S27. Li HJ, Li WL, Wu H, et al. [Dietary Patterns and Their Association with Diabetes Mellitus in Middle-Aged and Older Rural Population in Xinxiang County, Henan Province]. *Sichuan Da Xue Xue Bao Yi Xue Ban*. Jul 2021;52(4):662-670. doi:10.12182/20210760106

S28. Li T, Xie J, Shuai P, Huang J, He B. Dietary patterns, skeletal muscle mass loss, and cardiovascular risk among elderly men: A preliminary cross-sectional study in Sichuan province. *Environ Res*. May 15 2022;208:112719. doi:10.1016/j.envres.2022.112719

S29. Liang J. Trace Development History of Traditional Diet Standardization. *Food and Nutrition in China*. 2022;28(5):22-25.

S30. Liao K, Gu Y, Liu M, et al. Association of dietary patterns with depressive symptoms in Chinese postmenopausal women. *Br J Nutr*. Nov 28 2019;122(10):1168-1174. doi:10.1017/s0007114519001867

S31. Lin S, Gao T, Sun C, et al. Association of dietary patterns and endoscopic gastric mucosal atrophy in an adult Chinese population. *Sci Rep*. Nov 12 2019;9(1):16567. doi:10.1038/s41598-019-52951-7

S32. Liu XQ, Li YH. Epidemiological and nutritional research on prevention of cardiovascular disease in China. *Br J Nutr*. Dec 2000;84 Suppl 2:S199-203. doi:10.1079/096582197388699

S33. Liu Q, Cai J, Qin J, et al. Association between oil tea intake and the risk of type 2 diabetes in adults: A cross-sectional study in Gongcheng, Guangxi, China. *Asia Pac J Clin Nutr*. Sep 2021;30(3):487-496. doi:10.6133/apjcn.202109_30(3).0015

S34. Liu Y, Wang X, Zhang Q, et al. Relationship Between Dietary Patterns and Carotid Atherosclerosis Among People Aged 50 Years or Older: A Population-Based Study in China. *Front Nutr*. 2021;8:723726. doi:10.3389/fnut.2021.723726

S35. Lu S, Huang X, Yu H, et al. Dietary patterns and risk of breast cancer in Chinese women: a population-based case-control study. *The Lancet*. 2016/10/01/ 2016;388:S61. doi:<https://doi.org/10.1016/S0140-6736(16)31988-2>

S36. Lyu S, Su J, Xiang Q, Wu M. Association of dietary pattern and physical activity level with triglyceride to high-density lipoprotein cholesterol ratio among adults in Jiangsu, China: a cross-sectional study with sex-specific differences. *Nutr Res*. Aug 2014;34(8):674-81. doi:10.1016/j.nutres.2014.07.007

S37. Melaku YA, Gill TK, Appleton SL, Taylor AW, Adams R, Shi Z. Prospective Associations of Dietary and Nutrient Patterns with Fracture Risk: A 20-Year Follow-Up Study. *Nutrients*. Oct 31 2017;9(11)doi:10.3390/nu9111198

S38. Mi W, Lian W, Yang M, et al. [Analysis on dietary patterns and influencing factors among the elderly in Yantai City]. *Wei Sheng Yan Jiu*. Jan 2017;46(1):27-31.

S39. Mu M, Wang SF, Sheng J, et al. Dietary patterns are associated with body mass index and bone mineral density in Chinese freshmen. *J Am Coll Nutr*. 2014;33(2):120-8. doi:10.1080/07315724.2013.874897

S40. Qi Mudedaoerji. The traditional eating culture in Mongolian. *INNER MONGOLIA SOCIAL SCIENCES*. 2002;23(4):37-39. doi:10.14137/j.cnki .issn1003 -5281.

S41. Qin Y, Melse-Boonstra A, Yuan B, et al. Zinc biofortification of rice in China: a simulation of zinc intake with different dietary patterns. *Nutrients*. 2012;4(6):517-528.

S42. Qin Y, Melse-Boonstra A, Pan X, et al. Association of dietary pattern and body weight with blood pressure in Jiangsu Province, China. *BMC Public Health*. Sep 12 2014;14:948. doi:10.1186/1471-2458-14-948

S43. Qin C, Lv J, Yu C, et al. Dietary patterns and cardiometabolic diseases in 0.5 million Chinese adults: a 10-year cohort study. *Nutrition Journal*. 2021/09/03 2021;20(1):74. doi:10.1186/s12937-021-00730-4

S44. Ru Y, Wang N, Min Y, et al. Characterization of dietary patterns and assessment of their relationships with metabolomic profiles: A community-based study. *Clinical Nutrition*. 2021;40(5):3531-3541. doi:10.1016/j.clnu.2020.12.006

S45. Shen XM, Huang YQ, Zhang XY, Tong XQ, Zheng PF, Shu L. Association between dietary patterns and prediabetes risk in a middle-aged Chinese population. *Nutr J*. Jul 30 2020;19(1):77. doi:10.1186/s12937-020-00593-1

S46. Shen XM, Shu L, Huang YQ, Zhang XY, Zheng PF, Zhu Q. Association between dietary patterns and glycaemic control in a middle-aged Chinese population. *Public Health Nutr*. Sep 12 2021;25(8):1-9. doi:10.1017/s1368980021003931

S47. Shi Z. Cadmium Intake, Dietary Patterns and Hyperuricemia Among Adults in China. *Exposure and Health*. 2021/06/01 2021;13(2):219-227. doi:10.1007/s12403-020-00375-4

S48. Shi Z, Ganji V. Dietary patterns and cardiovascular disease risk among Chinese adults: a prospective cohort study. *Eur J Clin Nutr*. Dec 2020;74(12):1725-1735. doi:10.1038/s41430-020-0668-6

S49. Shi Z, Hu X, Yuan B, Pan X, Dai Y, Holmboe-Ottesen G. Association between dietary patterns and anaemia in adults from Jiangsu Province in Eastern China. *Br J Nutr*. Nov 2006;96(5):906-12. doi:10.1017/bjn20061785

S50. Shi Z, Yuan B, Hu G, Dai Y, Zuo H, Holmboe-Ottesen G. Dietary pattern and weight change in a 5-year follow-up among Chinese adults: results from the Jiangsu Nutrition Study. *Br J Nutr*. Apr 2011;105(7):1047-54. doi:10.1017/s0007114510004630

S51. Shi Z, Yuan B, Wittert GA, et al. Monosodium glutamate intake, dietary patterns and asthma in Chinese adults. *PloS one*. 2012;7(12):e51567.

S52. Shi Z, Taylor AW, Riley M, Byles J, Liu J, Noakes M. Association between dietary patterns, cadmium intake and chronic kidney disease among adults. *Clinical nutrition*. 2018;37(1):276-284.

S53. Shu L, Zheng PF, Zhang XY, et al. Association between Dietary Patterns and the Indicators of Obesity among Chinese: A Cross-Sectional Study. *Nutrients*. Sep 17 2015;7(9):7995-8009. doi:10.3390/nu7095376

S54. Shu L, Shen XM, Li C, Zhang XY, Zheng PF. Dietary patterns are associated with type 2 diabetes mellitus among middle-aged adults in Zhejiang Province, China. *Nutr J*. Dec 13 2017;16(1):81. doi:10.1186/s12937-017-0303-0

S55. Sun J, Buys N, Shen S. Dietary patterns and cardiovascular disease-related risks in Chinese older adults. *Frontiers in public health*. 2013;1:48.

S56. Tan S, Lu H, Song R, et al. Dietary quality is associated with reduced risk of diabetes among adults in Northern China: a cross-sectional study. *Br J Nutr*. Sep 28 2021;126(6):923-932. doi:10.1017/s0007114520004808

S57. Tang D, Bu T, Feng Q, Liu Y, Dong X. Differences in Overweight and Obesity between the North and South of China. *Am J Health Behav*. Nov 1 2020;44(6):780-793. doi:10.5993/ajhb.44.6.4

S58. Tian Y, Min J, Liu P, Sun J. [Clustering analysis on the dietary patterns of residents in Jiangsu Province]. *Wei Sheng Yan Jiu*. Nov 2011;40(6):771-2, 775.

S59. Wang Z. The Chinese dietary pattern. Dietary culture1994

S60. Wang D, He Y, Li Y, et al. Dietary patterns and hypertension among Chinese adults: a nationally representative cross-sectional study. *BMC Public Health*. Dec 14 2011;11:925. doi:10.1186/1471-2458-11-925

S61. Wang H, Deng F, Qu M, Yang P, Yang B. Association between Dietary Patterns and Chronic Diseases among Chinese Adults in Baoji. *Int J Chronic Dis*. 2014;2014:548269. doi:10.1155/2014/548269

S62. Wang CJ, Yang TF, Wang GS, Zhao YY, Yang LJ, Bi BN. Association between dietary patterns and depressive symptoms among middle-aged adults in China in 2016-2017. *Psychiatry Res*. Feb 2018;260:123-129. doi:10.1016/j.psychres.2017.11.052

S63. Wang J, Lin X, Bloomgarden ZT, Ning G. The Jiangnan diet, a healthy diet pattern for Chinese. *J Diabetes*. May 2020;12(5):365-371. doi:10.1111/1753-0407.13015

S64. Wang X, Liu A, Du M, et al. Diet quality is associated with reduced risk of hypertension among Inner Mongolia adults in northern China. *Public Health Nutr*. Jun 2020;23(9):1543-1554. doi:10.1017/s136898001900301x

S65. Wang Y-y, Tian T, Pan D, et al. The relationship between dietary patterns and overweight and obesity among adult in Jiangsu Province of China: a structural equation model. *BMC Public Health*. 2021/06/25 2021;21(1):1225. doi:10.1186/s12889-021-11341-3

S66. Wang YY, Zhang JX, Tian T, et al. Dietary patterns in association with the risk of elevated blood pressure, lipid profile and fasting plasma glucose among adults in Jiangsu Province of China. *Nutr Metab Cardiovasc Dis*. Jan 2022;32(1):69-79. doi:10.1016/j.numecd.2021.09.004

S67. Wei ZY, Liu JJ, Zhan XM, Feng HM, Zhang YY. Dietary patterns and the risk of metabolic syndrome in Chinese adults: a population-based cross-sectional study. *Public Health Nutr*. Sep 2018;21(13):2409-2416. doi:10.1017/s1368980018001088

S68. Xu X, Hall J, Byles J, Shi Z. Dietary Pattern Is Associated with Obesity in Older People in China: Data from China Health and Nutrition Survey (CHNS). *Nutrients*. Sep 23 2015;7(9):8170-88. doi:10.3390/nu7095386

S69. Xu X, Byles J, Shi Z, McElduff P, Hall J. Dietary pattern transitions, and the associations with BMI, waist circumference, weight and hypertension in a 7-year follow-up among the older Chinese population: a longitudinal study. *BMC Public Health*. Aug 8 2016;16:743. doi:10.1186/s12889-016-3425-y

S70. Xu X, Hall J, Byles J, Shi Z. Dietary pattern, serum magnesium, ferritin, C-reactive protein and anaemia among older people. *Clin Nutr*. Apr 2017;36(2):444-451. doi:10.1016/j.clnu.2015.12.015

S71. Xu X, Byles JE, Shi Z, Hall JJ. Dietary patterns, dietary lead exposure and hypertension in the older Chinese population. *Asia Pac J Clin Nutr*. 2018;27(2):451-459. doi:10.6133/apjcn.032017.20

S72. Xu X, Parker D, Shi Z, Byles J, Hall J, Hickman L. Dietary Pattern, Hypertension and Cognitive Function in an Older Population: 10-Year Longitudinal Survey. *Front Public Health*. 2018;6:201. doi:10.3389/fpubh.2018.00201

S73. Xu S-S, Hua J, Huang Y-Q, Shu L. Association between dietary patterns and chronic kidney disease in a middle-aged Chinese population. *Public Health Nutrition*. 2020;23(6):1058-1066. doi:10.1017/s1368980019002805

S74. Xu F, Chong BQ, Cai T, Zhang JL, Lv J. Associations between major dietary patterns and anxiety in middle-aged adults in eastern China. *Public Health Nutr*. May 2021;24(7):1716-1724. doi:10.1017/s1368980020000221

S75. Xue H, Tian G, Li D, Zhang J, Cheng G. The Relation of Dietary Pattern and Telomere Length among Adults in South China. *The FASEB Journal*. 2017;31:645.3-645.3.

S76. Yan H, Wu XY, Dang SN, Zhang YD, Luo SY. [Study on the association of dietary patterns of Shaanxi women of childbearing age during pregnancy with adverse pregnancy outcomes from 2010 to 2012]. *Zhonghua Yu Fang Yi Xue Za Zhi*. Aug 6 2019;53(8):829-834. doi:10.3760/cma.j.issn.0253-9624.2019.08.006

S77. Yang CQ, Shu L, Wang S, et al. Dietary Patterns Modulate the Risk of Non-Alcoholic Fatty Liver Disease in Chinese Adults. *Nutrients*. Jun 15 2015;7(6):4778-91. doi:10.3390/nu7064778

S78. Yu C, Shi Z, Lv J, et al. Major Dietary Patterns in Relation to General and Central Obesity among Chinese Adults. *Nutrients*. Jul 15 2015;7(7):5834-49. doi:10.3390/nu7075253

S79. Yu C, Shi Z, Lv J, et al. Dietary Patterns and Insomnia Symptoms in Chinese Adults: The China Kadoorie Biobank. *Nutrients*. Mar 4 2017;9(3)doi:10.3390/nu9030232

S80. Yu FN, Hu NQ, Huang XL, Shi YX, Zhao HZ, Cheng HY. Dietary patterns derived by factor analysis are associated with cognitive function among a middle-aged and elder Chinese population. *Psychiatry Res*. Nov 2018;269:640-645. doi:10.1016/j.psychres.2018.09.004

S81. Yu W, Pan L, Cao W, et al. Dietary Patterns and Risk of Chronic Obstructive Pulmonary Disease among Chinese Adults: An 11-Year Prospective Study. *Nutrients*. Feb 26 2022;14(5)doi:10.3390/nu14050996

S82. Zeng FF, Wu BH, Fan F, et al. Dietary patterns and the risk of hip fractures in elderly Chinese: a matched case-control study. *J Clin Endocrinol Metab*. Jun 2013;98(6):2347-55. doi:10.1210/jc.2013-1190

S83. Zhang JG, Wang ZH, Wang HJ, et al. Dietary patterns and their associations with general obesity and abdominal obesity among young Chinese women. *European Journal of Clinical Nutrition*. 2015/09/01 2015;69(9):1009-1014. doi:10.1038/ejcn.2015.8

S84. Zhang J, Wang Z, Wang H, et al. Association between dietary patterns and blood lipid profiles among Chinese women. *Public Health Nutr*. Dec 2016;19(18):3361-3368. doi:10.1017/s136898001600197x

S85. Zhang Q, Chen X, Liu Z, et al. Dietary Patterns in Relation to General and Central Obesity among Adults in Southwest China. *Int J Environ Res Public Health*. Nov 3 2016;13(11)doi:10.3390/ijerph13111080

S86. Zhang N, Du SM, Ma GS. Current lifestyle factors that increase risk of T2DM in China. *Eur J Clin Nutr*. Jul 2017;71(7):832-838. doi:10.1038/ejcn.2017.41

S87. Zhang M, Li Z, Yang S, et al. The Association between Dietary Patterns and Depressive Symptoms in Chinese Adults. *Biomed Res Int*. 2020;2020:8380151. doi:10.1155/2020/8380151

S88. Zhang J, Wang Z, Du W, et al. Twenty-Five-Year Trends in Dietary Patterns among Chinese Adults from 1991 to 2015. *Nutrients*. Apr 16 2021;13(4)doi:10.3390/nu13041327

S89. Zhang Q, Liu Z, Hu W, et al. Social capital and dietary patterns in three ethnic minority groups native to Yunnan Province, Southwest China. *Plos one*. 2021;16(8):e0256078.

S90. Zhao L. The characteristics of traditional dietary pattern. Nutrition in China 2000.

S91. Zhao L, Bao S. The advantages and limitations of the traditional Chinese dietary pattern. *Journal of Chinese Institute of Food Science and Technology*. 2004;4(4):12-16. doi:DOI:10.16429/j.1009-7848.2004.04.001

S92. Zhao L, Bao S. Adherence to the traditional Chinese diet is an important method to improvie the public health. Nutrition in China 2009.

S93. Zheng PF, Shu L, Zhang XY, et al. Association between Dietary Patterns and the Risk of Hypertension among Chinese: A Cross-Sectional Study. *Nutrients*. Apr 23 2016;8(4):239. doi:10.3390/nu8040239

S94. Zhou S. Promote the traditional food patterns. Food Science, Technology and Economics 1998.

S95. Zhou J, Sheng J, Fan Y, et al. The effect of Chinese famine exposure in early life on dietary patterns and chronic diseases of adults. *Public Health Nutr*. Mar 2019;22(4):603-613. doi:10.1017/s1368980018003440

S96. Zhou J, Sheng J, Fan Y, Zhu X, Wang S. Dietary patterns, dietary intakes and the risk of type 2 diabetes: results from the Hefei Nutrition and Health Study. *Int J Food Sci Nutr*. Jun 2019;70(4):412-420. doi:10.1080/09637486.2018.1515184

S97. Zhou C, Li M, Liu L, Zhao F, Cong W, Zhang F. Food Consumption and Dietary Patterns of Local Adults Living on the Tibetan Plateau: Results from 14 Countries along the Yarlung Tsangpo River. *Nutrients*. Jul 17 2021;13(7)doi:10.3390/nu13072444

S98. Zuo H, Shi Z, Yuan B, et al. Dietary patterns are associated with insulin resistance in Chinese adults without known diabetes. *Br J Nutr*. May 2013;109(9):1662-9. doi:10.1017/s0007114512003674

S99. Zuo H, Shi Z, Dai Y, et al. Serum leptin concentrations in relation to dietary patterns in Chinese men and women. *Public Health Nutr*. Jul 2014;17(7):1524-30. doi:10.1017/s1368980013001535
